# Knockout of phytoene desaturase gene using CRISPR/Cas9 in highbush blueberry

**DOI:** 10.3389/fpls.2022.1074541

**Published:** 2022-12-15

**Authors:** Giuseppe Vaia, Vera Pavese, Andrea Moglia, Valerio Cristofori, Cristian Silvestri

**Affiliations:** ^1^ Dipartimento di Scienze Agrarie e Forestali (DAFNE), Università della Tuscia, Viterbo, Italy; ^2^ Dipartimento di Scienze Agrarie, Forestali e Alimentari (DISAFA), Università degli Studi di Torino, Grugliasco, Italy

**Keywords:** *Vaccinium corymbosum* L., gene editing, phytoene desaturase, gene loss-of-function, *de novo* shoot organogenesis

## Abstract

Among the New Plant Breeding Techniques (NPBTs), the CRISPR/Cas9 system represents a useful tool for target gene editing, improving the traits of the plants rapidly. This technology allows targeting one or more sequences simultaneously, as well as introducing new genetic variations by homology-directed recombination. However, the technology of CRISPR/Cas9 remains a challenge for some polyploid woody species, since all the different alleles for which the mutation is required must be simultaneously targeted. In this work we describe improved protocols adapting the CRISPR/Cas9 system to highbush blueberry (*Vaccinium corymbosum* L.), using *Agrobacterium*-mediated transformation. As a proof of concept, we targeted the gene encoding for *phytoene desaturase*, whose mutation disrupts chlorophyll biosynthesis allowing for the visual assessment of knockout efficiency. Leaf explants of *in vitro*-cultured blueberry cv. Berkeley has been transformed with a CRISPR/Cas9 construct containing two guide RNAs (gRNA1 and gRNA2) targeting two conserved gene regions of *pds* and subsequently maintained on a selection medium enriched with kanamycin. After 4 weeks in culture on the selection medium, the kanamycin-resistant lines were isolated, and the genotyping of these lines through Sanger sequencing revealed successful gene editing. Some of mutant shoot lines included albino phenotypes, even if the editing efficiencies were quite low for both gRNAs, ranging between 2.1 and 9.6% for gRNA1 and 3.0 and 23.8 for gRNA2. Here we showed a very effective adventitious shoot regeneration protocol for the commercial cultivar of highbush blueberry “Berkeley”, and a further improvement in the use of CRISPR/Cas9 system in *Vaccinium corymbosum* L., opening the way to the breeding mediated by biotechnological approaches.

## Introduction

Blueberry plants are species with expanding production worldwide, and a commercially valuable minor fruit crop in Italy (especially highbush blueberry) since it is a much sought-after species to differentiate crops, utilizes marginal areas and meets the growing consumer demand (Marangelli et al., 2022)., Moreover it is interesting for both fresh market or to be processed as a food ingredients but also for pharmaceutical and cosmetics industries, since it represents an important source of antioxidants.

Blueberry fruits are used for fresh consumption to process for juices, preserves and jams, and even for the pharmaceutical and cosmetics industries, since they are one of the richest sources of antioxidant metabolites which possess a high potential to reduce the incidence of several degenerative diseases (Ferrao et al., 2021). The genetic engineering in blueberry has also been studied in depth, and many successful works have been reported describing novel transgenic traits such as freezing-tolerance obtained by the overexpression of *Vcddf1* gene (Walworth and Song, 2018), trans-grafting by an induced constitutive expression of *flowering locus T* gene (Song et al., 2019), increase of blueberry yield obtained by manipulating the expression of *Soc1*-MADS-Box (Song and Chen, 2018), and an interesting transgenic line which overexpresses the gene *Vcrr2*, useful to study the dormancy in highbush blueberry (Lin et al., 2019; Surridge, 2019).

Among the New Plant Breeding Techniques (NPBTs), the CRISPR/Cas9 has become part of the commonly used methodologies for obtaining new varieties (Bortesi and Fischer, 2015; Kim et al., 2021), has the advantage of accelerating and enhancing classical breeding, and it could be very interesting also for genetic improvement of highbush blueberry. Very little information about CRISPR/Cas9 and *V. corymbosum* L. has been reported. The first work reported the knockout of the *Centroradialis* (*Cen*) gene (Omori et al., 2021), and the second one studied the editing efficiency of CRISPR/Cas9 and CRISPR/Cas12a, both driven by a 35S promoter or AtUbi promoter, on a marker gene (*gusA*), previously introduced in transgenic lines as a single copy (Han et al., 2022). The last work demonstrated that the Cas-12a was not able to edit and that the factors to be considered during the process are numerous (pH, tissue culture conditions, temperature, etc.). Furthermore, highbush blueberry is a polyploid crop; traditional breeding often takes advantage of polyploidy. However, some methods such as random mutation breeding and genome editing are more difficult to be applied due to the presence of multiple homoeologous copies (or alleles) of each gene (Schaart et al., 2021). Hence, in polyploid species such as *Vaccinium corymbosum* L., guide RNAs must be carefully designed, since they must target all the different alleles simultaneously for which the mutation is required (Schaart et al., 2021). Therefore the application of editing technologies has also already been applied to many polyploid crops, including herbaceous and woody species (Zaman et al., 2019; Schaart et al., 2021). The number of new manuscripts published increased rapidly (Corte et al., 2019; Zhou et al., 2020; Fizikova et al., 2021; Karmakar et al., 2022; Liu et al., 2022; Nerkar et al., 2022; Parsaeimehr et al., 2022; Rao et al., 2022).

*Phytoene desaturase* (*pds*), a gene involved in carotenoid biosynthesis, has been targeted in most gene editing studies. *Pds* may be used as a visible marker to demonstrate specific genome editing effects (Savadi et al., 2021) since its knockout in plants results in albino phenotypes due to the absence of chlorophylls. CRISPR/Cas9-mediated *pds* gene knock-out has been found to confer an albino phenotype in many fruit crops such as grape (Nakajima et al., 2017), chestnut (2022; Pavese et al., 2021), strawberry (Wilson et al., 2019), cavendish banana (Naim et al., 2018; Ntui et al., 2020), apple (Nishitani et al., 2016) and pear (Charrier et al., 2019; Malabarba et al., 2021).

In this study, we reported the establishment of a CRISPR/Cas9-based transformation protocol through *de novo* shoot organogenesis in highbush blueberry by targeting the *Vcpds* endogenous reporter gene as a proof-of-concept of gene editing. The methodology here reported represents a suitable platform for the application of CRISPR/Cas9 technology in highbush blueberry.

## Materials and methods

### Plant material

The plant material used in our study were shoots of the cv Berkeley, cultivated on proliferation medium (PM) consisting of WPM basal medium including vitamins (Lloyd and McCown, 1980), 0.2 mgL^-1^ of L-glutamine, 3% (w/v) sucrose 3%, and 2.28 µM of zeatin trans-isomers (Duchefa, NL) (pH 5.6, gelled with 0.7% w/v agar - B&V, Italy) and grown under a 16 h photoperiod, provided by Fluora L58W/77 lamps (Osram, Germany), at photosynthetic photon lux (PPF) of 40 μmol m^−2^ s^−1^ and at 24 ± 1°C.

### Mining of pds in the Vaccinium genome and phylogenetic analysis

*Vaccinium myrtillus pds* (*Vmpds*) coding sequence was detected from the Genome Database for *Vaccinium* (GDV; https://www.vaccinium.org/ accessed on 1^st^ december 2021). The PROSITE database (https://prosite.expasy.org/ accessed on 20^th^ january 2022) was then used to analyze and annotate *Vmpds* functional domains. Subsequently, using NGPhilogeny online tool (https://ngphylogeny.fr, accessed on 31^st^ july 2022), the *Vmpds* coding sequence was aligned using MAFFT Alignment with 44 other plant *pds* coding sequence (**Supplementary File S1**), available on NCBI database, to produce the phylogenetic tree. The statistical significance of each branch was achieved by bootstrap analysis with 1,000 iterations. The phylogenetic tree was then visualized using PRESTO (Phylogenetic tReE viSualisaTiOn https://ngphylogeny.fr/data/displaytree, accessed on 31^st^ January 2022) tool.

### Vector design

To knock-out the *Vcpds* gene sequence, two specific guide RNA (gRNAs) (**Table 1**) were designed on the CDS of *Vaccinium myrtillus pds*, available on the Genome Database for *Vaccinium*, using CRISPR-P 2.0 online tool (http://crispr.hzau.edu.cn/CRISPR2, accessed on 31^st^ january 2022).

**Table 1:** Selected gRNA sequences from *pds* of *Vaccinium corymbosum* L.

gRNA	Score	Sequence	Strand	Position (bp)	%GC
gRNA1	0.6927	GGACTACTGCCAGCAATGATCGG	+	545	50%
gRNA2	0.6326	GAGAACTTGGTATAAATGATCGG	+	357	30%

Two pairs of primers (**Table 2**) designed on *V. myrtillus pds* gene were used to amplify *V. corymbosum pds* cv Berkeley to confirm the gRNAs sequences conservation among species.

**Table 2:** Primer sequences used for PCR of *pds* of *Vaccinium corymbosum* L. and for gRNAs flanking regions.

Primer	Sequence
***Vcpds*-F**	*5’*- ACATGCTTTAGGGTGGTGGT - *3’*
***Vcpds*-R**	*3’*- GTTCAATGCCTTTGACATGG - *5’*
**gRNA1-F**	*5’*- ATTGAGAACTTGGTATAAATGAT - *3’*
**gRNA1-R**	*3’*- AAACATCATTTATACCAAGTTCT - *5’*
**gRNA2-F**	*5’*- ATTGGACTACTGCCAGCAATGAT - *3’*
**gRNA2-R**	*3’*- AAACATCATTGCTGGCAGTAGTC - *5’*

The gRNAs were then assembled into a CRISPR/Cas9 vector using the GoldenBraid 4.0 assembly system (Vázquez-Vilar et al., 2021). The Cas9 coding sequence was placed under the control of 35S promoter, the *nptII* gene for kanamycin resistance was under pNos promoter, while gRNAs were under pU6-26 promoter.

The final vector, named alpha_nptII_Cas9_gRNA1_gRNA2 (**Supplementary File S2**), was transferred into the *Agrobacterium tumefaciens* EHA105 strain (Hood et al., 1993) by using the freeze/thaw method (Xu and Li, 2008).

### *De novo* shoot organogenesis and genetic transformation

Approximately 1144 leaf explants were excised from 3-week-old *in vitro* cultured shoots of cv. Berkeley and placed on 44 Petri dishes (26 leaf each one), following the protocol described by Marangelli et al. (2022). Briefly, the leaf explants were placed abaxial side up on the adventitious shoots organogenesis medium (SOM), consisting of WPM including vitamins, sucrose 3%, and 11.4 µM of zeatin riboside (ZR), (pH 5.6, gelled with 0.7% w/v agar - B&V, Italy), supplemented with 100 µM Acetosyringone (AS).

For genetic transformation experiments, *A. tumefaciens* EHA105-alpha1_NptII_Cas9_gRNA1_gRNA2 was grown overnight in Luria-Bertani (LB) selection medium supplemented with 50 mg L^-1^ kanamycin and 50 mg L^-1^ rifampicin at 28°C with shaking (100 rpm). Then, an aliquot (0.2-0.5 ml) of actively growing bacterial suspension was resuspended into 10 ml of LB (with kanamycin and rifampicin), under the same conditions, until an OD_600_ = 0.6 was reached. In a next step, centrifugation was used to recover the bacteria (4,000 rpm for 15 min) and the pellet was then resuspended in 10 ml of hormone-free SOM supplemented with 100 µM AS.

After 24 h in dark conditions at 28°C, a few drops of the *Agrobacterium* suspension were poured over the leaves and, after 10-15 minutes, the excess fluid was removed by absorption with sterile paper discs. The explants were then co-cultured with the engineered *Agrobacterium* EHA105 on the same medium (SOM + 100 µM AS) for 48, 72 and 96 hours, in dark conditions at 24°C.

After co-cultivation, half of the explants were placed in the same medium above described supplemented with 150 mg L^-1^ Cefotaxime (Cfx) alone, or in combination with 50 mg L^-1^ kanamycin (Kan). The same media have been used to induce adventitious shoot organogenesis on untreated leaf explants (as control). Since for the explants cultured on the medium enriched with kanamycin, no organogenesis occurs, all the explants have been transferred to the SOM medium containing 200 mg L^-1^ cefotaxime alone. The plates with the organogenetic tissues were checked daily for possible contaminations due to *Agrobacterium* strain used or other contaminants, and to check for the appearance of any mutant phenotypes. Throughout the experiment, regenerated shoots showing mutant phenotypes were isolated and after six weeks from the start of the experiments, all the regenerated shoots have been transferred to a PM supplemented with 200 mg L^-1^ Cfx and 50 mg L^-1^ Kan. Some of the mutants have been selected for molecular analysis.

### Scanning of electron microscopy

To better understand when the shoot organogenesis occurs, early observations have been done by SEM analyses. Samples were fixed overnight at 4°C with 2.5% (v/v) glutaraldehyde + 2% (v/v) paraformaldehyde in 0.1 M cacodylate buffer, pH 7.2. After 3x20 min washings at 4°C in the same buffer, samples were post-fixed with 2% (v/v) osmium tetroxide in 0.1M cacodylate buffer, pH 7.2 for 2 h at 4°C. Specimens were washed in the same buffer (3 changes for 15 min each at 4°C), and then dehydrated in a graded ethanol series. Samples were dried by the critical point method using CO_2_ in a Balzers Union CPD 020. Then samples were attached to aluminum stubs using carbon tape and sputter-coated with gold in a Balzers MED 010 unit. The observations were made by a JEOL JSM 6010LA electron microscope.

### Molecular analysis of kanamycin-resistant mutants

Genomic DNAs were extracted from 0.1 g of plant tissue obtained through transformation with nptII_*Cas9*_*pds*_gRNA1_gRNA2 vector using EZNA^®^ Plant DNA kit (Omega Bio-tek, Norcross, GA, USA). The kanamycin-resistant shoots include not only the albino phenotype but, in general, all the shoots showing an atypical phenotype and/or resistance to the presence of kanamycin in the growing medium.

Mutation frequencies at the target sites were evaluated through PCR amplification using primers designed on gRNAs flanking regions (**Table 2**). DNA was amplified using KAPA HIFI Taq (KapaBiosystems, Roche, Basel, Switzerland) and the following PCR program: 95°C/3 min, followed by 30 cycles of 98°C/20 sec, 60°C/20 sec, 72°C/45 sec and 72°C/3 min. The PCR products were purified using DNA/RNA Clean Up E.Z.N.A.^®^ kit (Omega Bio-tek, Norcross, GA, USA). Samples were sequenced using the Sanger method and the chromatograms obtained were analyzed using the TIDE online software (https://tide.deskgen.com, accessed on 31^st^ July 2022).

### Statistical analysis

The data was subjected to analysis of variance (ANOVA) performed using the software SPSS version 20.0. Percentage data were transformed as the arcsin of the square root of p/100 before analysis of variance. Data were presented as means of three replications at least. Significance was determined using Duncan’s test (*p* < 0.05).

## Results and discussion

### Gene structure and phylogenetic analysis

**Figure 1:**
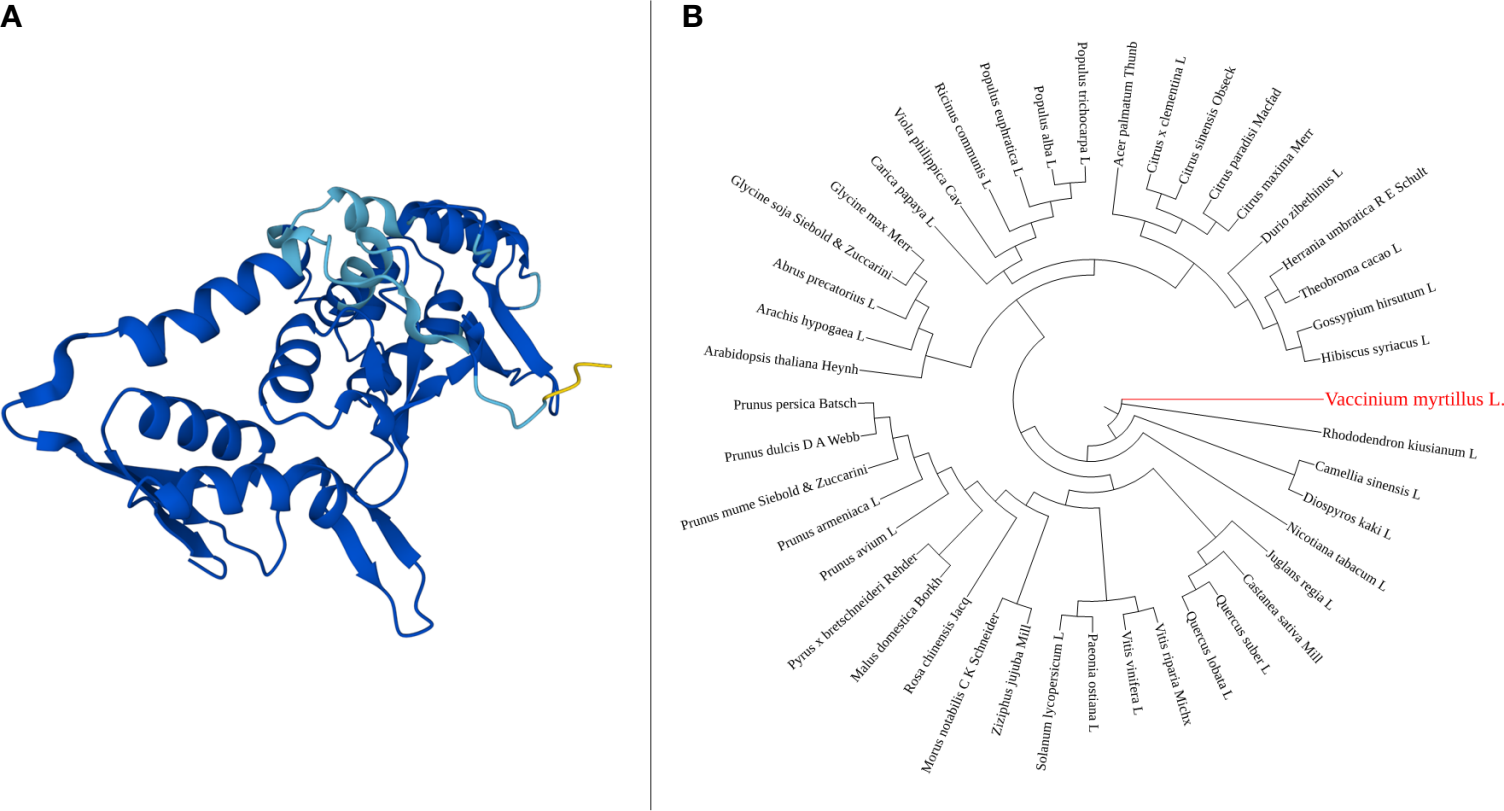
PDS amino_oxidase domain 3D structure, generated using AlphaFold online tool **(A)**. Phylogenetic tree of *pds* gene, constructed using NGPhylogeny **(B)**.

The *Vmpds* coding sequence is 1,737 bp long, and the protein size is 578 amino acids. Within the protein sequence a single amino_oxidase domain is present (**Figure 1A**). The amino_oxidase domain starts at the amino acid position 49 and ends at position 274 and has the molecular function of catalyzing oxidation-reduction (redox) reactions. Available full-length NCBI *pds* coding sequence orthologues (**Supplementary File 1**) were used for phylogenetic tree construction, and the resulting unrooted maximum-likelihood tree shown in **Figure 1B** suggests the phylogenetic relationship of gene sequence between *V. myrtillus* L. and *Rhododendron kiusianum* L. (94% bootstrap value). Both are species belonging to the Ericacee family, and they represent species-rich groups within the Ericacee (Rose et al., 2018). Affinity also emerged with *Camellia sinensis* L. and *Diospyros kaki* L. which belong to different families but from Ericales order.

### CRISPR/Cas9-mediated transformation of highbush blueberry

**Figure 2:**
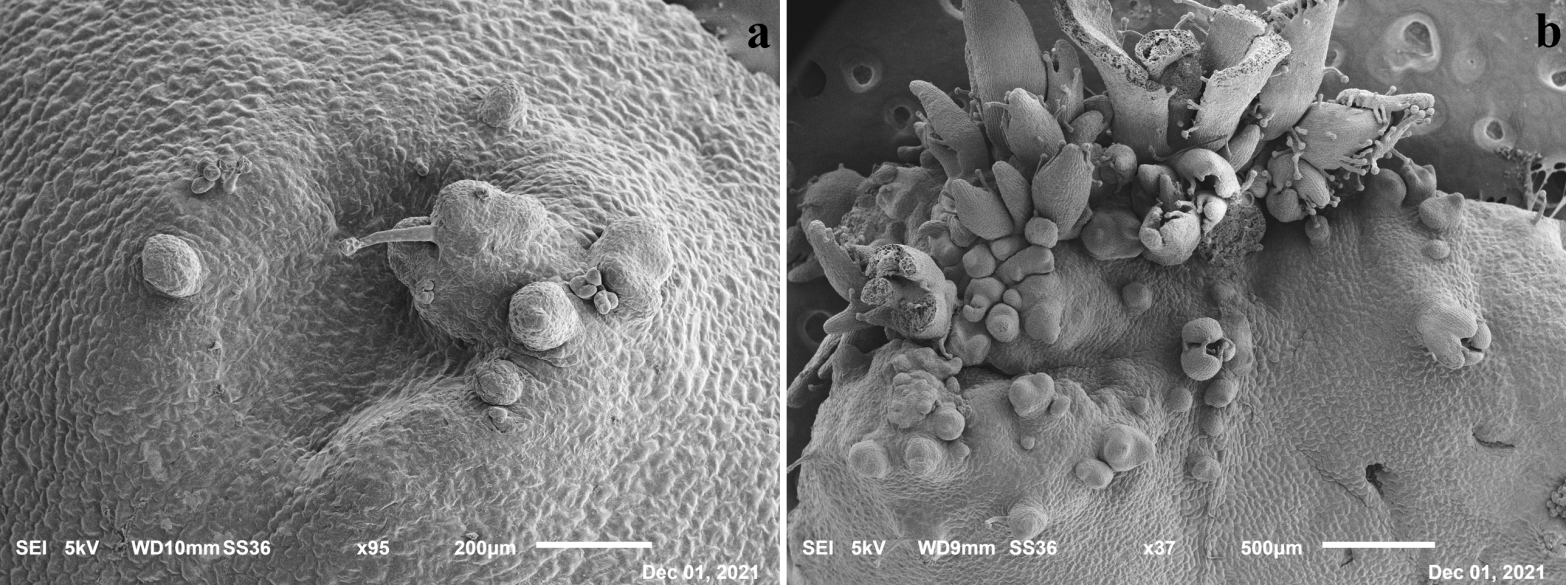
Scanning Electron Microscope (SEM) images of leaf tissues after 5 days in SOM medium with evidence of single **(A)** and grouped primordia **(B)**.

The presence of *de novo* shoot organogenesis was simultaneously observed just 5–6 days after the begin of the experiments on the control, in which single (**Figure 2A**) or grouped bud primordia (**Figure 2B**) were observed, confirming that the induction and differentiation phases in this species take place early as recently highlighted (Marangelli et al., 2022).

Different from other species, shoot organogenesis did not occur frequently from the wounds but on the whole leaf surface, as also reported in Marangelli et al. (2022). These results were also observed by Liu et al. (2010), who described two different patterns during the *de novo* organogenesis processes of various cultivars of southern highbush blueberry: pattern I, where regenerants emerged mainly from the portion closer to the cutting area, and pattern II, where regeneration occurred directly from the entire leaf blade.

As shown in **Table 3**, a total of 936 explants has been used for genetic transformation, and 208 explants used as control (no co-culture with *A. tumefaciens* strain). Furthermore, in a preliminary experiment we also tested the effect of cefotaxime on the regeneration potentiality of highbush blueberry leaves cv. Berkeley (data not shown). The results of the experiment display that the presence of Cfx at 150 mg L^-1^ does not affect the regeneration process. As previously observed some antibiotics can affect morphogenesis, while kanamycin exerts a negative effect in some species, including in highbush blueberry, a positive effect has been highlighted when β-lactams such as cefotaxime are added to the regeneration media (Bosela, 2009; Silvestri et al., 2016).

**Table 3:** Summary of Berkeley transformation (data considered only the explants regenerated on the medium containing only cefotaxime, because in the regeneration medium containing kanamycin, no regenerations occur).

Explant origin	Co-culture duration (h)	Number of inoculated leaf explants	No. of leaf producing shoots (%)	No. of kan-resistant shoots analyzed	No. of mutated shoots
Glass Jars	0 (control)	52	59.1 ± 2.9 a	–	
	48	78	29.3 ± 3.8 b	11	5
	72	78	28.1 ± 1.1 b	10	6
	96	78	26.9 ± 2.3 b	10	3
Average			35.8 ± 15.5 B		
PVC Jars	0 (control)	52	73.0 ± 0.8 a	–	
	48	78	37.3 ± 0.9 b	10	3
	72	78	39.4 ± 1.9 b	10	4
	96	78	38.6 ± 1.4 b	9	3
Average			47.1 ± 17.3 A		

Data are represented as mean ± standard deviations. Upper case letters indicate significant differences among explant origin, and lower case letters indicate different between co-culture duration.

After about ten days, when the first regenerations were observed in explants cultured on media enriched with only Cfx, no regeneration events were detected on the explants cultured on media also enriched with kanamycin. In general, a significant difference was observed in the regeneration ability of the explants derived from proliferation in glass jars or in PVC-jars, both in the controls and in the *Agrobacterium*-inoculated leaves. In fact, the average number of leaf-forming shoots was higher for the leaves harvested in PVC-Jars than the ones derived from glass jars (47.1 ± 17.3 vs 35.8 ± 15.5 respectively) (**Table 3**).

**Figure 3:**
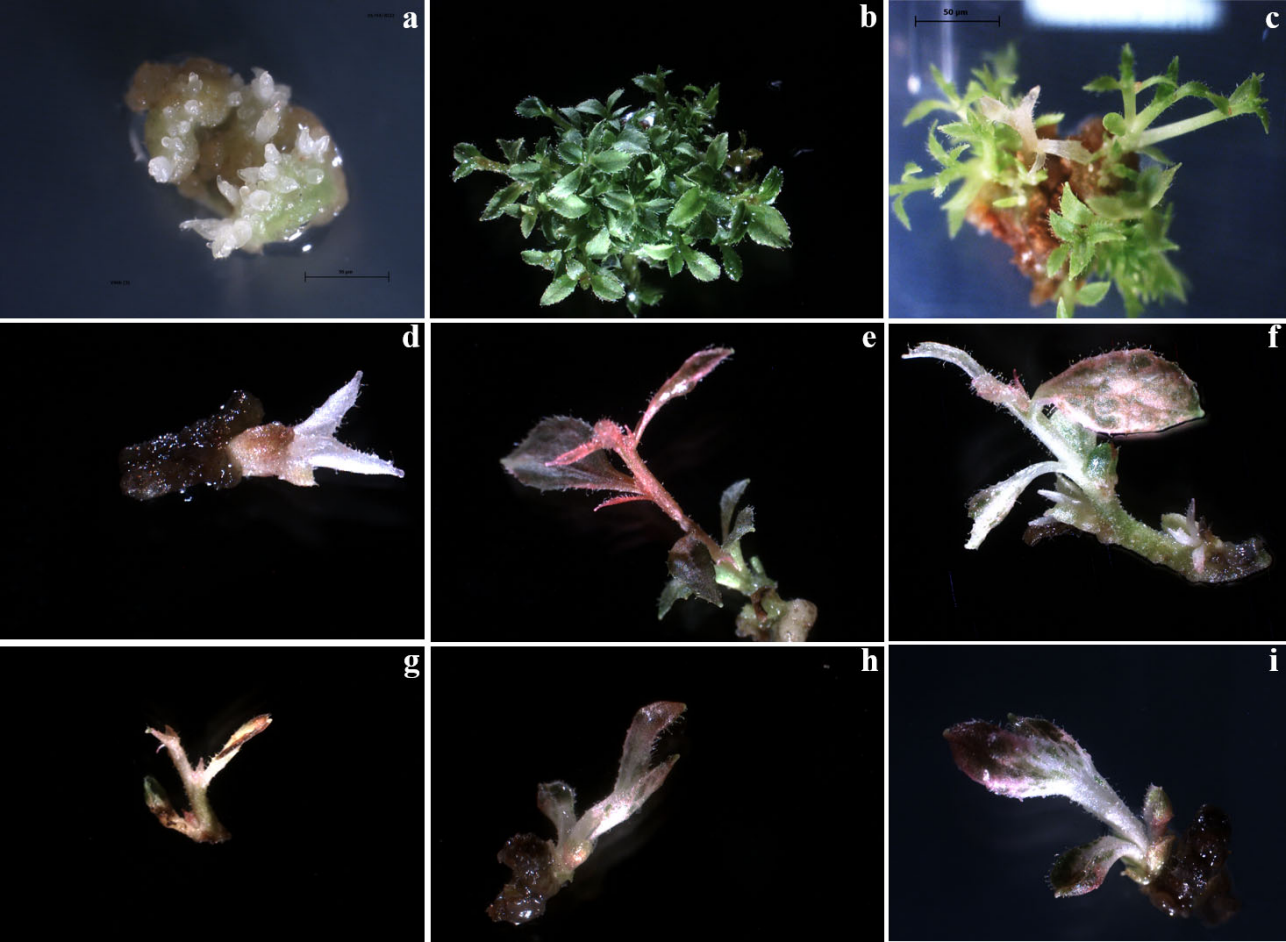
Editing of highbush blueberry cv Berkeley by knockout of pds gene with CRISPR/Cas9. **(A)** regenerating tissues produces numerous adventitious buds, **(B)** that rapidly evolve in tiny shoots (up to 30 shoots per explant). **(C-D)** regenerated shoots showed the expected albino phenotype. **(E)** shoot characterized by a red pigmentation and, **(F, G, H, I)** regenerated shoots showing chimeric phenotypes.

As confirmed by statistical analysis, co-culture with the bacteria resulted in a significant reduction in the regeneration efficiency of leaf explants, irrespective of both the origin (glass- or PVC jars) and the duration of co-culture (48, 72 and 96 hours). On the regeneration media containing only Cfx, the regenerating tissues produce numerous adventitious buds just after a couple of weeks (**Figure 3A**) which develop into tiny shoots (up to 30 shoots per explant) (**Figure 3B**). When they were transferred to the PM supplemented with 200 mg L^-1^ Cfx and 50 mg L^-1^ Kan, most of the explants continue to grow but, after two weeks, only the explants showing altered phenotypes were able to grow in kanamycin-enriched medium, while most of the shoots stop the growth and, in some cases, they rapidly turn brown. A broad phenotypic variation was observed among the regenerated shoots. In few cases, regenerated shoots showed the expected albino phenotype (**Figures 3C, D**), some shoots were characterized by a uniform red pigmentation (**Figure 3E**), and in many cases, instead, shoots with chimeric phenotype were observed, presenting green, white, and red colorations simultaneously (**Figures 3F-I**), emphasizing the difficulty to obtain fully edited shoots. It appears that explants exhibiting an albino phenotype showed stunted growth also if cultivated in *in vitro* conditions on an artificial medium. This characteristic could be ascribed to the fact that PDS represents not only an essential plant carotenoid biosynthetic enzyme and a prominent target of certain inhibitors, such as norflurazon, acting as bleaching herbicides (Koschmieder et al., 2017), but also an endogenous plant growth regulator, due to its role in gibberellin biosynthesis (Qin et al., 2007). Indeed, it has been demonstrated how PDS disruption could lead to a GA deficiency. Since the processes of adventitious organogenesis are particularly sensitive to hormonal balances, this aspect should be further investigated.

### Mutant screening

To confirm the editing event, kanamycin resistant selected samples were sequenced through the Sanger sequencing and the resulting chromatograms were analyzed using the TIDE online software. The editing efficiencies were different among the two gRNAs (**Table 4**) ranging between 2.1 and 9.6% for gRNA1 and 3.0 and 23.8 for gRNA2. It reinforces the necessity to use different guides simultaneously to ensure an efficient gene knockout (Pavese et al., 2021).

**Table 4:** Genotyping of targeted gene mutations induced by CRISPR/Cas9 in the transformed samples: editing efficiency, goodness-of-fit (R^2^), mutations and zygosity (HE – heterozygosity or CH – chimeric).

	gRNA1				gRNA2			
Sample	Efficiency (%)	R^2^	mutations	zygosity	Efficiency (%)	R^2^	mutations	zygosity
**1**	-	-	-	**-**	3.9	0.98	-1	**HE**
**2**	-	-	-	**-**	3.4	0.98	-1	**HE**
**3**	9.6	0.93	+1	**HE**	4.7	0.99	-1	**HE**
**4**	2.1	0.99	-1	**HE**	3.4	0.99	-1	**HE**
**5**	-	-	-	**-**	3.0	0.99	-8	**HE**
**6**	7.2	0.98	-1	**HE**	-	-	-	**-**
**7**	7.3	0.99	-1	**HE**	10.0	0.98	-1; +1	**CH**
**8**	7.5	0.99	-1	**HE**	-	-	-	**-**
**9**	-	-	-	**-**	9.4	0.97	-1; +1	**CH**
**10**	7.4	0.98	-1	**HE**	-	-	-	**-**
**11**	-	-	-	**-**	7.7	0.98	-1	**HE**
**12**	4.5	0.99	-1	**HE**	-	-	-	**-**
**13**	5.9	0.99	-1	**HE**	4.7	0.99	-1	**HE**
**14**	7.3	0.99	-1	**HE**	-	-	-	**-**
**15**	4.2	0.99	-1	**HE**	4.0	0.99	-1; +1	**CH**
**16**	-	-	-	**-**	13.3	0.96	-1; +1	**CH**
**17**	8.0	0.99	-1	**HE**	7.6	0.99	-1; +1	**CH**
**18**	-	-	-	**-**	7.9	0.97	+1	**HE**
**19**	-	-	-	**-**	4.3	0.97	-1	**HE**
**20**	8.2	0.98	+1	**HE**	8.2	0.95	-1	**HE**
**21**	7.1	0.97	-1	**HE**	4.0	0.97	+1	**HE**
**23**	7.2	0.98	-1	**HE**	5.7	0.97	-1; +1	**CH**
**24**	7.0	0.96	-1	**HE**	23.8	0.97	+1	**HE**

As reported in **Table 4**, of the 24 lines analyzed, 16 lines were edited for gRNA1 (66.6%) while 19 for gRNA2 (79%). While for gRNA1 only heterozygous mutants were observed, for gRNA2 6 out of 19 edited lines (31%) resulted chimeric. Our results are in agreement with the results reported by Pavese et al. (2021) for European chestnut, where they found almost all heterozygous lines, contrarily to other woody crops, in which most of the mutants were homologous and biallelic (Nishitani et al., 2016; Charrier et al., 2019; Dai et al., 2021). Chimeric genotypes represent one of the major concerns in genome editing, especially for heterozygous species such as perennial fruit crops characterized by high degree of heterozygosity, the length of their juvenile phase, the long generation times and auto-incompatibility systems (Ding et al., 2020). Anyway, this problem could be minimized through the second round of adventitious shoot organogenesis, as recently reported for highbush blueberry by Han et al. (2022).

The most common mutations observed in the edited lines were single insertions or deletions (+/-1 bp) and, only in one case (line 5) a -8 bp deletion was observed. Previous observations (Bortesi et al., 2016) showed that, in most cases, one bp insertions and deletions are the predominant ones, especially interesting T and A (Bortesi et al., 2016). Anyway, the type and size of mutations could vary considerably, probably due to different factors such as target sequences, vectors, and plant species (Jacobs et al., 2015; Xu et al., 2015; Pavese et al., 2021).

The editing efficiency we reported (up to 23.8%) is in line with the only two manuscripts so far published by other authors on highbush blueberry. The first work described the knockout of the *Centroradialis* (*Cen*) gene, selected because it is present in a single-copy gene in the polyploid genome of the highbush blueberry plant. The authors demonstrated for the first time the applicability of this technique to highbush blueberry, obtaining different edited lines with an average mutated allele ratio of 19-22%, on the basis of the selected gRNA. Nevertheless, the editing efficiency of fruit crops is very variable: up to 90% in apple (Osakabe et al., 2018; Charrier et al., 2019), less than 10% in callus of kiwifruit (Wang et al., 2018), 51% in *Populus* spp. (Fan et al., 2017), 31% in grape (Wang et al., 2018), 35% in orange (Peng et al., 2017) and higher than 90% in *Castanea sativa* Mill (Pavese et al., 2021).

**Figure 4:**
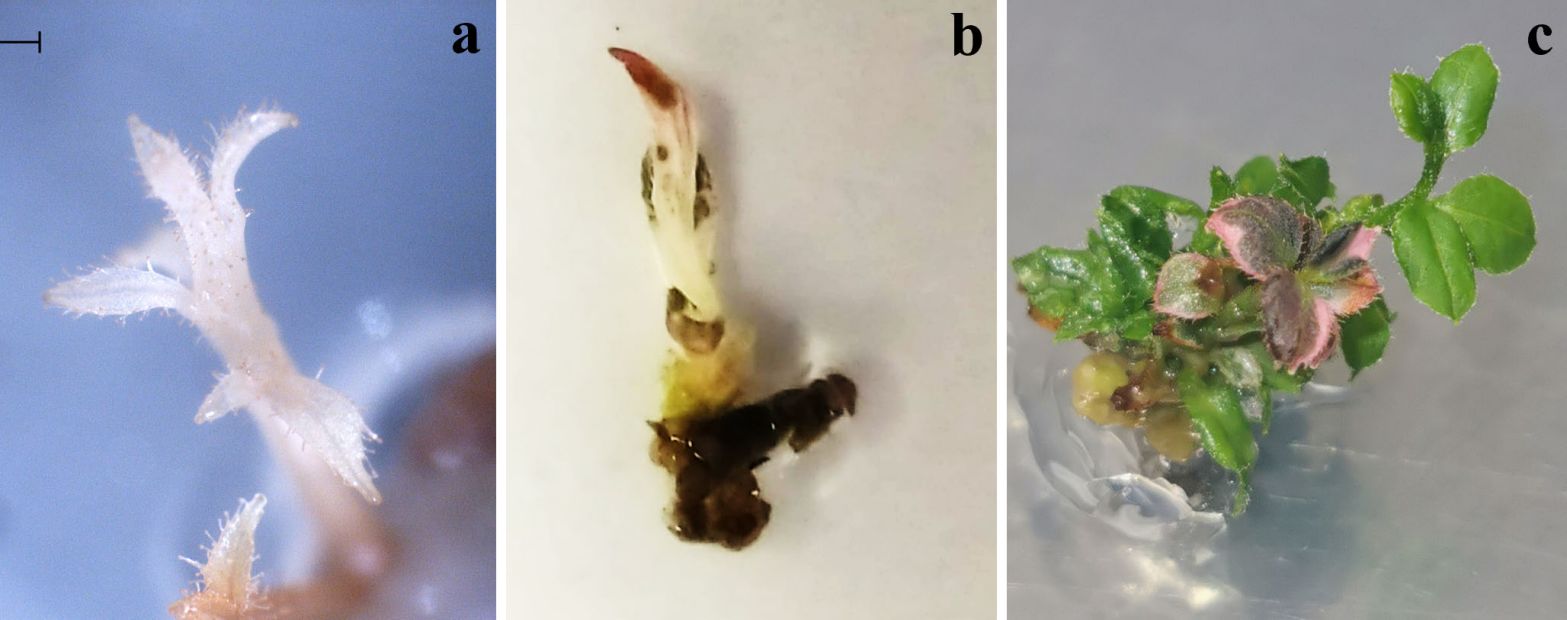
Edited phenotypes of highbush blueberry cv Berkeley. A developed albino phenotype **(A)**, albino/chimeric phenotype showing island of green tissues **(B)** and an edited chimeric shoot showing red color due to the presence of anthocyanins **(C)**.

In our experiments, a complete albino phenotype was observed even with low editing efficiency (23.8%) **(** **Figure 4A**), as well chimeric mutants showing green islands (**Figure 4B**) or anthocyanins (**Figure 4C**), that often appears with the albinism, as also observed in other species (Henderson et al., 2020). Anyway, the CRISPR/Cas9 in polyploids is more challenging, due to the presence of multiple homoeologous copies (or alleles) of each gene (Schaart et al., 2021), and low editing efficiency could be ascribed to different reasons. In fact, Wilson et al. (2019) demonstrated that by targeting *pds* gene in octoploid strawberry, some albino shoots were obtained also when wild-type *pds* alleles were still present and, in the same way, also they observed pale-green shoots containing the 11% of the wild-type alleles, assuming that probably just one allele is responsible for enzyme production and the others are non-functional alleles. Martin-Pizarro et al. (2019), by targeting *FaTM6* gene to study the function of the MADS box transcription factor during flower development of *Fragaria x ananassa* obtained the expected phenotype with modified anthers even though none of the edited lines showed mutations in all present alleles. Similarly, Gao et al. (2020) targeted the *Reduced Anthocyanins in Petioles* (*RAP*) gene in octoploid strawberry, obtaining changes in fruit colour without the occurrence of the editing in all the alleles, confirming that the loss of functional alleles is a common phenomenon in allopolyploids (Wendel et al., 2018).

Here we found a very effective adventitious shoot regeneration protocol for highbush blueberry, leading to the regeneration of up to 30 adventitious shoots from each leaf tissue. These results confirmed that zeatin and zeatin riboside are very useful to obtain high-efficient protocols for adventitious shoot organogenesis in some blueberry varieties (Rowland and Ogden, 1992; Cao and Hammerschlag, 2000; Marangelli et al., 2022).

Regarding the experiments of genetic transformation and genome editing, our results indicate that the knockout of *pds* gene by using CRISPR/Cas9 technology is efficient in highbush blueberry, opening the opportunity to the functional knockout of interesting candidate genes.

## Conclusions

The CRISPR/Cas technology is representing the most powerful tool in the era of New Plant Breeding Techniques (NPBTs), able to speed up the breeding and genetic improvement of plants, especially nowadays, as the agriculture is facing the climate change accompanied by an increase of potential climate impacts, with the urgency to address and guide agricultural adaptation.

Our results represent an update of the genome editing technology applied to highbush blueberry, and the first example of the *pds* knockout gene in this species. The protocol is efficient and reliable, but further improvements are needed to make this technique a ready-to-use tool in the genetic improvement of this high-value species, by studying other important factors such as pH of the medium, culture conditions, light, temperature and considering the different response of the genotypes.

Moreover, chimerism is not necessarily a disadvantage, but it can become particularly attractive in species for which efficient protocols of adventitious organogenesis exist, where a second round of regeneration could eliminate the chimerism rate and increase the editing efficiency, as described by Han et al. (2022).

This protocol can be used for the genetic improvement of some important traits for the highbush blueberry, such as low chilling requirements, superior fruit quality (Ferrao et al., 2021), increased shelf-life and tolerance/resistance to the most important pests and diseases.

However, the breakthrough in the application of genome editing technology lies precisely in the possibility of producing transgene-free mutants. To avoid the stable integration of recombinant DNA, the CRISPR/Cas9 RNP approach combined with protoplasts can represent an innovative way to obtain transgene-free plants leading to potential better acceptance by consumers and opening at legislative level.

## Data availability statement

The original contributions presented in the study are included in the article/**Supplementary Material**. Further inquiries can be directed to the corresponding authors.

## Author contributions

CS, VP and AM: conceptualization. GV, VP and CS: data curation and writing—original draft preparation. GV, VP and CS: investigation. AM, CS and VC: supervision and writing—review and editing preparation. All authors contributed to the article and approved the submitted version.

## Supplementary material

Supplementary File 1
*#pds* coding sequences used for phylogenetic tree construction using NGPhylogeny (https://ngphylogeny.fr/, accessed on 31^st^ July 2022).

Supplementary File 2 Final vector containing gRNAs, hCas9 and nptII, assembled using GoldenBraid technology.
