# Supplementary figures and images for: Knockout of phytoene desaturase gene using CRISPR/Cas9 in highbush blueberry
Source: Front Plant Sci. 2022 Dec 15;13:1074541. doi: 10.3389/fpls.2022.1074541 (PMC9800005; doi:10.3389/fpls.2022.1074541)

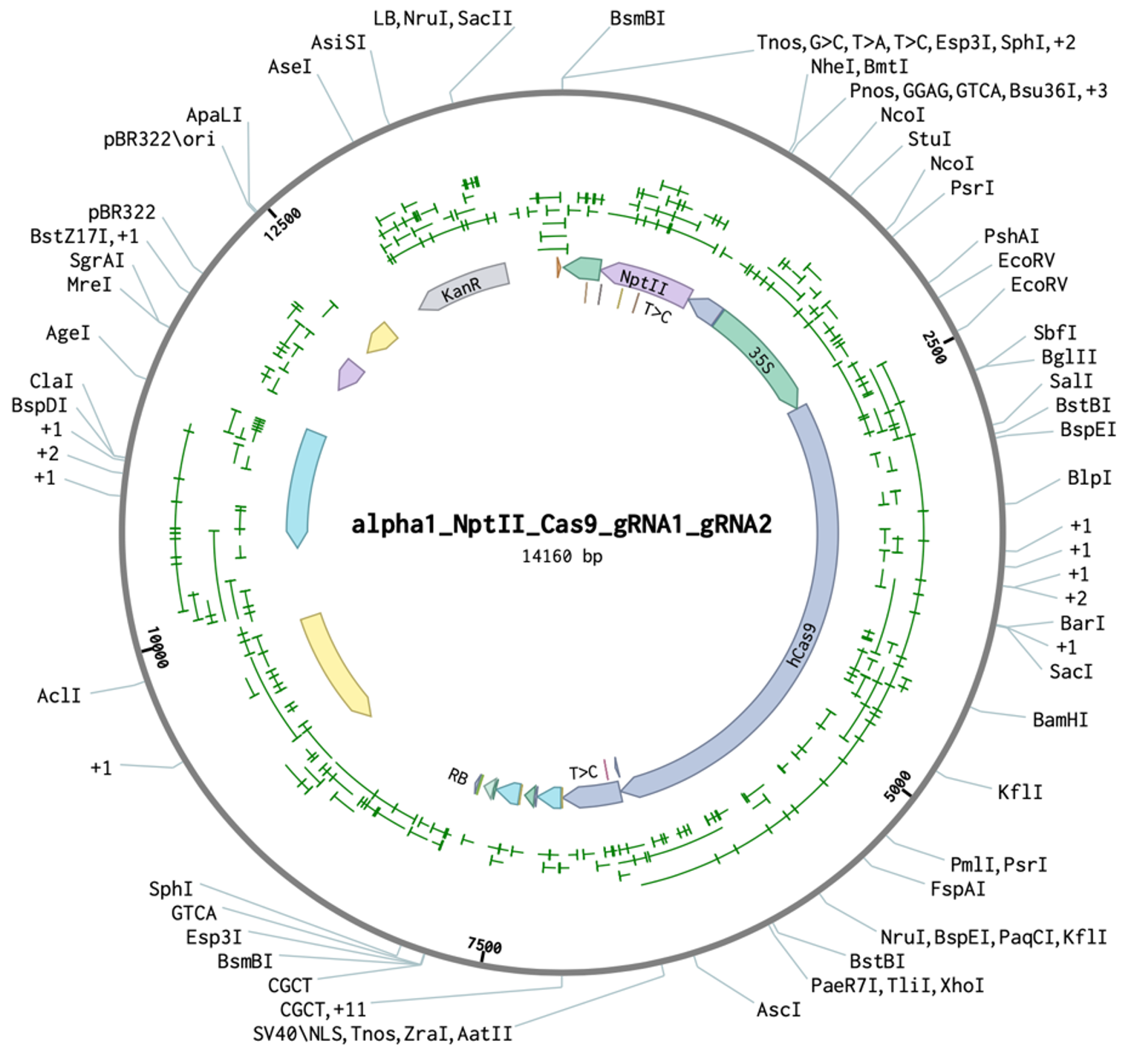

Supplement: Supplementary File 1 — #pds coding sequences used for phylogenetic tree construction using NGPhylogeny (https://ngphylogeny.fr/, accessed on 31st July 2022). [file Image_1.tif]
