# Supplementary material for: Knockout of phytoene desaturase gene using CRISPR/Cas9 in highbush blueberry
Source: Front Plant Sci. 2022 Dec 15;13:1074541. doi: 10.3389/fpls.2022.1074541 (PMC9800005; doi:10.3389/fpls.2022.1074541)
Supplement: Supplementary file 2 [file Table_1.docx]

**Supplementary file 1|** *#pds* coding sequences used for phylogenetic tree construction using NGPhylogeny (<https://ngphylogeny.fr/>, accessed on 31^st^ July, 2022).

***> Quercus suber***

ATGACCATTGGTGGGTTTGTTTCGGCTGCAAACTTGAGCTGCCAAAGTACTTTGACAG AAATCAAACTCTGGGATGTGGGTTTCTTAATAATTCAGTGAAAACCAATGCATTAGCA TTGGAGGTTGTGAATCCATGGGTCATAGTTTGAGAATTCCACATACAAAGGCTATTA ATTGAGGCCGAGGAAGGGTGTCTCTCCTTTGCAGGTAGTATGTATGGACTTCCCAAG CCAGAGCTTGAGAATACTGTTAATTTCTTAGAGGCTGCTTATTTGTCTTCTTCCTTTCG GCATCTGCTCGTCCATCTAAACCCCTAACAGTTGTAATTGCTGGTGCAGGTTTGGCTG TTTGTCTACTGCAAAGTATTTGGCAGATGCTGGTCACAAACCTATACTATTGGAGTCA GAGATGTACTAGGAGGAAAGGTGGCTGCATGGAAAGATGACGATGGAGACTGGTAT AGACTGGATTACATATATTCTTTGGGGCTTACCCAAATGTGCAGAATCTGTTCGGAGA CTTGGTATTGATGATCGGTTGCAATGGAAGGAACATTCTATGATTTTTGCAATGCCAAATAAGCCGGGAGAGTTCAGCCGATTTGATTTTCCTGAAGTTCTTCCTGCACCATTAAATGGAATATGGGCTATCTTGAAGAACAATGAGATGCTGACTTGGCCAGATAAAGTCAAGTTTGCGATTGGACTCTTGCCGGCAATGCTTGGTGGACAGGCTTATGTTGAAGCTCAAGATGGTTTAACTGTTAAAGAGTGGATGAGAAAGCAGGGAGTACCTGATCGTGTAACTGATGAGGTATTTGTAGCCATGTCAAAGGCGCTAAACTTCATTAACCCCGATGAACTTTCAATGCAATGCATATTGATTGCTTTGAATAGGTTTCTTCAGGAGAAGAATGGTTCCAAGATGGCTTTCTTGGATGGTAATCCCCCAGAGAGACTCTGTATGCCAATTGTTGATCATATTCAATCACTAGGTGGTGAAGTAAGACTAAATTCGAGAATACAAAAAATCGAGCTAAATAATGATGGAACAGTGAAAAGCTTTTTACTGAATAATGGGAACATGATTGAAGGAGATGCTTATGTATTTGCTACTCCAGTTGATATCCTGAAGCTTCTTTTGCCGGAAAACTGGAAAGAGATTCCATATTTCCAGAGATTAAAGAAATTAGTTGGAGTTCCAGTTATTAATGTCCACATATGGTTTGACAGAAAACTGAAGAACACCTATGATCACCTACTGTTTAGCAGAAGTCCACTTCTCAGTGTGTATGCTGACATGTCAGTAACATGTAAGGAATATTACAACCCAAACCAATCTATGCTGGAGTTGGTTTTTGCGCCTGCAGAAGAATGGATTTCACGCAGTGACTCAGACATTATTGACGCTACAATGAATGAACTTGCAAGACTCTTTCCTGATGAAATTTCCACGGATCAAAGCAAAGCAAAGATTGTGAAGTACCATGTTGTTAAAACACCAAGGTCTGTTTACAAAACTGTCCCAGACTGTGAACCTTGCCGTCCCTTACAAAGATCTCCTATTGAGGGGTTTTACTTAGCTGGTGACTACACAAAACAAAAATATTTGGCTTCAATGGAAGGTGCTGTTCTGTCAGGAAAGCTTTGTGCTCAGGCTATTGTACAGGATTATGAGTTGCTTATAGCTCGGGGGCAAACAAGGTTGGCTCAAGCAAGTGTTTATTGA

***>Quercus lobata***

ATGACCATTGGTGGGTTTGTTTCGGCTGCAAACTTGAGCTGCCAAAGTACTTTGACAGGAAATCAAACTCTGAGATGTGGGTTTCTTAATAATTCAGTGAAAACCAATGCATTAGCATTTGGAGGTTGTGAATCCATGGGCCATAGTTTGAGAATTCCACATACAAAGGCTATTAGATTGAGGCCGAGGAAGGGTGTCTCTCCTTTGCAGGTAGTATGTATGGACTTTCCAAGACCAGAGCTTGAGAATACTGTTAATTTCTTAGAGGCTGCTTATTTGTCTTCTTCCTTTCGTGCATCTGCTCGTCCATCTAAACCCCTAACAGTTGTAATTGCTGGTGCAGGTTTGGCTGGTTTGTCTACTGCAAAGTATTTGGCAGATGCTGGTCACAAACCTATACTATTGGAGTCAAGAGATGTACTAGGAGGAAAGGTGGCTGCATGGAAAGATAACGATGGAGACTGGTATGAGACTGGATTACATATATTCTTTGGGGCTTACCCAAATGTGCAGAATCTGTTCGGAGAACTTGGTATTGATGATCGGTTGCAATGGAAGGAGCATTCTATGATTTTTGCAATGCCAAATAAGCCGGGAGAGTTCAGCCGATTTGATTTTCCTGAAGTTCTTCCTGCACCATTAAATGGAATATGGGCTATCTTGAAGAACAATGAGATGCTGACTTGGCCAGATAAAGTCAAGTTTGCGATTGGACTCTTGCCAGCAATGCTTGGTGGACAGGCTTATGTTGAAGCTCAAGATGGTTTAACTGTTAAAGAGTGGATGAGAAAGCAGGGAGTACCTGATCGTGTAACTGATGAGGTATTTGTAGCCATGTCAAAGGCGCTAAACTTCATTAACCCCGATGAACTTTCAATGCAATGCATATTGATTGCTTTGAATAGGTTTCTTCAGGAGAAGAATGGTTCCAAGATGGCTTTCTTGGATGGTAATCCCCCAGAGAGACTCTGTATGCCAATTGTTGATCATATTCAATCACTAGGTGGTGAAGTAAGACTAAATTCAAGAATACAAAAAATCGAGCTAAATAATGATGGAACAGTGAAAAGCTTTTTACTGAATAATGGGAACATGATTGAAGGAGATGCTTATGTATTTGCTACTCCGGTTGATATCCTGAAGCTTCTTTTGCCGGAAAACTGGAAAGAGATTCCATATTTCCAGAGATTAAAGAAATTAGTTGGAGTTCCAGTTATTAATGTCCACATATGGTTTGACAGAAAACTGAAGAACACCTATGATCACCTACTGTTTAGCAGAAGTCCACTTCTCAGTGTGTATGCTGACATGTCAGTAACATGTAAGGAATATTACAACCCAAACCAATCTATGCTGGAGTTGGTTTTTGCGCCTGCAGAAGAATGGATTTCACGCAGTGACTCAGACATTATTGACGCTACAATGAATGAACTTGCAAGACTCTTTCCCGATGAAATTTCCACGGATCAAAGCAAAGCAAAGATTGTGAAGTACCATGTTGTTAAAACACCAAGGTCTGTTTACAAAACTGTCCCAGACTGTGAACCTTGCCGTCCCTTACAAAGATCTCCTATTGAGGGGTTTTACTTAGCTGGTGACTACACAAAACAAAAATATTTGGCTTCAATGGAAGGTGCTGTTCTGTCAGGAAAGCTTTGTGCTCAGGCTATTGTACAGGATTATGAGTTGCTTATAGCTCGGGGGCAAACAAGGTTGGCTCAAGCAAGTGTTTATTGA

***>Juglans regia***

ATGGCCCTTTGCGGGTGTGTTTCGGCCGCGAACTTGAGCTGGCAGAATACTGTTATAGAAAATCACAGTTTGAAAACTGCCCCAAGATGTGGGTTTCTTAAAGTTTCAGAGAAAACCAATGCATTAGCATTTGGAGGTAGTGAATCCATGGGTCATAGTTTGAAAATTAATTATCCACATGCTGTGGGATTAAGGCCAAGGAAGGGTGTCTACCCTTTGCAGGTAGTATGCATGGACTTCCCAAGACCAGAACTTGATAATACTGTTAATTTCTTAGAAGCCGCATACCTCTCTTCCTCCTTTCGTACTTCTCCCCGCCCAACTAAACCTTTAACAGTGGTAATTGCTGGTGCAGGTTTGGCTGGTTTGTCAACCGCAAAGTATTTGGCAGATGCTGGTCATAAACCTGTATTATTGGAAGCAAGAGATGTTCTTGGAGGAAAGGTGGCTGCATGGAAAGATGACGATGGAGACTGGTATGAGACAGGATTACATATATTCTTTGGGGCTTACCCAAATGTGCAGAACATATTTGGAGAACTTGGTATTAATGATCGGTTGCAGTGGAAGGAGCACTCTATGATATTTGCAATGCCAAAAAAGCCTGGAGAATTCAGCCGATTTGATTTTCCTGAGGTTCTTCCTGCACCATTAAATGGAATATTTGCTATTTTGAGGAACAATGAAATGTTGAGTTGGCCAGAGAAAGTCACGTTTGCCATTGGACTTTTACCTGCGATGCTTGGTGGACAGTCCTATGTTGAAGCTCAAGATGGTTTAACTGTTGAACAGTGGATGAGAAAGCAGGGAATTCCTGATCGTGTAACTGATGAGGTGTTTATAGCAATGTCAAAGGCACTAAACTTCATTAACCCTAAGGAACTTTCAATGCAATGTATATTGATTGCTTTGAACCGGTTTCTTCAGGAAAAGCATGGTTCCAAGATGGCTTTCTTGGATGGTAACCCCCCAGAGAGACTATGTCAGCCAATTGTTGATCATATTCAGTCACTGGGTGGTGAAGTAAGACTAAACTCGCGAATTCAGAATATTGAGCTAAATAGTGATGGAACAGTGAAAAGGTTTTTACTGAATAATGGGAACGTGATTGAAGGGGATGCATATGTATTTGCTACTCCAGTTGACATCCTGAAGCTTCTTTTACCTGAAAACTGGAAAGAGATTCCATATTTCCAGAGATTGGAGAAATTAGTTGGAGTTCCAGTTATTAATGTGCACATATGGTTTGACAGGAAACTGAAGAACACCTATGATCACCTACTATTTAGCAGAAGCCAACTTCTCAGTGTGTATGCTGACATGTCAGTAACATGTAAGGAATATTACAACCCAAACGAATCCATGCTGGAATTAGTTTTTGCGCCTGCAGAAGAATGGATTTCACGCAGTGATGCTGATATTATTGATGCTACAATGAAGGAACTCGCAAAACTCTTTCCAGATGAAATTTCCACAGATCAGAGCAAAGCAAAGATTGTAAAGTACCATGTTGTTAAAACACCAAGGTCCGTTTACAAAAATGTCCCAAATTGTGAACCCTGCCGTCCCTTACAAAGATCTCCTGTTGAGGGCTTCTACTTAGCTGGTGACTACACAAAGCAAAAGTATTTGGCTTCAATGGAAGGTGCTGTTCTGTCAGGAAAGCTTTGTGCTCAAGCTATTGTACAGGATTATGAGTTGCTCGTTGCTCGGGGGCAAAGAAGGTTGGCTCAAGCAAGTCTTTTTTGA

***>Vitis riparia***

ATGACTCAATTCAGATATGTTTCTGCGGTGAACTTGAGCTGCCAAAGTAATATAATAAACTTTCAGAACTCCCAATGTACCTGGAGACATCTTTATATTGATTCAGATCAGACCAATACACTTCTATTTAGAGGTGGTGACTCTATGGGTCTCAAGTTGAGAATTCCAAATAAGCATTCTATTGGAACAAGGCGGAGGAAGGATTTCTGCCCCTTGCAGGTTGTTTGCATGGATTATCCAAGACCAGAACTTGAGAATACTGTGAATTTCTTAGAAGCTGCATACTTATCCTCATCCTTTCATACTTCTCCTCGTCCCAGTAAACCATTAGAGGTTGTAATTGCTGGTGCAGGTTTGGCTGGTTTGTCTACTGCAAAATATTTGGCAGATGCAGGTCACAAGCCTATATTGTTGGAAGCAAGAGATGTTTTAGGTGGAAAGGTGGCTGCATGGAAAGATGAGGATGGAGACTGGTATGAGACAGGCCTACATATATTCTTTGGGGCTTACCCAAATGTGCAGAACCTGTTTGGAGAACTTGGTATTAATGATCGGTTGCAGTGGAAGGAACATTCTATGATATTTGCAATGCCAAGCAAGCCAGGGGAATTCAGCCGATTTGATTTCCCTGAAGTCCTTCCTGCACCCTTAAATGGGATATGGGCCATCTTGAGGAATAATGAAATGCTGACTTGGCCGGAGAAAATCAAGTTTGCTATTGGACTTGTGCCAGCAATGCTCGGAGGACAGGCTTATGTTGAAGCACAGGATGGTTTAACTGTTAAAGACTGGATGAGAAAACAAGGTATTCCTGATCGAGTAACAGATGAGGTTTTCATTGCCATGTCCAAGGCACTGAACTTCATAAATCCGGATGAACTTTCGATGCAGTGTATATTGATTGCTTTGAACCGATTTCTTCAGGAGAAGAATGGCTCCAAGATGGCTTTCTTAGATGGTAATCCTCCAGAGAGACTCTGCATGCCCATTGTTGACCATATTCAGTCACTAGGTGGTCAAGTCCAACTTAATTCACGAATACAAAAGATTGAGCTGAACAAAGATGGAACTGTGAAGAGTTTTGTGCTAAATAATGGGAATGTAATTAAAGGAGATGCTTATGTAATTGCAACTCCAGTTGATATCCTGAAGCTTCTTTTGCCGGGAGACTGGAAAGAGATTCCATACTTCAGGAGATTGGATAAATTAGTTGGAGTTCCAGTGATCAATGTTCATATATGGTTTGACAGGAAACTGAAGAACACATACGATCATCTTCTTTTCAGCAGAAGTCCCCTTCTGAGTGTGTATGCTGACATGTCCGTAACATGTAAGGAATATTACAACCCAAATCAATCTATGCTGGAGTTGGTTTTTGCACCTGCTGAAGAATGGGTCTCACGCAGTGACTCAGAAATCATTGAAGCTACAATGAAGGAACTTGCCAAACTCTTTCCTGATGAAATTTCAGAAGATCAGAGCAAAGCGAAAGTTTTGAAATACCATGTTGTTAAAACACCAAGATCTGTTTACAAAACTGTCCCAAATTGTGAACCTTGCCGTCCCTTACAAAGATCTCCTATAGAGGGCTTTTATTTAGCTGGGGACTACACAAAACAAAAATACTTAGCTTCAATGGAAGGTGCTGTTCTGTCAGGGAAGCTTTGTGCACAGGCTATTGTAAAGGACTATGAATTGCTTGTAGCTCAGGGAGAACAAAAGTTGGCCGAGGTCAGCATTCTCAGTTAA

***>Vitis vinifera***

ATGACTCAATTCAGATATGTTTCTGTGGTGAACTTGAGCTGCCAAAGTAATATAATAAACTTTCAGAACTCCCAATGTACCTGGAGACATCTTTATATTGATTCAGATCAGACCAATACACTTCTATTTGGAGGTGGTGACTCTATGGGTCTCAAGTTGAGAATTCCAAATAAGCATTCTATTGGAACAAGGCGGAGGAAGGATTTCTGCCCCTTGCAGGTTGTTTGCATGGATTATCCAAGACCAGAACTTGAGAATACTGTGAATTTCTTAGAAGCTGCATACTTATCCTCATCCTTTCATACTTCTCCTCGTCCCAGTAAACCATTAGAGGTTGTAATTGCTGGTGCAGGTTTGGCTGGTTTGTCTACTGCAAAATATTTGGCAGATGCAGGTCACAAGCCTATATTGTTGGAAGCAAGAGATGTTTTAGGTGGAAAGGTGGCTGCATGGAAAGATGAGGATGGAGACTGGTATGAGACAGGCCTACATATATTCTTTGGGGCTTACCCAAATGTGCAGAACCTGTTTGGAGAACTTGGTATTAATGATCGGTTGCAGTGGAAGGAACATTCTATGATATTTGCAATGCCAAGCAAGCCAGGGGAATTCAGCCGATTTGATTTCCCTGAAGTCCTTCCTGCACCCTTAAATGGGATATGGGCCATCTTGAGGAATAATGAAATGCTGACTTGGCCGGAGAAAATCAAGTTTGCTATTGGACTTGTGCCAGCAATGCTCGGAGGACAGGCTTATGTTGAAGCACAGGATGGTTTAACTGTTAAAGACTGGATGAGAAAACAAGGTATTCCTGATCGAGTAACAGATGAGGTTTTCATTGCCATGTCCAAGGCACTGAACTTCATAAATCCGGATGAACTTTCGATGCAGTGTATATTGATTGCTTTGAACCGATTTCTTCAGGAGAAGAATGGCTCCAAGATGGCTTTCTTAGATGGTAATCCTCCAGAGAGACTCTGCATGCCCATTGTTGACCATATTCAGTCACTAGGTGGTCAAGTCCAACTTAATTCACGAATACAAAAGATTGAGCTGAACAAAGACGGAACTGTGAAGAGTTTTGTGCTAAATAATGGGAATGTAATTAAAGGAGATGCTTATGTAATTGCAACTCCAGTTGATATCCTGAAGCTTCTTTTGCCGGGAGACTGGAAAGAGATTCCATACTTCAGGAGATTGGATAAATTAGTTGGAGTTCCAGTGATCAATGTTCATATATGGTTTGACAGGAAACTGAAGAACACATACGATCATCTTCTTTTCAGCAGAAGTCCCCTTCTGAGTGTGTATGCTGACATGTCCGTAACATGTAAGGAATATTACAACCCAAATCAATCTATGCTGGAGTTGGTTTTTGCACCTGCTGAAGAATGGGTCTCACGCAGTGACTCAGAAATCATTGAAGCTACAATGAAGGAACTTGCCAAACTCTTTCCTGATGAAATTTCAGAAGATCAGAGCAAAGCGAAAGTTTTGAAATACCATGTTGTTAAAACACCAAGATCTGTTTACAAAACTGTCCCAAATTGTGAACCTTGCCGTTCCTTACAAAGATCTCCTATAGAAGGGCTTTTATTAGCTGGGGACTACACAAAACAAAAATACTTAGCTTCAATGGAAGGTGCTGTTCTGTCAGGGAAGCTTTGTGCACAGGCTATTGTAAAGGACTATGAATTGCTTGTTGCTCAGGGAGAACAAAAGTTGGCCGAGGTCAGCATTCTCAGTTAA

***>Populus alba***

ATGAGTGCATTGAACTTGAGCTGGCATAGTAAATCATTAGACTCTCAAGTTGCCTTGAGATGTGGCGCTTATCCTACTTGTTCTCACCAAACGAATGCACTAGCTTTTAGAGGCAGTGAATCAATGGGCCATTCTTTGAAATTCCCATTTGGAAATTCTTCTGCTAAAACAAGACTAAGGAATCATATCCGCCCTCCTTTGCGGGTTGTCTGTATGGACTATCCAAGACCGGACCTTGATAACACGGTGAATTTCTTAGAGGCTGCCTTGTTATCTTCATCCTTTCGTTCTTCTCCGCGTCCAGCTAAACCATTAAATGTTGTCATTGCTGGTGCAGGTTTGGCGGGTTTATCGACTGCAAAATACTTGGCAGATGCGGGCCATAAGCCTATATTGCTTGAAGCAAGAGATGTTTTAGGTGGAAAGGTGGCTGCATGGAAAGATGATGATGGAGACTGGTACGAGACAGGCTTGCATATATTCTTTGGGGCATATCCAAATGTGCAGAATCTTTTTGGTGAACTTGGTATCAATGATAGGTTGCAATGGAAGGAGCATTCTATGATATTTGCAATGCCAAATAAGCCAGGAGAATTCAGTCGATTTGATTTTCCTGAAGTTCTCCCTGCACCATTAAATGGGATATTGGCCATTTTAAAGAACAATGAAATGCTGACTTGGCCAGAGAAAGTGAAGTTTGCAATTGGGCTACTTCCAGCAATTGTTGGTGGACAGGCTTATGTTGAGGCTCAAGATGGTTTAAGTGTTCAAGAGTGGATGAGAAAGCAGGGTGTACCTGATAGAGTGACTACTGAGGTGTTTATTGCCATGTCAAAGGCTCTAAACTTTATTAACCCAGATGAGCTTTCAATGCAATGCATTTTGATAGCTTTGAACAGATTTCTTCAGGAGAAACATGGTTCAAAGATGGCTTTCTTGGATGGTAATCCCCCAGAGAGGCTCTGCATGCCAATTGTTGATCATATTCAGTCGCGTGGTGGTGAAGTCAAGCTTAATTCTCGGATAAAGAAGATTGAGCTAAATGATGATGGAACAGTGAAGAGTTTTTTACTAAATACTGGGGATGTGATTGAAGGGGATGTTTATGTGTTTGCCACTCCAGTTGATATCCTGAAGCTTCTTTTGCCTGATAACTGGAAAGAGATTCCTTACTTCAAGAAACTGGAGAAATTAGTTGGAGTTCCTGTTATTAATGTTCACATATGGTTTGACAGGAAACTGAAGAATACATACGATCACCTACTTTTCAGCAGGAGTCCTCTTCTCAGTGTGTATGCTGACATGTCTCTGACATGTAAGGAATATTATGACCCAAATAAATCCATGCTGGAGTTAGTTTTTGCGCCTGCTGAAGAATGGATTTCACGCAGTGACTCAGAGATTATTGATGCTACAATGGGGGAACTCGCAAAACTTTTTCCTGATGAAATATCCGCAGATCAAAGCAAAGCAAAAATCGTGAAGTATCATGTTGTTAAAACTCCAAGGTCGGTTTACAAGACTGTCCCAGATTGTGAACCTTGCCGTCCCTTGCAAAGATCTCCGATAGAGGGTTTCTATTTAGCTGGTGACTACACAAAACAAAAGTACTTGGCTTCAATGGAAGGTGCTGTTCTATCAGGGAAGCTTTGTGCACAGGCAATTATACAGGATTACGAGTTCCTGGTTGCTCGGGGGCAAGGAAGCTTGACTGAGGCAACCATTAGTTAA

***>Populus trichocarpa***

ATGAGTGCATTGAACTTGAGCTGGCATAGTAAATCATTAGACTCTCAAGTTGCCTTGAGATGTGGCGCTTATCCTACTTGTTCTCACCAAACTAATGCACTAGCTTTTAGAGGCAGTGAATCAATGGGCCATTCTTTGAAATTCCCATTTGGAAATTCTTCTGCTAAAACAAGACTAAGGAATCATATCCGCCCTCCTTTGCGGGTTGTCTGTGTGGACTATCCAAGACCGGACCTTGATAACACGGTGAATTTCTTAGAAGCTGCCTTGTTATCTTCATCCTTTCGTTCTTCTCCGCGTCCAGCTAAACCATTAAATGTTGTCATTGCTGGTGCAGGTTTGGCGGGTTTATCGACTGCAAAATATTTGGCAGATGCAGGCCATAAGCCTATATTGCTTGAAGCAAGAGATGTTTTAGGTGGAAAGGTGGCTGCATGGAAAGATGACGATGGAGACTGGTACGAGACAGGCTTGCATATATTCTTTGGGGCATATCCAAATGTGCAGAATCTTTTTGGTGAACTTGGTATCAATGATAGGTTGCAATGGAAGGAGCATTCTATGATATTTGCAATGCCAAATAAGCCAGGAGAATTCAGTCGATTTGATTTTCCTGAAGTTCTCCCTGCACCATTAAATGGGATATTAGCCATTTTAAAGAACAATGAAATGCTGACTTGGCCAGAGAAAGTGAAGTTTGCAATTGGGCTACTGCCAGCAATTGTTGGTGGACAAGCTTATGTTGAGGCTCAAGATGGTTTAAGTGTTCAAGAGTGGATGAGAAAGCAGGGTGTACCTGATAGAGTGACTACTGAGGTGTTTATTGCCATGTCAAAGGCTCTAAACTTTATTAACCCAGATGAGCTTTCAATGCAATGCATTTTGATAGCTTTGAACAGATTTCTTCAGGAGAAACATGGTTCAAAGATGGCTTTCTTGGATGGTAATCCCCCAGAGAGGCTCTGCATGCCAATTGTTGATCATATTCAGTCGCGTGGTGGTGAAGTCAAGCTTAATTCTCGGATAAAGAAAATTGAGCTAAATGATGACGGAACAGTGAAGAGTTTTTTACTAAATACCGGGGATGTGATTGAAGGGGATGTTTATGTGTTTGCCACTCCAGTTGATACCCTGAAGCTTCTTTTGCCTGATAACTGGAAAGAGATTCCTTACTTCAAGAAACTGGAGAAATTAGTTGGAGTTCCTGTTATTAATGTTCACATATGGTTTGACAGGAAACTGAAGAATACATACGATCACCTACTTTTCAGCAGGAGTCCTCTTCTCAGTGTGTATGCTGACATGTCTCTGACATGTAAGGAGTATTATGACCCAAATAAATCTATGCTGGAGTTAGTTTTTGCGCCTGCTGAAGAATGGATTTCACGCAGTGACTCAGAGATTATTGATGCTACAATGGGGGAACTTGCAAAACTTTTTCCTGATGAAATATCCGCAGATCAAAGCAAAGCAAAAATCGTGAAGTATCATGTTGTTAAAACTCCAAGGTCGGTTTACAAGACTGTCCCAGATTGTGAACCTTGCCGTCCCTTGCAAAGATCTCCGATAGAGGGTTTCTATTTAGCTGGTGACTACACAAAACAAAAGTACTTGGCTTCAATGGAAGGTGCTGTTCTATCAGGGAAGCTTTGTGCACAGGCAATTGTACAGGATTATGAGTTCCTGGTTGCTCGGGGGCAAGGAAGGTTGACTGAGGCAACCATTACTTAA

***>Populus euphratica***

ATGAGTGCATTGAACTTGAGCTGGTATAGTAAATCATTAGACTCTCAAGTTGCCTTGAGATGTGGCTCTTATCCTTCTTGTTCTCACCAAACTAATGCACTAGCTTTTAGAGGCAGTGAATCAATGGGCCATTCTTTGAAATTCGCATTTGGAAATTCTTCTGCTAAAACAAGACTAAGGAATCATATCCGCCCTCCTTTGCGGGTTGTCTGTGTGGACTATCCAAGACCGGACCTTGATAACACGATGAATTTCTTAGAAGCTGCCTTGTTATCTTCATCCTTTCATTCTTCTCCGCGTCCAGCTAAACCATTAAAGGTTGTCATTGCTGGTGCAGGTTTGGCGGGTTTATCGACTGCAAAATATTTGGCAGATGCAGGCCATAAGCCTATATTGCTTGAAGCAAGAGATGTTTTAGGTGGAAAGGTGGCTGCATGGAAGGATGATGATGGAGACTGGTACGAGACAGGCTTGCATATATTCTTTGGGGCGTATCCAAATGTGCAAAATCTTTTTGGTGAACTTGGTATCAATGATAGGTTGCAATGGAAGGAGCATTCTATGATATTTGCAATGCCAAATAAGCCAGGAGAATTCAGTCGATTTGATTTTCCTGAATTTCTCCCTGCACCATTAAATGGGATATTGGCCATTTTAAAGAACAATGAAATGCTGACTTGGCCAGAAAAAGTGAAGTTTGCAATTGGGCTACTGCCAGCAATAGTTGGTGGACAGGCTTATGTTGAGGCTCAAGATGGTTTAAGTGTTCAAGAGTGGATGAGAAAACAGGGTGTACCTGATAGAGTGACTACTGAGGTGTTTATTGCCATGTCAAAGGCTCTAAACTTTATTAACCCAGATGAGCTTTCAATGCAATGCATTTTGATAGCCTTGAACAGATTTCTTCAGGAGAAACATGGTTCAAAGATGGCTTTCTTGGATGGTAATCCCCCAGAGAGGCTCTGCATGCCAATTGTTGATCATATTCAGTCACGTGGTGGTGAAGTCAAGCTTAATTCTCGGATAAAGAAAATTGAACTAAATGATGATGGAACAGTGAAGAGCTTTTTACTAAATACTGGGGATGTGATTGAAGGGGATGTTTATGTGTTTGCCACTCCAGTTGATACCTTGAAGCTTCTTTTGCCTGAGAACTGGAAAGAGATTCCTTACTTCAAGAAACTGGAGAAATTAGTTGGAGTTCCTGTTATTAATGTTCACATATGGTTTGACAGGAAACTGAAGAATACAAACGATCACCTACTTTTCAGCAGGAGTCCACTTCTCAGTGTGTATGCTGACATGTCTCTGACATGTAAGGAATATTATGACCCAAATAAATCTATGCTGGAGTTGGTTTTTGCACCTGCTGAAGAATGGATTTCACGCAGTGACTCAGAGATTATTGACGCTACAATGGGGGAACTTGCAAAACTTTTTCCTAATGAAATATCGGCAGATCAAAGCAAAGCAAAAATCGTGAAGTATCATGTTGTTAAAACTCCAAGGTCGGTTTACAAAACTGTCCCAGATTGTGAACCTTGCCGTCCCTTGCAAAGATCTCCAATAGAGGGTTTCTATTTAGCTGGTGACTACACAAAACAGAAGTACTTGGCTTCAATGGAAGGTGCTGTTCTATCAGGGAAGCTTTGTGCACAGGCAATTGTACAGGATTATGAGTTCCTGGTTGCTCGGGGGCAAGGAAGGTTGACCGAGGCAACCATTACTTAA

***>Paeonia ostia***

ATGGCCCTTTATGGTTGTGTTTCCGCGGTGACGCCAACGCCAAGTAATAAAATCTCGCAATCCACCTTGACTCGTGGTTTTCGCATGAAAATCAATCCCGCGATGGCATTTGGAGATAGTGCTGCTATGGGTCTCAGCTTGAGAATTCCAAATACACACGCCATAACTACGAGGCCTAGAAAAGATGTCTTCCCTTTGCAGGTTGTTTGCGTGGACTATCCAAGACCAGAGCTTGACAATACTGTTAATTTCTTAGAAGCCGCTTACTTATCATCATTCTTCCGCTCTTCTTCCCGTCCAAATAAACCGTTGGATGTTGTGATTGCCGGTGCAGGTTTGGCTGGTTTATCAACTGCAAAATATTTAGCAGATGCAGGTCACAGACCTTTATTGTTGGAAGCAAGAGATGTTCTAGGTGGAAAGGTGGCTGCATGGAAAGATGACGATGGAGACTGGTATGAGACAGGGCTACATATATTCTTTGGGGCTTACCCAAATGTGCAGAACCTGTTTGGAGAACTTGGTATTAATGATCGGTTGCAGTGGAAGGAGCATTCTATGATATTTGCCATGCCTAACAAGCCAGGAGAATTCAGCCGATTTGATTTCCTTGAAGTACTGCCTGCACCTTTAAATGGCCTTTGGGCGATCCTGAAGAACAATGAAATGTTGACTTGGCCAGAAAAAGTGAAATTTGCGATTGGACTCTTGCCAGCAATTGTTGGCGGTCAGGCTTATGTTGAGGCTCAAGATGGTTTTACTGTTAAAGACTGGATGAGAAAACAAGGGATACCTGATCGAGTAACTAATGAGGTGTTTATTGCCATGTCAAAGGCACTAAACTTCATAAACCCAGATGAACTTTCAATGCAATGTATTTTGATTGCTTTGAACAGATTTCTTCAGGAGAAGCATGGTTCCAAGATGGCTTTCTTAGATGGCAATCCTCCAGAGAGACTCTGCATGCCAATTGTTGATCATATTGAGTCATTGGGGGGTCAGGTCCGTCTTAATTCAAGAATACAAAAGATTGAGTTGAATAAAGATGGAACCGTGAAGGGCTTTTTGCTTAATGATGGGAATTTAATTAAAGGAGATGCTTATGTATTCGCCACTCCAGTTGACATTCTGAAGCTTCTTCTGCCGAAAGAGTGGAAAGAGATTCCAGACTTTAAAAGACTGGAGAAGTTAGTTGGAGTTCCAGTTATAAATGTTCACATATGGTTTGACAGGAAGTTGAAGAACACATATGACCATTTACTTTTCAGCAGAAGTCCCCTTCTGAGTGTGTATGCTGACATGTCTGTAACTTGTAAGGAATATTACAACCCAAATGAATCTATGCTGGAGTTGGTTTTTGCTCCTGCAGAAGAATGGATCTCACGTAGTGACTCAGAAATTATTGATGCTACGATGAAAGAACTTGCAAAACTGTTTCCTGATGAAATTTCTGCGGATCAGAGCAAGGCTAAGATTTTGAAGTATCATGTTGTTAAAACACCGAGGTCCGTATATAAGACTGTCCCAGATTGTGAACCATGTCGTCCCTTACAAAGATCTCCAATAGAAGGATTCTATTTAGCAGGTGACTATACAAAACAGAAGTATTTGGCTTCTATGGAAGGTGCTGTTCTATCGGGAAAGTTTTGTGCACAGGCTATTGTGCAGGATTATGAATTGCTTGTTGCTCGGGAGCCGAAAAAATTGGCTGAGGTTCGCACCCTCTAA

***>Camellia sinensis***

ATGTCTCAATTTGGACAAGTTTCCACCGTCAGTGTGAGTGGGCAAAACAATGGAATAAGTGTTTGGAACCCAAAATCTACTTGGGGGTGTGGTTGTTCCTTTGGTTCAGGGCCAGCCAAAGCACTATCATTTCGAGGGAGTGATTCCATGGGTCATAGGTTCAAAATTCCTAATGCATATGCTGTTGGAACCAGACCAAGGAAGGACGTGTGCCCTTTGAAGGTGGTTTGCATTGACTATCCAAGACCAGACCTTGAGAGTACTGTCAATTTTTTGGAAGCTGCCTACTTATCTTCAGCCTTTCGTACTTCCCGCCGTCCAGATAAACCATTGAAGGTTGTTATTGCYGGTGCAGGTTTGGCTGGTTTGTCTACTGCAAAATATTTGGCAGATGCAGGTCACAAACCTGTATTATTGGAAGCAAGGGATGTTTTAGGTGGAAAGGTGGCTGCGTGGAAAGATGATGATGGAGACTGGTATGAGACTGGCTTACATATATTTTTTGGGGCTTACCCAAATGTGCAGAACCTGTTTGGAGAACTTGGTATAAATGATCGATTGCAGTGGAAAGAGCATTCTATGATATTTGCAATGCCAAACAAGCCAGGGGAGTTCAGCCGATTTGACTTTCCTGAAGTTCTACCTGCACCATTAAATGGGATATGGGCCATATTAAAGAACAATGAAATGCTTACTTGGCCTGAGAAAATCAAGTTTGCAATTGGACTCATTCCAGCAATTCTAGGTGGACAGGCCTATGTTGAAGCTCAAGATGGTTTAAGTGTTAAAGACTGGATGAGGAAGCAAGGTATACCAGATCGAGTAACTACTGAGGTGTTTATTGCCATGTCAAAAGCGTTAAACTTCATAAACCCTGATGAACTTTCAATGCAGTGTATTTTGATTGCACTGAACCGGTTTCTTCAGGAGAAGCATGGTTCGAAGATGGCATTCTTGGATGGTAACCCCCCAGAGAGACTTTGCCAGCCAATTGTTGATCATATTCAGTCACTGGGTGGTGAAGTCCAACTTAATTCTCGAATTAAAAAGATTGAGCTGAATAAAGATGGAACTGTAAAGAGCTTTTTACTAAATAATGGTAATGCTATTGAAGGAGATGCCTATGTTTTTGCTACTCCAGTTGATATCTTGAAGCTTCTTTTGCCTGAAGACTGGAAAGAGATTCCGTACTTCAGAAAATTGGAGATATTAGTTGGAGTTCCTGTTATAAATGTTCACATATGGTTTGACAGGAAGCTAAGGAATACTTATGATCATCTACTTTTTAGCAGAAGTCCTCTTCTCAGTGTGTATGCTGACATGTCAGTGGCATGTAAGGAATATTACGACCCAAATCGCTCTATGCTGGAATTGGTTTTTGCACCTGCAGAGGAATGGATCTCATGTAGTGATGAGGAAATTATTGATGCTACGATGAAGGAACTGGCAAAACTCTTTCCTGATGAAATTTCTGCAGATCAGAGCAAAGCAAAAATATTGAAGTACCATGTTGTTAAAACACCAAGGTCTGTTTATAAAACTGTCCCGAACTGTGAACCTTGTCGTCCATTGCAAAGATCCCCTGTAGAAGGGTTCTATTTGTCTGGTGACTACACAAAGCAAAAATATTTGGCTTCAATGGAAGGTGCTGTTCTTTCAGGAAAGCTTTGTGCACAAGCTATTGTACAGGATTATGAGAAGCTTGTTTCCCGGGAGCAGGGAAAGCTGGCCGAGGCAAGTGTCGTGTAA

***>Prunus avium***

ATGTCTCAGTGGGCTTGTGTCTCTGCTGCTAACTTGAGCTGCCAAGCTAGCATCATCAACACTCAAAAGCTACGAAACACTCCCAGATGCGATGCCTTTTCATTTAAAGGTAGTGAATTTATGGCTCAAAGCTGTAGATTTTTAAGCCCACAAGCCATTCATGGAAGGCCGAGGAATGGTGCTTGCCCTTTGAAGGTGGTTTGCGTTGATTATCCAAGACCAGACCTTGACAATACTGCTAATTTCTTAGAAGCTGCATATTTCTCTTCCACTTTCCGAGCCTCTCCTCGTCCAGCTAAGCCGTTGAAGGTCGTGATTGCTGGTGCAGGTTTGGCTGGTCTGGCAACTGCAAAATATTTGGCTGATGCAGGTCATAAACCTATCTTACTGGAAGCAAGAGATGTTCTAGGCGGAAAGGTGGCAGCATGGAAAGATAAGGATGGAGACTGGTACGAAACAGGCCTACATATCTTCTTTGGGGCTTATCCGAATATTCAGAACCTGTTTGGTGAGCTTGGTATTGATGATAGATTGCAGTGGAAGGAGCATTCTATGATATTTGCAATGCCAAACAAACCAGGAGAGTTCAGCCGGTTTGATTTCCCTGAAGTTTTACCAGCACCCTTAAATGGAATATGGGCCATATTGAAGAACAATGAGATGCTGACTTGGCCAGAGAAAATCAAGTTTGCAATTGGACTACTGCCAGCAATTCTTGGTGGGCAGGCTTATGTTGAAGCCCAAGATGGCTTGAGTGTAAAAGATTGGATGAGGAAACAGGGCATACCAGATCGAGTGACCACTGAGGTGTTTATTGCCATGTCAAAGGCCCTGAACTTTATTAACCCTGATGAACTTTCAATGCAATGCATATTGATTGCTTTGAACCGATTCCTTCAGGAGAAACACGGTTCCAAGATGGCTTTCTTGGATGGTAGTCCCCCTGAGAGACTCTGTGCACCAATTGTTGATCATATCCAGTCATTGGGCGGTGAAGTCCGAATTAATTCCAGAATACAGAAAATTGAGCTAAATAACGATGGGACCGTGAAGAGTTTTGTACTAAATAATGGGAGCATGATTGAAGCAGATGCCTATGTATTCGCCACTCCAGTTGATATCCTAAAGCTTCTATTGCCTGATAACTGGAAAGAGATCCCATATTTCAAGAAATTGAAGAAACTAGTTGGCGTTCCAGTTATCAATGTTCACATATGGTTTGACAGAAAGCTGAAGAACACATATGATCATCTACTTTTTAGCAGAAGTCCTCTTTTAAGTGTCTATGCTGACATGTCCGTAACATGTAAGGAATATTACAATCCAAACCAGTCTATGCTGGAGTTGGTTTTTGCACCAGCAGAAGAATGGATTTCATGCAGTGATTCAGAAATTATTGATGCTACACTCAAAGAACTTGCAAAACTCTTTCCTGATGAGATAGCTGCAGATCAAAGCAAAGCAAAGATTTTGAAGTACCATGTTGTGAAAACACCAAGGTCGGTTTACAAAACTGTACCAGATTGCGAACCTTGCCGTCCCTTGCAAAGATCTCCCCTAGAGGGTTTCTATTTAGCTGGTGATTATACAAAACAAAAGTATTTAGCCTCAATGGAAGGTGCTGTTCTGTCAGGGAAACTTTGTGCACAAGCAATTGTACAGGATTACGAATTGCTTGTTGCTCGGGGACAAACAAGGTTGGCTGAGGCAAGCGTTCGGTGA

***>Ziziphus jujuba***

ATGTCCCAGTGGGGATGTGTTTCCGCGGCTAACTTGAGCTGGCAAACTAGTAGTATCGTAAGTATTAGGAAGGTTGGAAGCACACCCAGATGTTGTTTCCAAATGGGTTTGCCAAATTTGGAAGCTTTGACTTTTGGAGGTAGTGAATTTATGTCTCAGAGTTTGAGAATTCCGTGCTCAAGTGCTACTGGTAGAGGGCAGAGGAAAAGGGGTTTCCCTTTGAAGGTAGTTTGTGTGGACTATCCAAGACCGGAGCTTGAGAATACTGTTAATTTCTTAGAAGCTGCTTCCTTGTCTGCTTCCTTTCGCTCTTCTCCTCGTCCTGCTAAACCGTTAAAAGTTGTAATTGCTGGTGCAGGACTGGCTGGTTTATCAACTGCAAAGTATTTGGCAGATGCAGGTCATAAACCTTTATTACTGGAAGCAAGAGATGTTTTAGGTGGAAAGCTGGCAGCATGGAAAGATGAGGATGGAGACTGGTATGAGACAGGCCTACATATATTCTTTGGAGCGTATCCAAATGTACAAAACTTGTTTGGAGAGCTTGGTATTGATGACAGGTTACAGTGGAAAGAGCATTCTATGATATTTGCAATGCCCAACAAGCCAGGAGAGTTCAGTCGATTTGACTTCCTTGAAGCTTTGCCATCACCCATAAATGGAATATGGGCCATTTTGAAGAATAATGAAATGCTGTCTTGGCCAGAGAAAGTAAAGTTTGCTATTGGTCTTCTACCGGCAATGCTTGGTGGACAGGCTTATGTTGAAGCTCAAGATAACATCTCTGTTAAAGATTGGATGAGAAAACAGGGCATACCTGATCGAGTAACTGAGGAGGTGTTTATTGCCATGTCAAAGGCATTAAACTTTATTAACCCTGATGAACTTTCAATGCAATGTATATTGATTGCTTTGAACAGATTTCTTCAGGAGAAGCATGGTTCCAAGATGGCCTTTTTAGATGGTAATCCTCCAGAGAGACTCTGTATGCCAATTGTTGATCATATCGAGAAATTGGGTGGTGAACTCCAACTTAATTCACGGATACAAAAAATTGAGCTAAATAATGACGGAACAGTGAAGAGATTTTTATTAACCAATGGAAATGTGATTGAAGGGGATGTTTATGTGTTTGCCACTCCAGTTGATATCCTGAAGCTTCTCTTGCCTGACAGCTGGAAAGAAATTCCATATTTTAAAAAGTTGGAGAAATTAGTAGGAGTCCCAGTTATTAATGTTCACATATGGTTTGACAGAAAACTGAAGAACACATATGACCACCTACTTTTTAGCAGAAGTCCTCTTCTAAGTGTCTATGCTGACATGTCTGTAACATGCAAGGAATATTACAATCCAAACCAGTCTATGTTGGAGTTGGTTTTTGCACCAGCAGAAGAATGGATATCCCGTAGTGACACAGAAATTATTGATGCTACGATGAAGGAACTTGCTAAACTCTTTCCTGATGAAATATCCGCCGATCAGAGCAAAGCAAAGATTTTAAAGTATCATGTTGTCAAAACACCGAGGTCTGTCTACAAAACCGTCCCTGATTGTGAACCTTGCCGGCCCTTGCAAAAATCTCCCGTAGAGGGTTTTTATTTGGCTGGTGACTACACAAAACAGAAATATTTAGCTTCGATGGAAGGTGCAGTTCTATCGGGAAAGCTTTGTGCACAGGCAATTGTACAGGATTACGATTCACTTATTGCTCGTGAGCAAAGGAACTTGGCCGAGGCAGTCAGTCGTTGA

***>Malus domestica***

ATGGCGCAGTGGGCTTGTGTCTCCGCTGCTAACTTGAGCTGCCAAGCTACCATCGTAAACACTCAAAAGCAACGAAACAGTCCCGGATGCGATGCCCTTTCTTTCAAAGGCAGTGAATTTATGGCTCAGAGCTGTAGATTTTCAAGCCCACAAGCTGTTTATAGAAGGCCCAGGAATGGTGTTTGCCCCTTGAAGGTGGTTTGCGTTGATTATCCAAGACCAGACCTTGACAGTACTGCTAATTTCTTAGAAGCTGCGTACTTCTCTTCCACTTTCCGAGCCTCTCCTCGTCCAACCAAGCCGTTAAAAGTTGTGATTGCTGGTGCAGGTTTGGCTGGTCTGGCAACTGCAAAATATTTGGCGGATGCGGGTCATCAACCTATACTACTAGAAGCGAGAGATGTTTTAGGCGGAAAGGTGGCAGCATGGAAAGATAGTGATGGGGACTGGTATGAAACAGGCCTGCATATATTCTTTGGGGCATATCCAAATATTCAGAATCTGTTTGGAGAGCTTGGTATTAATGATCGGTTGCAGTGGAAGGAACATTCTATGATATTTGCAATGCCAAACAAGCCAGGGGAGTTCAGTCGGTTTGATTTCCTGGAAGTTCTGCCAGCACCCATAAATGGAATATGGGCCATATTGAAGAACAATGAGATGCTGACTTGTCCAGAGAAAATCAAGTTTGCAATTGGACTACTGCCAGCAATCCTTGGTGGGCAGGCTTATGTTGAAGCCCAAGATGGCTTGAGCGTAAAAGACTGGATGAGGAAACAGGGCATACCTGATCGAGTAACTACAGAGGTGTTTATAGCCATGTCAAAGGCCCTTAACTTTATTAACCCTGATGAACTTTCAATGCAGTGCATATTGATTGCTTTGAACCGGTTCCTCCAGGAGAAACACGGTTCCAAGATGGCTTTCTTGGATGGTAGTCCCCCCGAGAGACTCTGTGCTCCAATTGTTGATCATATCCAGTCATTGGGCGGTGAAGTCCGAACTAATTCCCGAATACAGAAAATTGATCTAAATAACGATGGAACTGTGAAGAGTTTTGTACTAAATAATGGGAGCGTGATTGAAGCAGATGCGTATGTGTTCGCCACTCCAGTTGATATCCTAAAGCTTCTATTGCCTGAAAACTGGAAAGAGATGCCATATTTCAAGAAATTGGAGAAATTAGTTGGAGTTCCAGTTATCAATGTTCACATATGGTTCGACAGAAAGCTGAAGAACACATATGATCACCTACTTTTTAGCAGAAGTCCTCTTTTAAGTGTGTATGCTGACATGTCCGTAACATGTAAGGAATATTACAATCCAAACCAATCTATGCTGGAGTTGGTTTTTGCACCGGCAGAAGAATGGATTTCATGTAGTGATTCTGAAATTATTGATGCTACACTCAAAGAACTTGCAAAACTCTTTCCTGACGAAATAGCTGCAGATCAGAGCAAAGCAAAGATTTTGAAGTACCATGTTGTGAAAACACCAAGGTCTGTTTACAAGACTGTACCAGGTTGTGAACCTTGCCGTCCCTTGCAAAGATCTCCCCTAGAGGGTTTCTATTTAGCTGGTGATTACACAAAACAAAAGTATTTAGCCTCAATGGAAGGAGCAGTTCTATCAGGGAAACTTTGTGCTCAGGCGATTGTACAGGATTATGAATTGCTTGCTGCCCGGGGAATAAAAACAACGTTGGCTGAGGCAGCCGCTCGATGA

***>Durio zibethinus***

ATGATTTTATGTCGTGTTTCCCTCTATTTCGGCCAAATCCACTCTAAAAAAAAATACGTACACGTTAACTTATCACCTCCACAACTTCCTCTGCCTGTGTCCCGAACCACTTTCCATATCCCACTTTCTATTATCAAGGCAAATCAAGTTTTTTTAACAATTTTTTTTGAAATAATTCAATCTACCCCTTCTTTGGCGTTGATTTGTTGGTGTTCGAAAATGAGTCTCTGTGGGAGTGTTTCTGCTGTGCATTTGAACTTCCAAAGCAACACAATAAGCATGGGAAGCGTCTTAGCTTTTAGAAGTGGTGAATCCATGGGACATACCTTGAGAATTCCTTTTAAAAAGGGGTCAAGTAAGGGTGCTTGCCCTTTGCAGGTGCTTTGTATAGATTATCCAAGACCTGAGCTTGAGAATACTGTTAACTTTTTGGAGGCTGCGTCTTTATCTGCTTCTTTTCGTTCTGCTCCCCGTCCAACTAAGCCATTGAAAGTCATAATTGCTGGTGCAGGTTTGGCTGGTTTGTCAACTGCAAAATATTTAGCAGATGCAGGTCACAAACCTCTGTTGCTAGAAGCAAGAGATGTTCTAGGTGGAAAGGTGGCTGCATGGAGAGATGAGGATGGAGATTGGTATGAGACAGGCCTACATATATTCTTTGGGGCTTACCCAAATGTGCAAAACCTGTTTGGAGAACTTGGCATCAATGATCGGTTGCAATGGAAGGAGCATTCTATGATATTTGCAATGCCAAATAAACCTGGAGAGTTCAGCCGATTTGATTTTCCAGAAGTTCTACCTGCACCTTTAAATGGGATATGGGCCATTTTGAAGAACAATGAAATGCTGACTTGGCCAGAGAAAGTGAAGTTTGCAATAGGACTCCTACCAGCAATGCTTGGTGGACAACCTTATGTTGAGGCCCAAGATGGTTTAAGTGTTAAAGAGTGGATGAAAAAGCAGGGTGTACCTGATCGTGTGACTAACCAGGTGTTTATTGCCATGTCAAAGGCACTGAACTTCATTAACCCAGATGAACTTTCAATGCAATGTATACTGATTGCTTTAAACAGATTTCTTCAGGAGAAAAATGGATCCAAGATGGCATTCTTGGATGGCAACCCTCCAGAGAGGCTTTGCATGCCTATTGTTAATCATATTGAATCACTGGGTGGCGAGGTCCGGCTGAACTCACGAATAAAGAAAATAGAGCTTAATGATGATGGGACTGTGAAGAGTTTTCTTCTAACTGATGACAATACAATTGAAGGAGATGCTTATGTAATTGCAACTCCAGTTGATATCCTAAAGCTACTTTTGCCTGAAGACTGGAGAGAGATTTCATACTTCAAGAAATTAGAGAAATTAGTTGGCGTTCCAGTTATCAATGTTCACATATGGTTTGATAGGAAATTGAAGAACACCTATGATCATCTACTCTTTAGCAGAAGTCCCCTTTTAAGTGTATATGCCGACATGTCCGTAACATGTAAGGAATATTACAATCCAAACCAATCCATGTTGGAGTTAGTTTTTGCTCCTGCAGAAGAATGGATTGCACGAAGTGACTCAGAAATTATTGACGCTACAATGAAGGAACTTGCAAAGCTCTTTCCTGATGAAATTTCTGCAGATCAGAGCAAAGCAAAAGTCGTAAAGTACCATGTTGTTAAAACACCAAGATCTGTATATAAAACTGTACCAAATTGTGAACCCTGCCGTCCCTTGCAAAGATCTCCAATAGAGGGATTCTATCTAGCGGGTGATTACACAAAGCAAAAGTATTTAGCTTCGATGGAAGGTGCTGTTCTTTCAGGGAAGCTTTGTGCAGAGTCTATTGTACAGGATTATGAATTGCTTTGTACTTTGGGGCAAAGAAAGTTGACAGGAGCAAGGGTTCACTGA

***>Pyrus x bretschneideri***

ATGGCGCAGTGGGCTTGTGTCTCCGCTGCTAACTTGAGCTGCCAAGCTACCATCGTAAACACTCAGAAGCAACGAAACAGTCCCCGATGCGATGCCTTTTCTTTCAAAGGCAGTGAATTTATGGCTCAGAGCTGGAGATTTTCAAGCCCACAAGCTGTTTATAGAAGGCCCAGGAATGGTGTTTGCCCTTTGAAGGTCGTTTGCGTTGATTATCCAAGACCAGACCTTGACAGTACTGCTAATTTCTTAGAAGCTGCGTACTTCTCTTCCACTTTCCGAGCCTCTCCTCGTCCAACCAAGCCGTTAAAAGTTGTGATTGCTGGTGCAGGTTTGGCTGGTCTGGCAACTGCAAAATATTTGGCGGATGCGGGTCATCAACCTATACTACTAGAAGCCAGAGATGTTTTAGGCGGAAAGGTGGCAGCATGGAAAGATAGTGATGGGGACTGGTACGAAACAGGCCTCCATATATTCTTTGGAGCATATCCAAATATTCAGAACCTGTTTGGAGAGCTTGGTATTAATGATCGATTGCAGTGGAAGGAACATTCTATGATATTTGCAATGCCAAACAAGCCAGGGGAGTTCAGTCGGTTTGATTTCCTAGAAGTTCTGCCAGCACCCATAAATGGAATATGGGCCATATTGAAGAACAATGAGATGCTGACTTGGCCAGAGAAAATCAAGTTTGCAATTGGACTACTGCCAGCAATCCTTGGTGGGCAGGCTTATGTTGAAGCCCAAGATGGCTTGAGCGTAAAAGACTGGATGAGGAAACAGGGCATACCTGATCGAGTAACTACTGAGGTGTTTATAGCTATGTCAAAGGCCCTTAACTTTATTAACCCTGATGAACTTTCAATGCAATGCATATTGATTGCTTTGAACCGATTTCTTCAGGAGAAACACGGTTCCAAGATGGCTTTCTTGGATGGTAGTCCCCCTGAGAGACTCTGTGCTCCAATTGTTGATCATATCCAGTCATTGGGCGGTGAAGTCCGAACTAATTCCCGAATACAGAAAATTGATCTAAATAATGATGGAACTGTAAAGAGTTTTGTACTAAATAATGGGAGTGTGATTGAAGCAGATGCGTATGTGTTTGCCACTCCAGTTGATATCTTAAAGCTTCTATTGCCTGAAAACTGGAAAGAGATGCCATATTTCAAGAAATTGGAGAAACTAGTTGGAGTTCCAGTTATCAATGTTCACATATGGTTTGATAGAAAGCTGAAGAACACATATGATCACCTACTTTTTAGCAGAAGTCCTCTTTTAAGTGTGTATGCTGACATGTCCGTAACATGTAAGGAATATTACAATCCAAACCAATCTATGCTGGAGTTGGTTTTTGCACCGGCAGAAGAATGGATTTCATGTAGTGATTCAGAAATTATTGATGCTACCCTCAACGAACTTGCAAAACTCTTTCCTGACGAAATAGCTGCAGATCAAAGCAAAGCAAAGATTTTGAAGTACCATGTTGTGAAAACACCAAGGTCTGTTTACAAGACTGTACCAGGTTGTGAACCTTGCCGTCCCTTGCAGAGATCTCCCCTAGAGGGTTTCTATTTAGCTGGTGATTACACAAAACAAAAGTATTTAGCCTCAATGGAAGGAGCGGTTCTATCAGGGAAACTTTGTGCTCAGGCGATTGTACAGGATTATGAATTGCTTGCTGCCCGGGGAAAAAAAACAAGGTTGCCTGAGGCAGCTGCTCGATGA

***>Rosa chinensis***

ATGTCGCAGTGGGCTTGTGTCTCTGCCACCAACTTGAGCTACCAAGCCAACCTCATCAACACCCAAAACCCACAAACCACTCCCAGATATGATGCGCTTTCCTTTCACGGCAGTGAAATTGTTGCTCGGAATTTTGGGTTTCTGAGCTCACAAGCTACTACTAGTATTGGTAAAAGGCTGAGGAAGGGTGCTCTCCCTTTGAAGGTGGTTTGTGTGGATTATCCAAGACCCGAGCTTGACAATACTGTAAATTTCTTAGAAGCTGCGCTCTTGTCTTCCTCTTTCAGAGCCTCTTCTCGCCCAGCTAAGCCCCTCAAGGTTGTGATTGCTGGTGCAGGTTTGGCTGGGTTGTCAACTGCAAAGTATTTGGCAGATGCAGGTCATAAACCCATACTACTGGAAGCAAGAGACGTTTTAGGTGGAAAGATTGCAGCATGGAAAGATAAAGATGGAGACTGGTATGAGACAGGCCTACATATATTTTTTGGGGCTTATCCAAATATTCAGAACTTGTTTGGAGAGCTTGGTATCGATGATCGGTTGCAGTGGAAGGAACACTCTATGATATTTGCAATGCCAAACAAGCCAGGAGAGTTCAGCCGGTTTGATTTCCCTGAAGTTCTGCCAGCACCCTTAAATGGAATATGGGCCATATTAAAGAACAATGAGATGCTGACATGGCCAGAAAAAGTGAAGTTTGCTATCGGACTTGTGCCAGCAATTCTTGGTGGACAGGCTTATGTTGAAGCTCAGGATGGCTTGACTGTAAAGGAGTGGATGAGAAAACAGGGGATACCTGATCGAGTAACTACTGAGGTGTTTATTGCCATGTCAAAGGCCCTTAACTTTATTAATCCTGATGAGCTCTCAATGCAATGCATATTGATTGCTTTGAATCGATTTCTTCAGGAGAAACACGGTTCCAAGATGGCTTTCCTTGATGGAAGTCCTCCCGAGAGACTCTGTCAACCAATCGTTGATCATATCCAGTCATTGGGCGGTGAAGTCCGGCTTAATTCCCGATTACAAAAGATTGAGCTAAATAATGATGGAACAGTGAAGAGCTTTGTACTACAAAATAACAGTGTGATTGAAGCGGATGCTTATGTATCTGCCTCTCCAGTTGATATCTTCAAGCTTCTAGTGCCTGAAAACTGGAAAGAGATTCCATATTTCAAGAAATTGGACAAACTAGTTGGAGTTCCAGTCATCAATGTACACATATGGTTTGACAGAAAACTAAAGAACACATATGATCACCTACTTTTTAGCAGAAGTCCTCTTCTAAGTGTGTATGCTGATATGTCGGTAACATGCAAGGAGTATTACAATCCAAATCAGTCTATGCTGGAGTTGGTTTTTGCACCAGCAGAAGAATGGATTTCACGCAGTGATTCGGAAATTATTGATGCTACGCTCAAAGAACTTGCAAAGCTCTTTCCCGATGAGATAGCTGCAGATCAAAGCAAAGCGAAGATTTTGAAGTACCATGTTGTGAAAACACCAAGGTCTGTGTACAAAACTATACCAGATTGTGAACCTTGCCGTCCGTTGCAAAGATCTCCCTTGGAGGGTTTCTATTTAACTGGTGACTATACAAAACAAAAATACTTAGCCTCTATGGAAGGTGCTGTTCTATCAGGGAAACTTTGTGCACAGGCGATTGTACAGGACTATGAATTGCTCGTTGCTCGGGGCCAGAAAAGGTTGGCTGAGGCAGGTGCTAGATGA

***>Prunus mume***

ATGTCTCAGTGGGCTTGTGTCTCTGCTGCTAACTTGAGCTGCCAAGCTAGCATCATCAACACTCAAAAGCTACGAAACACTCCTAGATGCGATGACTTTTCATTTAAAGGTAGTGAATTTATGGCTCAAAGCTGTAGATTTTTAAGCCCACAAGCTATTTATGGAAGGCCGAGGAATGGTGCTTGCCCTTTGAAGGTGGTTTGCGTTGATTATCCAAGACCAGACCTTGACAATACTGCTAATTTCTTAGAAGCTGCATATTTCTCTTCCACTTTCCGAGCCTCTCCTCGTCCAGCTAAGCCGTTGAAGGTCGTGATTGTTGGTGCAGGTTTGGCTGGTCTGGCAACTGCAAAATATCTGGCTGATGCAGGTCATAAACCTATCTTACTGGAAGCAAGAGATGTTCTAGGCGGAAAGGTGGCAGCATGGAAAGATAAGGATGGAGACTGGTACGAAACAGGCCTGCATATCTTCTTTGGGGCTTATCCGAATATTCAGAACCTGTTTGGTGAGCTTGGTATTGATGATCGATTGCAGTGGAAGGAGCATTCTATGATATTTGCAATGCCAAGCAAACCAGGAGAGTTCAGCCGGTTTGATTTCCCTGAAGTTTTACCAGCACCCTTAAATGGAATATGGGCCATATTGAAGAACAATGAGATGCTGACTTGGCCAGAGAAAATCAAGTTTGCAATTGGACTACTGCCAGCAATTCTTGGTGGGCAGGCTTATGTTGAAGCCCAAGATGGCTTGAGTGTAAAAGATTGGATGAGGAAACAGGGCATACCGGATCGAGTGACTACTGAGGTGTTTATTGCCATGTCAAAGGCCCTGAACTTTATTAACCCTGATGAACTTTCAATGCAATGCATATTGATTGCTTTGAACCGATTCCTTCAGGAGAAACACGGTTCCAAGATGGCTTTTTTGGATGGTAGTCCCCCTGAGAGACTCTGTGCACCAATTGTTGATCATATCCAGTCATTAGGCGGTGAAGTCCGAATTAATTCCCGAATACAGAAAATTGAGCTAAATAAAGATGGGACCGTGAAGAGTTTTGTACTAAATAATGGGAGCATGATTGAAGCAGATGCCTATGTATTCGCCACTCCAGTTGATATCCTAAAGCTTCTATTGCCTGATAACTGGAAAGAGATCCCATATTTCAAGAAATTGGAGAAACTAGTTGGCGTTCCAGTTATCAATGTTCACATATGGTTTGACAGAAAGCTGAAGAACACATATGATCATCTACTTTTTAGCAGAAGTGCTCTTTTAAGTGTCTATGCTGACATGTCTGTAACATGTAAGGAATATTACAATCCAAACCAGTCTATGCTGGAGTTGGTTTTTGCACCAGCAGAAGAATGGATTTCACGCAGTGATTCAGAAATTATTGATGCTACACTCAAAGAACTTGCAAAACTCTTTCCTGATGAGATAGCTGCAGATCAAAGCAAAGCAAAGATTTTGAAGTACCATGTTGTGAAAACACCAAGGTCGGTTTACAAAACTGTACCAGGTTGTGAACCTTGCCGTCCCTTGCAAAGATCTCCCCTAGAGGGTTTCTATTTAGCTGGTGATTACACAAAACAAAAGTATTTAGCCTCAATGGAAGGTGCTGTTCTGTCAGGGAAACTTTGTGCCCAAGCAATTGTACAGGATTACGAATTGCTTGTTGCTCGGGGACAAACAAGGGTGGCTGAGGCAAGCGTTCGGTGA

***>Prunus armeniaca***

ATGTCTCAGTGGGCTTGTGTCTCTGCTGCTAACTTGAGCTGCCAAGCTAGCATCATCAACACTCAAAAGCTACGAAACACTCCCAGATGCGATGCCTTTTCATTTAAAGGTAGTGAATTTATGGCTCAAAGCTGTAGATTTTTAAGCCCACAAGCTATTTATGGAAGGCCGAGGAATGGTGCTTGCCCTTTGAAGGTGGTTTGCGTTGATTATCCAAGACCAGACCTTGACAATACTGCTAATTTCTTAGAAGCTGCATATTTCTCTTCCACTTTCCGAGCCTCTCCTCGTCCAGCTAAGCCGTTGAAGGTCGTGATTGCTGGTGCAGGTTTGGCTGGTCTTGCAACTGCAAAATATTTGGCTGATGCAGGTCATAAACCTATCTTACTGGAAGCAAGAGATGTTCTAGGCGGAAAGGTGGCAGCATGGAAAGATAAGGATGGAGACTGGTACGAAACAGGCCTCCATATCCTCTTTGGGGCTTATCCGAATATTCAGAACCTGTTTGGTGAGCTTGGTATTGATGATCGATTGCAGTGGAAGGAGCATTCTATGATATTTGCAATGCCAAACAAACCAGGAGAATTCAGCCGGTTTGATTTCCCTGAAGTTTTACCAGCACCCTTAAATGGAATATGGGCCATATTGAAGAACAATGAGATGCTGACTTGGCCAGAGAAAATAAAGTTTGCAATTGGACTACTGCCAGCAATTCTTGGTGGGCAGGCTTATGTTGAAGCCCAAGATGGCTTGAGTGTAAAAGATTGGATGAGGAAACAGGGCATACCGGATCGAGTGACTACTGAGGTGTTTATTGCCATGTCAAAGGCCCTGAACTTTATTAACCCTGATGAACTTTCAATGCAATGCATATTGATTGCTTTGAACCGATTCCTTCAGGAGAAACACGGTTCCAAGATGGCTTTCTTGGATGGTAGTCCCCCTGAGAGACTCTGTGCACCAATTGTTGATCATATCCAGTCATTGGGCGGTGAAGTCCGAATTAATTCCCGAATACAGAAAATTGAGCTAAATAAAGATGGGACCGTGAAGAGTTTTGTACTAAATAATGGGAGCATGATTGAAGCAGATGCCTATGTATTTGCCACTCCAGTTGACATCCTAAAGCTTCTATTGCCTGATAACTGGAAAGAGATCCCATATTTCAAGAAATTGGAGAAACTAATTGGCGTTCCAGTTATCAATGTTCACATATGGTTTGACAGAAAGCTGAAGAACACATATGATCATCTACTTTTTAGCAGGAGTCCTCTTTTAAGTGTCTATGCTGACATGTCCGTAACATGTAAGGAATATTATAATCCAAACCAGTCTATGCTGGAGTTGGTTTTTGCACCAGCAGAAGAATGGATTTCATGCAGTGATTCAGAAATTATTGATGCTACACTCAAAGAACTTGCAAAACTCTTTCCTGATGAGATAGCTGCAGATCAGAGCAAAGCAAAGATTTTGAAGTACCATGTTGTGAAAACACCAAGGTCGGTTTACAAAACTGTACCAGATTGTGAACCTTGCCGTCCCTTGCAAAGATCTCCCCTAGAGGGTTTCTATTTAGCTGGTGATTACACAAAACAAAAGTATTTAGCCTCAATGGAAGGTGCTGTTCTGTCAGGGAAACTTTGTGCACAAGCAATTGTACAGGATTACGAATTGCTTGTTGCTCGGGGACAAACAAGGTTGGCTGAGGCAAGCGTTCGGTGA

***>Prunus dulcis***

ATGTCTCAGTGGGCTTGTGTCTCTGCTGCTAACTTGAGCTGCCAAGCTAGCATCATCAACACTCAAAAGCTACGAAACACTCCCAGATGCGATGCCTTTTCATTTAAAGGTAGTGAGTTTATGGCTCAAAGCTGTAGATTTTTAAGCCCACAAGCTATTTATGGAAGGCCGAGGAATGGTGCTTGCCCTTTGAAGGTGGTTTGCGTTGATTATCCAAGACCAGACCTTGACAATACTGCTAATTTCTTAGAAGCTGCATATTTCTCTTCCACTTTCCGAGCCTCTCCTCGTCCAGCTAAGCCGTTGAAGGTCGTGATTGCTGGTGCAGGTTTGGCTGGTCTGGCAACTGCAAAATATTTGGCTGATGCAGGTCATAAACCTATCTTACTGGAAGCAAGAGATGTTCTAGGCGGAAAGGTGGCAGCATGGAAAGATAAGGATGGAGACTGGTACGAAACAGGCCTACATATCTTCTTTGGGGCTTATCCGAATATTCAGAACCTGTTTGGTGAGCTTGGTATTGATGATCGATTGCAGTGGAAGGAGCATTCTATGATATTTGCAATGCCAAGCAAACCAGGAGAGTTCAGCCGGTTTGATTTCCCTGAAGTTTTACCAGCACCCTTAAATGGAATATGGGCCATATTGAAGAACAATGAGATGCTGACTTGGCCAGAGAAAATCAAGTTTGCAATTGGACTACTGCCAGCAATTCTTGGTGGGCAGGCTTATGTTGAAGCCCAAGATGGCTTGAGTGTAAAAGATTGGATGAGGAAACAGGGCATACCGGATCGAGTGACTACTGAGGTGTTTATTGCCATGTCAAAGGCCCTGAACTTTATTAACCCTGATGAACTTTCAATGCAGTGCATATTGATTGCTTTGAACCGATTCCTTCAGGAGAAACACGGTTCCAAGATGGCTTTTTTGGATGGTAGTCCCCCTGAGAGACTCTGTGCACCAATTGTTGATCATATCCAGTCATTAGGCGGTGAAGTCCGAATTAATTCCCGCATACAGAGAATTGAGCTAAATAAAGATGGGACCGTGAAGAGTTTTGTACTAAATAATGGGAGCATGATTGAAGCAGATGCCTATGTATTCGCCACTCCAGTTGATATCCTAAAGCTTCTATTGCCTGATAACTGGAAAGAGATCCCATATTTCAAGAAATTGGAGAAACTGGTTGGCGTTCCAGTTATCAATGTTCACATATGGTTTGACAGAAAGCTGAAGAACACATATGATCATCTACTTTTTAGCAGAAGTCCTCTTTTAAGTGTCTATGCCGACATGTCCGTAACATGTAAGGAATATTACAATCCAAACCAGTCTATGCTGGAGTTGGTTTTTGCACCAGCAGAAGAATGGATATCATGCAGTGATTCAGAAATTATTGATGCTACACTCAAAGAACTTGCAAAACTCTTTCCTGATGAGATAGCTGCAGATCAAAGCAAAGCAAAGATTTTGAAGTACCATGTTGTGAAAACACCAAGGTCGGTTTACAAAACTGTACCAGGTTGTGAACCTTGCCGTCCCTTGCAAAGATCTCCCCTAGAGGGTTTCTATTTAGCTGGTGATTACACAAAACAAAAGTATTTAGCCTCAATGGAAGGTGCTGTTCTGTCAGGGAAACTTTGTGCACAAGCAATTGTACAGGATTACGAATTGCTTGTTGCTCGGGGACAAACAAGGGTGGCTGAGGCAAGCGTTCGGTGA

***>Viola philippica***

ATGAGTGTACACGGGAGTGTTTCTGCGTTGAGCTTGACCGGCCATGGTAGCACCTTAAACGTTAGAAACTCACAACCTGGGTTGAGATACCGCCATCCTGCTTGTTTTAGGCAAAGCAGTACACTTGCTTTCACCAGGAGTGAATCAATGGGTCATGCTTTCAAATTCTCAGCTGGAAATGCTCCTTGTAGCAGAGCAAGAAGTAATGTTGGCCGTCTGCGGGTAGTATGTGTGGACTATCCAAGGCCTGAGATTGATAACACCACAAACTTCTTGGAAGCTGCCTTCTTGTCATCAACATTTCGCACTTCTCCACGTCCAGCTAAACCCTTGAAAGTTGTAATTGCTGGTGCAGGTCTGGCTGGTTTATCAACTGCAAAATATTTGGCAGATGCAGGCCACAAGCCTCTATTACTGGAAGCAAGAGATGTTCTAGGTGGAAAGGTGGCTGCTTGGAAAGATGACGATGGAGACTGGTACGAGACTGGATTGCATATATTCTTTGGAGCGTACCCAAATATTCAGAACTTGTTTGGAGAGCTTGGCATTAATGATAGGTTGCAGTGGAAGGAGCATTCCATGATATTTGCAATGCCAAACAAACCAGGAGAGTACAGCCGATTCGATTTCCCCGATGCTCTTCCCGCACCAATCAATGGGATATTGGCCATTTTGAAGAACAATGAAATGCTGACCTGGCCAGAGAAAGTGAAGTTTGCAATTGGACTCCTTCCAGCAATGCTTGGTGGACAGGCTTATGTTGAGGCTCAAGATGGTCTAAGTGTTCAAGAGTGGATGAGAAAGCAGGGGGCACCTGATCGAGTTACTACCGAGGTGTTTATTGCTATGTCAAAGGCATTAAACTTCATTAACCCAGATGAACTGTCAATGCAGTGTATATTGATAGCTTTGAACCGGTTTCTTCAGGAGAAACATGGTTCAAAGATGGCTTTTTTAGATGGTAATCCACCAGAGAGACTCTGCATGCCAATTGTTGATCATATTCAGTCACTTGGTGGTGAAGTCCGGCTGAATTCCCGCATAAAGAAAATTGAGCTAAATGATGATGGTACAGTGAAGAACTTTTTATTAAATAGTGGGGACGTGATTGAAGGAGATGTTTATGTATTTGCTACTCCAGTTGATATCCTGAAGCTTCTTTTGCCTGATAACTGGAAGGAGATTCCTTACTTCAAGAAATTGGAGAAATTAGTTGGAGTTCCCGTTATTAATGTTCACATATGGTTTGACAGGAAACTGAAGAATACATATGATAGCCTACTTTTTAGCAGAAGCCCCCTTCTTAGTGTATATGCTGACATGTCGGTAACATGTAAGGAATATTACGACCCAAATAAATCTATGCTGGAATTAGTTTTTGCACCTGCAGAAGAATGGATCTCACGGACTGATTCCGAGATTATTGATGCTACAATGAAAGAACTTGCAAAACTCTTTCCTGATGAAATAGCCGCTGATCAAAGCAAAGCAAAAATTGTCAAGTACCATGTTGTGAAAACTCCAAGGTCTGTTTACAAGACTGTCCCGAATTGCGAACCTTGTCGGCCTTTACAAAGATCACCTATGGAGGGCTTCTATTTATCTGGTGACTACACGAAACAAAAATATTTGGCATCAATGGAAGGCGCCGTTCTATCGGGGAAGCTGTGTGCACAAGCAATTATGCAGGATTACGAGTTACTTGCTGGTCTTGGGCAGAGAACGCTGGCGGAGGCAACCATTAGTTAG

***>Prunus persica***

ATGTCTCAGTGGGCTTGTGTCTCTGCTGCTAACTTGAGCTGTCAAGCTAGCATCATCAACACTCAAAAGCTACGAAACACTCCCAGATGCGATGCCTTTTCATTTAAAGGTAGTGAATTTATGGCTCAAAGCTGTAGATTTTTAAGCCCACAAACTATTTATGGAAGGCCGAGGAATGGTGCTTGCCCTTTGAAGGTGGTTTGCGTTGATTATCCAAGACCAGACCTTGACAATACTGCTAATTTCTTAGAAGCTGCATATTTCTCTTCCACTTTCCGAGCCTCTCCTCGTCCAGCTAAGCCGTTGAAGGTCGTGATTGCTGGTGCAGGTTTGGCTGGTCTGGCAACTGCAAAATATTTGGCTGATGCAGGTCATAAACCTATCTTACTGGAAGCAAGAGATGTTCTGGGCGGAAAGGTGGCAGCATGGAAAGATAAGGATGGAGACTGGTACGAAACAGGCCTACATATCTTCTTTGGGGCTTATCCGAATATTCAGAACCTGTTTGGTGAGCTTGGTATTGATGATCGATTGCAGTGGAAGGAGCATTCTATGATATTTGCAATGCCAAGCAAACCAGGAGAGTTCAGCCGGTTTGATTTCCCTGAAGTTTTACCAGCACCCTTAAATGGAATATGGGCCATATTGAAGAACAATGAGATGCTGACTTGGCCAGAGAAAATCAAGTTTGCAATTGGACTACTGCCAGCAATTCTTGGTGGGCAGGCTTATGTTGAAGCCCAAGATGGCTTGAGTGTAAAAGATTGGATGAGGAAACAGGGCATACCGGATCGAGTGACTACTGAGGTGTTTATTGCCATGTCAAAGGCCCTGAACTTTATTAACCCTGATGAACTTTCAATGCAATGCATATTGATTGCTTTGAACCGATTCCTTCAGGAGAAACACGGTTCCAAGATGGCTTTTTTGGATGGTAGTCCCCCTGAGAGACTCTGTGCACCAATTGTTGATCATATCCAGTCATTAGGCGGTGAAGTCCGAATTAATTCCCGAATACAGAGAATTGAGCTAAATAAAGATGGGACCGTGAAGAGTTTTGTACTAAATAATGGGAGCATGATTGAAGCAGATGCCTATGTATTCGCCACTCCAGTTGATATCCTAAAGCTTCTATTGCCTGATAACTGGAAAGAGATCCCATATTTCAAGAAATTGGAGAAACTGGTTGGCGTTCCAGTTATCAATGTTCACATATGGTTTGACAGAAAGCTGAAGAACACATATGATCATCTACTTTTTAGCAGAAGTCCTCTTTTAAGTGTCTATGCCGACATGTCCGTAACATGTAAGGAATATTACAATCCAAACCAGTCAATGCTGGAGTTGGTTTTTGCACCAGCAGAAGAATGGATATCATGCAGTGATTCAGAAATTATTGATGCTACACTCAAAGAACTTGCAAAACTCTTTCCAGATGAGATAGCTGTAGATCAAAGCAAAGCAAAGATTTTGAAGTACCATGTGGTGAAAACACCAAGGTCGGTTTACAAAACTGTACCAGGTTGTGAACCTTGCCGTCCCTTGCAAAGATCTCCCCTAGAGGGTTTCTATTTAGCTGGTGATTACACAAAACAAAAGTATTTAGCCTCAATGGAAGGTGCTGTTCTGTCAGGGAAACTTTGTGCACAAGCAATTGTACAGGATTACGAATTGCTTGTTGCTCGGGGACAAACAAGGGTGGCTGAGGCAAGCGTTCGGTG

***>Acer palmatum***

ATGAGCCTCTGCGGAAGCGTTTCTGCTTTGAGCTTGAGATGTGGGTTTCGTCATGGTAATTCGGATATGATGAATGCTATGTCGTTTCGAGGGAGTGAATCCATGGGTCATCCTCTCACAATTCCAACTAAAACCAGACCCAGAAAGGCTTCTCACCCTTTGCAGGTAGTTTGCCTGGACTATCCAAGACCAGAGCTTGAGACTACCGTTAATTTCTTGGAAGATTCTTACTTGTCTTCGTCTTTTCGTGTTTCTCCTCGACCAACTAAGCCATTGAAGATTATAATTGCCGGTGCAGGTTTGGCTGGTTTATCAACTGCTAAATATTTGGCAGATGCAGGCCACAAACCTTTGTTATTGGAAGCAAGAGACGTTCTAGGTGGAAAGGTGGCTGCCTGGAAAGATGAGGATGGAGACTGGTATGAGACGGGCCTCCATATATTTTTTGGGGCTTATCCTAATGTACAGAACCTGTTTGGAGAACTTGGTATCAATGACCGGTTGCAGTGGAAAGAGCACTCTATGATATTTGCCATGCCTAACAAGCCAGGAGAGTTCAGCCGATTTGATTTTCCTGAAGTTCTTCCTGCACCTATAAATGGTATATGGGCCATTTTAAAGAATAATGAAATGCTGACTTGGGCAGAGAAAGTTAAGTTTGCAATTGGTCTGCTTCCAGCAATTCTTGGTGGGCAGGCTTATGTTGAAGCTCAAGATGGTATAACTGTTAAGGAGTGGATGAGAAAGCAGGGCATACCCGACCGAGTGACTACTGAGGTGTTTATTGCCATGTCAAAGGCACTAAACTTCATTAACCCTGACGAACTATCGATGCAATGTATATTGATTGCTTTAAATCGATTTCTTCAGGAGAAGCATGGTTCCAAGATGGCATTCTTAGATGGTAATCCTCCAGAGAGACTCTGCATGCCTATTGTTGATCACATTCGGTCACTGGGTGGTGAAGTCGAACTTAATTCACGAATACAGAAAATTGAACTAAATAGTGATGCAACTGTGAAGAATTTCTTACTAACTAACGGGGAGGTAATTGAAGGAGATGTTTATGTATTTGCCACTCCAGTTGATATCCTTAAGCTTCTTTTACCTGAAAGCTGGAAAGAGATTCCTTACTTCAAGAAATTGGAGAAATTGGTTGGAGTTCCAGTTATTAATGTTCACATATGGTTTGACAGGAAATTGAAAAACACGTCTGATCATCTACTATTTAGCAGAAGTCCCCTCCTAAGTGTGTATGCAGACATGTCGGTAACATGCAAGGAATATTACAACCCTAACCAGTCCATGCTGGAGTTAGTTTTCGCCCCTGCTGAAGAATGGATTTCTTGCAGTGACTCAGAAATCATTGATGCTACAATGAAGGAGCTTGCAAAATTGTTTCCTGATGAAATTTCTGCTGATCAGAGCAAAGCAAAAATACTGAAGTACCATATTGTTAAAACACCAAGGTCTGTATACAAAACTGTTCCAAATTGTGAACCTTGTCGTCCCTTGCAAAGATCTCCTGTAGAGGGATTCTATTTAGCTGGAGATTACACAAAACAGAAGTACCTAGCATCGATGGAAGGGGCTGTTTTGTCGGGGAAGTTTTGTGCACAAGCTATTGTACAGGACTATGAACTGCTTGGTGCACGGGGCGAAGGGAGATTGGCTGAAGCAAGTATTCGGTAA

***>Herrania umbratical***

ATGAGTCTCTGTGGGAGTGTTTCGGCTGTGTACTTGAACTCCCAAAGCAACACAATAAGCATGGGAAGTGTCTTAGCTTTTAGAAGTGGTGAATCCATGGGACATGCCTTGAGAATTCCCGTTAAAAAGAGGTCAAGTAAGGGTGCTTGTCCTTTGCAGGTAGTTTGCATAGATTATCCAAGGCCAGAGCTTGAGAATACTGTTAATTTTTTGGAGGCTGCTTCTCTATCTGCTTCTTTTCGTTCTGCTCCCCGTCCAACTAAGCCATTGAAAGTTATAATTGCTGGTGCAGGTTTGGCTGGTTTGTCAACTGCAAAATATTTAGCAGATGCAGGTCACAAACCTCTGTTGCTTGAAGCAAGAGATGTCCTAGGTGGAAAGGTGGGCGCATGGAAAGATGATGATGGAGATTGGTATGAGACAGGCTTACATATATTCTTCGGGGCTTATCCAAATGTGCAAAGCCTGTTTGGAGAACTTGGCATTAATGACCGGCTGCAATGGAAGGAGCACTCTATGATATTTGCAATGCCAAACAAACCTGGAGAGTTCAGCCGATTTGATTTTCCAGAAGTTCTACCTGCACCCTTAAATGGGATATGGGCCATTTTGAAGAACAATGAAATGCTGACTTGGCCAGAGAAAGTGAAGTTTGCAATAGGACTCCTACCAGCAATGCTTGGTGGACAACCTTACGTTGAGGCCCAAGATGGTCTAACTGTTAAAGAGTGGATGAGAAAGCAGGGCATACCGGATCGTGTGACTAACGAGGTGTTTATTGCCATGTCAAAGGCACTGAACTTCATTAACCCAGATGAACTTTCAATGCAGTGTATATTGATTGCTTTGAATCGATTTCTGCAGGAGAAAAATGGATCAAAGATGGCATTCTTGGATGGCAACCCCCCTGAGAGGCTTTGCATGCCTATTGTTAATCATATTGAGTCACTGGGTGGTGAGGTTCGGCTTAACTCACGAATAAAGAAAATAGAGCTCAATGATGATGGAACTGTGAAGAGTTTTCTTCTAACTAATGGCAATGCAATTGAAGGAGATGCTTATGTAATGGCAGCTCCAGTTGATATCCTGAAGCTACTTTTGCCTGAAGACTGGAGAGAGATTTCATACTTCAAGAAATTAGAGAAATTAGTAGGAGTCCCAGTTATCAACGTTCACATATGGTTTGATAGGAAACTGAAGAACACCTACGATCATCTACTCTTTAGCAGAAGTCCCCTTCTAAGTGTTTATGCTGACATGTCTGTAACGTGTAAGGAATATTACAATCCAAACCAGTCCATGTTGGAGTTAGTTTTTGCCCCTGCAGAAGAATGGATTGCACGTAGTGACTCGGAAATTATTGATGCTACAATGAAGGAGCTTGCAAAACTCTTTCCTGATGAAATTTCTGCAGATCAGAGCAAAGCAAAAGTTATAAAGTACCATATTGTTAAAACACCAAGATCAGTATATAAAACTGTTCCAGATTGTGAACCCTGCCGCCCATTGCAAAGATCTCCGATAGAGGGGTTCTATCTAGCTGGTGATTACACAAAACAAAAGTATCTGGCTTCAATGGAAGGCGCTGTTCTCTCAGGGAAGCTTTGTGCGCAGTCTATTGTACAGGATTATGAGTTGCTTCTTGCTCTGGGACAAAGAAAGTTGGCAGGAGCAAGCATTCACTAG

***>Morus notabilis***

ATGTCTCAGTGGGGTTGTGTTTCCGCGGCCAACTTGGGCTGGCAAAAAACAAGCGCCGCCGTGGATGGTCGGAACGGAGGGAACATGCCCAGATGCTGTTTCTATCTGGGTTCGCAGAAGATGGACTCTTTGGCTTTTGGGTATGGAGAATTTTTGGCTCGCGGGTCGAGAATTTCGTCTTCTCGGGCTGTTGGTAGAAGACAGAAGAAGGGTGTTTCGTTTTCGCCATTGAAGGTAGTTTGTGTGGATTATCCAAGGCCCGAGCTGGACAACACTGTTAACTTCTTAGAAGCTGCTTCCTTGTCTGCCTCTTTTCGTAGCTCTCCTCGTCCGGCTAAACCTTTGAAAGTCGTGATTGCTGGTGCAGGTTTGGCCGGTTTATGTACTGCAAAATACTTGGCAGATGCAGGTCATAAACCTCTATTACTGGAAGCCAGAGATGTTTTAGGTGGAAAGGTGGCAGCATGGAAAGACGATGATGGAGATTGGTATGAGACAGGGTTACATATATTCTTTGGAGCTTACCCAAATTTGCAGAACTTGTTTGGAGAGCTTGGAATTGATGATCGGTTACAATGGAAAGAGCATTCTATGATATTTGCAATGCCTAACAAACCTGGAGAGTTCAGCCGATTTGATTTCCCTGAAGTGCTGCCAGCACCCTTAAATGGAATATGGGCCATCTTAAGGAACAATGAGATGCTGACATGGCCAGAGAAAGTCAAGTTTGCAATCGGACTGCTGCCAGCAATACTTGGTGGCCAGCCTTATGTTGAAGCACAAGATGGTTTAACCGTTAAAGAATGGATGATAAAACAGGGCATACCTGATCGCGTAACTGATGAGGTGTTTATTGCCATGTCAAAGGCCCTAAACTTTATCAACCCTGATGAACTTTCAATGCAGTGTATATTGATTGCGTTAAACCGTTTTCTTCAGGAGAAGCATGGTTCCAAGATGGCCTTCTTGGATGGGAATCCACCAGAGAGACTCTGTATGCCGATAGTTGAGCATATCCAGTCATTGGGTGGTGAAGTCGAGCTTAATTCACGGATACAAAAGATTGACCTAAATGATGATGGAACAGTAAAGAGATTCTTACTAACTAATGGAAGTGCGATAGAAGGGGATGTATACGTTTTTGCGACTCCAGTTGATATCCTAAAGCTTTTATTGCCCGACAACTGGAAAGAGATTCCATATTTCAAGAAATTGGAGAAATTAGTTGGAGTTCCCGTTATCAATGTTCACATATGGTTTGACAGAAAATTGAAGAACACATATGATCACCTGCTTTTCAGCAGAAGTCCTCTCCTAAGTGTCTATGCCGATATGTCAGTAACGTGTAAGGAATATTACAGTCCAAACCAGTCTATGCTGGAGTTAGTTTTTGCACCAGCTGAAGAATGGATTTCATGTAGTGACTCAGAAATTATTGATGCTACAATGAAGGAACTTGCTAAGCTCTTTCCTGATGAAATAGCGGCAGATCAGAGTAAAGCAAAAATTTTGAAATACCATGTTGTAAAAACACCAAGGTCTGTTTACAAAACTGTTCCAGATTGTGAACCTTGTCGTCCGTTGCAAAGATCTCCTATAGAAGGCTTCTATTTAGCAGGCGACTACACAAAACAGAAGTATTTGGCTTCAATGGAGGGTGCTGTTCTCTCAGGGAAGTTTTGTGCACAGTCAATTGTACAGGATTATGAGTTGCTTGCTGCTCGTGGTCAAAGAAGTTTGGCGAAGGCTGGAAGTTGGTAA

***>Gossypium hirsutum***

ATGAGTCTCTGTGGGAGTGTTTCTGCCCTGTACTTAAACTTACAAAGCAGCAAGATAAGCATGGGAAATGTCTTAGCTTTTAGAAGTGGTGAATCCATGGGAAATACCTTGAGAATTCCCTTTAAAAAGAGGTCACGTAAGGGTGCTGGTTGTCCTTTGCAGGTAGTTTGCATAGATTATCCAAGGCCAGAGCTAGAGAATACTGTTAATTTTTTGGAGGCTGCTTCTCTATCTGCTTCTTTTCGTTCAGCTTCCCGTCCAACTAAACCATTGAAAGTCATAATTGCTGGTGCAGGTTTGGCTGGTTTGTCAACTGCAAAGTATCTTGCGGATGCAGGTCATACACCAATATTATTAGAAGCGAGAGATGTTCTAGGTGGAAAGGTGGCTGCATGGAAGGATGATGATGGAGATTGGTATGAGACAGGATTACATATATTCTTTGGGGCTTACCCAAATGTGCAAAACTTGTTTGGAGAACTTGGCATTAATGATCGGCTGCAATGGAAGGAGCATTCTATGATATTTGCGATGCCAAATAAACCTGGAGAGTTCAGTCGATTTGATTTTCCAGAAGTTCTACCTGCACCATTAAATGGAATATGGGCCATTTTGAAGAATAATGAAATGCTGACTTGGCCAGAGAAAGTGAAATTTGCAATAGGACTCCTACCAGCAATGCTGGGTGGACAACCTTATGTTGAGGCCCAAGATGGTTTATCTGTTAAAGACTGGATGAGAAAGCAGGGCGTACCTGATCGTGTGACTGAGGAGGTGTTTATTGCCATGTCAAAGGCACTGAACTTCATTAACCCTGATGAACTTTCAATGCAATGTATATTGATTGCATTGAATCGATTTCTTCAGGAGAAACATGGATCAAAGATGGCATTCTTGGATGGAAACCCTCCCGAGAGGCTTTGCATGCCTATCGTCAATCATATTGAATCACTGGGGGGTGAGGTCCGGCTTAATTCACGTATAAAAAAAATAGAGCTCAATGAAGATGGAACTGTGAAGACTTTTCTTCTAAATAATGGCAATACAATCGAAGGAGATGCTTATGTAGTTGCAACGCCCGTTGATATCTTCAAGTTACTTTTGCCTGAAGACTGGAGAGAGATTTCATACTTCAAGAAATTAGAGAAATTAGTTGGAGTTCCAGTTATCAACGTTCACATCTGGTTTGATAGGAAATTGAAGAACACCTATGATCATCTACTCTTTAGCAGAAGCCCGCTTTTAAGTGTTTATGCTGACATGTCTGTAACATGTAAGGAATATTACAATCCAAACCAATCCATGTTGGAGTTAGTTTTTGCCCCAGCAGAAGAATGGATTGCATGTAGTGACTCAGAAATTATTGATGCTACAATGAAGGAACTTGCAAAGCTCTTTCCTGATGAAATATCTGCAGATCAGAGTAAAGCAAAAGTCGTAAAATACCATATTGTTAAAACACCAAGATCTGTATATAAAACTGTTCCAAATTGTGAACCCTGCCGCCCCTTGCAAAGATCTCCAATACAGGGGTTCTATCTAGCAGGTGATTACACAAAGCAAAAGTATTTAGCTTCGATGGAAGGTGCTGTGCTCTCAGGGAAGCTTTGTGCACAGTCTATTGTACAGGATTATGAGTTGCTTTGTACTTTGGGACAAAGAAAGTTGACAGGAGCAAGCATTCACTGA

***>Carica papaya***

ATGACTTTATGCGGGAGTGTTTCTGCGGCGAGCTTCGGCTGCCAAAGCAACAGAATAGCTATTGGAAACCTTCATTCTGCCGCCTCGAAATGTGGTTATCGAGACACTCTGGATCAAAACAACATACTAGCATTTAGGGTTAGTGAATCCATTGGAGACGGCCTGAGAATTCCCGAAGCACGAGCTGTTAAGATTAGGTCCAGGAACGGTGCCCGCCCTTTGCAGGTAGTTTGTGTAGATTACCCGAGACCAGAGCTTGATAATACTTTAAATTTCTTGGAAGCAGCGTACTTGTCTTCATCCTTTCGGACTTCTCCCCGTCCATCGAGACCATTGAAGATCGTAATTGCTGGTGCAGGTTTGGCTGGTTTATCGACTGCAAAATATTTGGCAGATGCAGGTCACAAGCCTTTGTTGCTGGAAGCAAGAGATGTTCTAGGTGGAAAGGTGGCTGCATGGAAAGATGATGATGGAGACTGGTATGAGACAGGCTTACATATATTCTTTGGGGCCTATCCAAATATGCAGAACTTGTTTGGAGAACTTGGTATCAATGATCGGTTGCAGTGGAAGGAGCACTCAATGATATTTGCAATGCCAAACAAGCCTGGGGAATTCAGCCGATTTGACTTCCTTGAAGAATTGCCGGCACCTTTAAACGGAATTTGGGCAATTTTAAAAAATAATGAAATGCTGACTTGGCCTGAGAAAGTGAAGTTTGCGATTGGGCTTCTGCCTGCAATGGTCGGTGGACAGGAGTATGTTGAGGCTCAAGATGGTTTAAGTGTTCAAGAGTGGATGAGAAAGCAGGGCATACCTGACCGGGTGACTAATGAGGTGTTTATCGCTATGTCAAAGGCACTAAACTTCATTAACCCAGATGAACTGTCAATGCAATGTATACTGATTGCTTTGAACCGATTTCTTCAGGAGAAGCATGGTTCTAAGATGGCATTCTTAGATGGTAACCCTCCAGAAAGACTCTGTATGCCAATTGTTGATCATATCCTGTCACTAGGTGGTGAAGTGAAACTTAATTCTCGGATACAGACAATTGAGCTCAACAATGATGGAACTGTGAAGAGCTTTATACTAAATAGTGGGGATGTGATTGAAGGAGATGCTTATGTATTTGCCACTCCAGTTGATATCTTGAAGCTTCTTCTGCCTGAAAGCTGGAAAGAGATCCTATACTTCAAGAGATTGGAGAAATTAGTTGGCGTCCCTGTTATTAATGTTCACATATGGTTTGACAGGAAACTAAAGAACACATATGACCACCTACTCTTCAGCAGAAGTCCCCTCCTGAGTGTATATGCTGATATGTCTGTAACATGTAAGGAATATTACAACCCAAATCAATCCATGCTGGAGTTAGTTTTTGCCCCTGCTGAAGAATGGATTTCACGCAGTGATTCAGAAATTATTGATGCTACAATGAAGGAACTTGCAAAACTTTTTCCTGATGAAATTGCAGCGGACCAAGGCAAAGCAAAAATATTGAAATACCATGTTGTTAAAACACCAAGGTCTGTCTACAAAACTGTCCCAGGTTGTGAACCTTGCCGCCCGGTACAGAGAACTCCTATAGAGGGTTTTTACTTAGCTGGTGATTATACTAAACAAAAGTATTTGGCTTCAATGGAAGGAGCTGTTCTCTCAGGGAAGCTTTGTGCACAGGCTATTGTACAGGATTATGAGTTTCTTTTTGCTTCAGCGCAAAGAAGGTTGGCACAGGCAAGCATTCATTGA

***>Theobroma cacao***

ATGAGTCTCTGTGGGAGTGTTTCTGCTGTGCGCTTGAACTCCCAAAGCAACACAATAAGCATGGGAAGTGTCTTAGCTTTTAGAGGTGGTGAATCCATGGGACATGCCTTGAGAATTCCCTTTAAAAAGAGGTCAAGTAAGGGTGCTTGTCCTTTGCAGGTAGTTTGCATAGATTATCCAAGGCCAGAGCTTGAGAATACTGTTAATTTTTTGGAGGCTGCTTCTCTATCTGCTTCTTTTCGTTCTGCTCCCCGTCCAACTAAGCCATTGAAAGTTATAATTGCTGGTGCAGGTTTGGCTGGTTTGTCAACTGCAAAATATTTAGCAGATGCAGGTCACAAACCTCTGTTGCTTGAAGCAAGAGATGTCCTAGGTGGAAAGGTGGCCGCATGGAAAGATGATGATGGAGATTGGTATGAGACAGGCCTACATATATTCTTCGGGGCTTATCCAAATGTGCAAAACCTGTTTGGAGAACTTGGCATTAATGACCGGCTGCAATGGAAGGAGCACTCTATGATATTTGCAATGCCAAACAAACCTGGAGAGTTCAGCCGATTTGATTTTCCAGAAGTTCTACCTGCACCCTTAAATGGGATATGGGCCATTTTGAAGAACAATGAAATGCTGACTTGGCCAGAGAAAGTGAAGTTTGCAATAGGACTCCTACCAGCAATGCTTGGCGGACAACCTTATGTTGAGGCCCAAGATGGTCTAACTGTTAAAGAGTGGATGAGAAAGCAGGGCATACCTGATCGTGTGACTGACAATGTGTTTATTGCCATGTCAAAGGCACTGAACTTCATTAACCCAGATGAACTTTCAATGCAGTGTATATTGATTGCTTTGAATCGATTTCTGCAGGAGAAAAATGGATCAAAGATGGCATTCTTGGATGGCAACCCCCCTGAGAGGCTTTGCATGCCTATTGTTAATCATATTGAGTCACTGGGTGGTGAGGTCTGGCTTAACTCACGAATAAAGAAAATAGAGCTCAATGATGATGGAACTGTGAAGAGTTTTCTTCTAACTAATGGCAATACAATTGAAGGAGATGCTTATGTAATGGCAGCTCCAGTTGATATTCTGAAGCTACTTTTGCCTGAAGACTGGAGAGAGATTTCATACTTCAAGAAATTAGAGAAATTAGTTGGAGTCCCAGTAATCAACGTTCACATATGGTTTGATAGGAAATTGAAGAACACCTACAATCATCTACTCTTTAGCAGAAGTCCCCTTCTAAGTGTCTATGCTGACATGTCTGTAACGTGTAAGGAATATTACAATCCAAACCAGTCCATGTTGGAGTTAGTTTTTGCCCCTGCAGAAGAATGGATTGCCCGTAGTGACTTGGAAATTATTGATGCTACAATGAAGGAGCTTGCAAAACTCTTTCCTGATGAAATTTCTGCAGATCAGAGCAAAGCAAAAGTTATAAAGTACCATATTGTTAAAACACCAAGATCTGTATATAAAACTGTTCCAGATTGTGAACCCTGCCGCCCATTGCAAAGATCTCCAATAGAGGGGTTCTATCTAGCAGGTGATTACACAAAACAAAAGTATCTGGCTTCGATGGAAGGCGCTGTTCTCTCAGGGAAGCTTTGTGCACAGTCTATTGTACAGGATTATGAGTTGCTTCTTGCTCTGGGACAAAGAAAGTTGGCAGGAGCAAGCATTCACTAA

***>Abrus precatorius***

ATGGCTGCGTGTGGGTGTATATCTGCGGCGAACTTGAATTGGCAGATTGGTGCTAGAAGCATATCCAAATTCGGTTCTTCAGATGCCACAATTTCGTTATCATTTGGTGGGAGTGAGTCCATGGGTGTTAGTGTGCGACCTCGTTCTGCTAAGAGCACCAGGTTGAGGAACCATGCATCACCCTTGAGTGTCGTTTGTGTCGATTATCCACGCCCTGAGCTTGAAAACACTGTTAATTTCATCGAAGCTGCTTACTTGTCTTCCACCTTTCGTGCTTCTCCGCGTCCAGAAAAACCCTTGAATGTCGTTATTGCTGGTGCAGGATTGGCTGGCTTATCAACTGCAAAATATTTAGCAGATGCTGGTCATAAACCTATATTGCTGGAGGCAAGAGATGTTCTAGGTGGAAAGGTTGCTGCATGGAAAGATGAAGATGGAGACTGGTACGAGACAGGCCTACACATCTTCTTTGGGGCTTACCCTAATGTGCAGAACTTATTTGGAGAACTTGGTATTAATGATCGGTTACAATGGAAGGAGCATTCTATGATTTTTGCAATGCCAAATAAGCCTGGAGAGTTTAGTCGCTTTGATTTTCCTGAAGTTCTTCCCGCCCCATTAAATGGAATATGGGCAATATTGAGGAACAATGAGATGCTGACATGGCCAGAGAAAGTCAAATTTGCAATTGGGCTTCTGCCAGCTATGCTTGGTGGACAGGCGTATGTTGAGGCTCAAGATGGTGTTTCTGTTAAGGAGTGGATGAGAAAGCAGGGCATACCCGATCGGGTAACTGATGAGGTGTTCATAGCAATGTCAAAGGCACTAAACTTCATCAATCCTGATGAACTTTCAATGCAATGTATATTGATTGCTTTAAACCGATTTCTTCAGGAGAAACATGGTTCTAAGATGGCCTTTTTGGATGGCAATCCCCCTGAAAGACTTTGTATACCAATTGTTGATCATATTCAGTCCTTGGGGGGTGAAGTTCATCTGAATTCACGCATTCAAAAAATTGAGCTAAATGATGATGGCACAGTGAAGAGCTTCTTACTAAATAATGGGAGGGTGATGGAAGGGGATGCTTACGTGTTTGCAACTCCAGTGGATATTCTGAAGCTTCTTCTGCCTGACAACTGGAAAGGAATTCCTTATTTTCAGAGATTGGATAAATTAGTTGGAGTCCCGGTCATAAATGTTCACATATGGTTTGACAGAAAACTGAAAAACACATATGATCACCTTCTCTTTAGCAGAAGTCCCCTTCTGAGTGTATATGCTGACATGTCAGTTACTTGCAAGGAATATTATAACCCAAACCAGTCTATGTTGGAGTTAGTTTTTGCACCAGCTGAAGAATGGGTGTCACGTAGTGATGAAGATATTATACGTGCCACAATGTCTGAACTTGCCAAACTCTTTCCTAATGAAATTTCTGCTGATCAAAGCAAAGCGAAGATTGTCAAGTACCATGTTGTTAAAACACCAAGGTCGGTTTACAAAACTGTTCCAAATTGTGAACCTTGTCGTCCCGTACAAAGATCTCCTGTAGAAGGTTTCTATTTAGCTGGAGATTACACAAAACAAAAATATTTAGCTTCAATGGAAGGTGCTGTTCTTTCTGGGAAGCTTTGTGCACAGGCAATTGTACAGGATTCTGAGCTACTTGCTGCGCGTGGTCACAAAAGAATGGCTCAAGCAAGTGTTATTTAA

***>Ricinus communis***

ATGGCTCTATATGGGGGTGTTTCTGCTTTGAATTTAAGCTGGCATAGTGATGTCTTAGACACTAGAAATCTGCAATCAGCCCTTAGATGTGGTTACGCTACCTGTTCTAATCAAACCAATGTACTAGCTTTTAGAGGCAGTGAATCTATGGGCCATGCTTTGAGAAATTCTTCTAAAACAAGATTTAGGAATACTGGTAGCTGCCCTTTGAAGGTAGTTTGTGTGGACTATCCTAGACCAGACCTTGATAACACAGTGAATTTCTTGGAAGCTGCCTACTTATCATCATCTTTTCGATCTTCTTCCCCTCCAGATAAACCATTGAAGGTTGTAATTGCTGGTGCAGGATTGGCTGGTTTATCAACTGCAAAATATTTGGCAGATGCAGGACACAAGCCTTTATTGCTGGAAGCAAGAGATGTTCTAGGTGGAAAGGTGGCTGCATGGAAAGATGATGATGGGGACTGGTACGAGACAGGCTTGCATATATTCTTTGGAGCATACCCAAATGTGCAGAACCTGTTTGGAGAACTTGGTATAAATGATAGATTGCAGTGGAAGGAGCATTCTATGATATTTGCGATGCCAAACAAGCCTGGAGAATTCAGCCGATTTGATTTCCCAGATGTTCTTCCTGCACCATTAAATGGGATATGGGCAATTCTGAGAAACAATGAGATGCTGACATGGCCAGAGAAAGTGAAATTTGCAATTGGACTCCTGCCAGCGATGGTTGGTGGACAGGCCTATGTTGAGGCTCAAGATGGTTTAAGTGTTCAAGAGTGGATGAGAAAGCAGGGCGTACCTGATAGAGTGACTAAGGAGGTTTTTATTGCTATGTCAAAGGCGCTAAACTTTATTAACCCTGATGAGCTTTCAATGCAATGTATATTGATAGCATTGAACAGATTTCTTCAGGAGAAACATGGTTCAAAGATGGCTTTCTTAGATGGAAATCCCCCAGAGAGACTCTGCATGCCAATTGTTGACCATGTGCAGTCACTTGGTGGTGAAGTCCGGCTAAATTCACGAATAAAGAAAATTGAATTAAATAATGATGGAGCAGTGAAGAACTTTTTACTAAATAATGGGGAAGTGATTGAAGGAGATGTTTATGTGGTTGCTACTCCAGTTGATATCCTGAAGCTTCTTTTGCCTGATAACTGGAAAGAGATTCCATACTTCAAGAAGCTGGATAAATTAGTTGGAGTTCCTGTTATTAATGTTCACATATGGTTTGACAGGAAGCTGAAGAATACATATGATCACCTACTTTTCAGCAGAAGTCCCCTTCTTAGTGTTTATGCGGACATGTCTGTAACATGTAAGGAATATTATAATCCAAATCAGTCTATGCTGGAGTTAGTTTTTGCACCTGCAGAAGAATGGGTATCACGCAGCGATGAAGAAATTATTGAGGCTACAATGATGGAACTAGCAAAACTCTTTCCTGATGAAATATCTGCAGATCAGAGCAAAGCAAAAATTGTTAAATACCATGTTGTCAAAACTCCCAGGTCTGTTTACAAGACTGTCCCAAATTGTGAACCTTGCCGACCCTTGCAAAGATCTCCTATAGAGGGCTTCTATTTGGCTGGTGACTACACAAAACAAAAATATTTGGCTTCGATGGAGGGTGCTGTTCTATCCGGGAAGTATTGTGCACAAGCCATTGTACAGGATTATGGGTTGCTTATCGCTCGCAAGCAAAAAAAGTTGGCTGAGGTAACCGTAATTTAA

***>Citrus x paradisi***

ATGAGCCTTTGCTTCAGCGTTTCTGAAAGTGCTTTCAACTTGCGATATGGTTTCCGAGATAGTGAACCGATGGGTCAGAGCCTGAAAATTCGAGTTAAAACGAGGACAAGGAAGGGTTTCTGTCCTTCGAAGGTGGTTTGTGTGGACTACCCAAGACCAGATATTGATAATACATCTAATTTCTTGGAAGCTGCTTACTTATCTTCGTCATTTCGTACTTCTCCTCGTCCTTCTAAGCCGTTGAAAGTTGTAATTGCTGGTGCAGGTTTGGCTGGTTTATCAACTGCAAAATATTTGGCAGATGCAGGCCACAAGCCTTTGTTACTGGAAGCAAGAGATGTTCTAGGTGGAAAGATAGCTGCCTGGAAAGATGGGGACGGGGACTGGTATGAGACAGGCCTTCATATTTTCTTCGGGGCTTACCCAAATATACAGAACCTGTTTGGAGAACTTGGTATTAATGACCGGTTGCAGTGGAAGGAGCACTCTATGATTTTTGCAATGCCAAACAAGCCCGGAGAATTCAGCCGATTTGATTTTCCTGAAGTTCTTCCAGCTCCGCTAAATGGGATATTGGCCATTTTAAGGAACAATGAAATGCTGACTTGGCCGGAGAAAGTGAAGTTTGCRATTGGACTGCTTCCAGCAATAATTGGTGGACAGGCATATGTTGAAGCTCAAGATGGTTTAACTGTTCAGGAGTGGATGAGAAAGCAGGGTGTACCTGATCGAGTGACGACGGAGGTGTTTATTGCCATGTCAAAGGCACTAAACTTCATAAACCCTGATGAACTGTCAATGCAATGTATATTGATTGCCTTAAACCGATTTCTTCAGGAGAAGCATGGTTCRAAGATGGCATTCTTAGATGGCAACCCCCCAGAGAGACTTTGCTTGCCTATTGTTGAACACATTCAGTCACTGGGTGGTGAAGTCCGGCTTAATTCCCGAGTTCAGAAAATTGAGCTCAATGATGATGGAACTGTGAAGAATTTTTTACTAACTAATGGCAATGTGATTGACGGAGATGCTTATGTATTTGCCACACCTGTTGATATCCTCAAGCTTCAGTTACCTGAAAACTGGAAAGAGATGGCATACTTCAAGAGATTAGAGAAATTGGTGGGAGTTCCAGTCATCAACATCCACATATGGTTTGACAGGAAATTGAAAAACACTTATGATCACCTACTCTTTAGCAGAAGTCCCCTTCTAAGTGTGTATGCCGACATGTCTTTAACTTGTAAGGAGTATTACAACCCCAATCAATCCATGCTGGAGTTAGTTTTTGCCCCGGCTGAAGAGTGGATCTCATGCAGTGACTCAGAAATCATTGATGCTACAATGAAGGAGCTTGCAAAACTATTTCCTGATGAAATTTCTGCTGATCAGAGCAAAGCAAAGATTGTGAAGTACCATGTCGTCAAAACGCCAAGGTCTGTATATAAAACCATCCCAAATTGTGAACCTTGCCGTCCCTTACAAAGGTCTCCTGTAGAAGGGTTTTATTTAGCCGGGGATTACACAAAACAGAAGTATTTRGCTTCAATGGAAGGTGCTGTTTTGTCAGGGAAGCTTTGTGCACAAGCAATTGTACAGGACTATGTGCTGCTTGCTGCACGGGGGAAAGGGAGATTGGCTGAGGCAAGCATGTGTCCATAA

***>Citrus clementina***

ATGAGCCTTTGCTTCAGCGTTTCTGAAAGTGCTTTCAACTTGCGATATGGTTTCCGAGATAGTGAACCGATGGGTCAGAGCCTGAAAATTCGAGTTAAAACGGGGACAAGGAAGGGTTTCTGTCCTTCGAAGGTGGTTTGTGTGGACTACCCAAGACCAGATATTGATAATACATCTAATTTCTTGGAAGCTGCTTACTTGTCTTCGTCATTTCGTACTTCTCCTCGTCCTTCTAAGCCGTTGAAAGTTGTAATTGCTGGTGCAGGTTTGGCTGGTTTATCAACTGCAAAATATTTGGCAGATGCAGGCCACAAGCCTTTGTTACTGGAAGCAAGAGATGTTCTAGGTGGAAAGGTAGCTGCCTGGAAAGATGGGGACGGGAACTGGTATGAGACAGGCCTTCATATTTTCTTCGGGGCTTACCCAAATATACAGAACCTGTTTGGAGAACTTGGTATTAATGATCGGTTGCAGTGGAAGGAGCACTCTATGATTTTTGCAATGCCAAACAAGCCCGGAGAATTCAGCCGATTTGATTTTCCTGAAGTTCTTCCGGCTCCGCTAAATGGGATATTGGCCATTTTAAGGAATAATGAAATGCTGACTTGGCCGGAGAAAGTGAAGTTTGCAATTGGACTGCTTCCAGCAATAATTGGCGGACAGGCATATGTTGAAGCTCAAGATGGTTTAACTGTTCAGGAGTGGATGAGAAAGCAGGGTGTACCTGATCGAGTGACGACAGAGGTGTTTATTGCCATGTCAAAGGCACTAAACTTCATAAACCCTGATGAACTGTCAATGCAATGTATATTGATTGCCTTAAACCGATTTCTTCAGGAGAAGCATGGTTCGAAGATGGCATTCTTAGATGGCAACCCCCCAGAGAGACTTTGCTTGCCTATTGTTGAACACATTCAGTCACTGGGTGGTGAAGTCCGGCTTAATTCCCGAGTTCAGAAAATTGAGCTCAATGATGATGGAACTGTGAAGAATTTTTTACTAACTAATGGCAATGTGATTGACGGAGATGCTTATGTATTTGCCACACCTGTTGATATCCTCAAGCTTCAGTTACCTGAAAACTGGAAAGAGATGGCATACTTCAAGAGATTAGAGAAATTGGTGGGAGTTCCAGTCATCAACATCCACATATGGTTTGACAGGAAATTGAAAAACACTTATGATCACCTACTCTTTAGCAGAAGTTCCCTTCTAAGTGTGTATGCCGACATGTCTTTAACTTGTAAGGAGTATTACAACCCCAATCAATCCATGCTGGAGTTAGTTTTTGCCCCGGCTGAAGAGTGGATCTCATGCAGTGACTCAGAAATCATTGATGCTACAATGAAGGAGCTTGCAAAACTATTTCCTGATGAAATTTCTGCTGATCAGAGCAAAGCAAAGATTGTGAAGTACCATGTCGTCAAAACGCCAAGGTCTGTATATAAAACCATCCCAAATTGTGAACCTTGCCGTCCCTTACAAAGGTCTCCTGTAGAAGGGTTTTATTTAGCCGGGGATTACACAAAACAGAAGTATTTGGCTTCAATGGAAGGTGCTGTTTTGTCAGGGAAGCTTTGTGCACAAGCAATTGTACAGGACTATGTGCTGCTTGCTGCACGGGGGAAAGGGAGATTGGCTGAGGCAAGCATGTGTCCATAA

***>Citrus sinensis***

ATGAGCCTTTGCTTCAGCGTTTCTGAAAGTGCTTTCAACTTGCGATATGGTTTCCGAGATAGTGAACCGATGGGTCAGAGCCTGAAAATTCGAGTTAAAACGGGGACAAGGAAGGGTTTCTGTCCTTCGAAGGTGGTTTGTGTGGACTACCCAAGACCAGATATTGATAATACATCTAATTTCTTGGAAGCTGCTTACTTGTCTTCGTCATTTCGTACTTCTCCTCGTCCTTCTAAGCCGTTGAAAGTTGTAATTGCTGGTGCAGGTTTGGCTGGTTTATCAACTGCAAAATATTTGGCAGATGCAGGCCACAAGCCTTTGTTACTGGAAGCAAGAGATGTTCTAGGTGGAAAGGTAGCTGCCTGGAAAGATGGGGACGGGAACTGGTATGAGACAGGCCTTCATATTTTCTTCGGGGCTTACCCAAATATACAGAACCTGTTTGGAGAACTTGGTATTAATGATCGGTTGCAGTGGAAGGAGCACTCTATGATTTTTGCAATGCCAAACAAGCCCGGAGAATTCAGCCGATTTGATTTTCCTGAAGTTCTTCCGGCTCCGCTAAATGGGATATTGGCCATTTTAAGGAATAATGAAATGCTGACTTGGCCGGAGAAAGTGAAGTTTGCAATTGGACTGCTTCCAGCAATAATTGGCGGACAGGCATATGTTGAAGCTCAAGATGGTTTAACTGTTCAGGAGTGGATGAGAAAGCAGGGTGTACCTGATCGAGTGACGACAGAGGTGTTTATTGCCATGTCAAAGGCACTAAACTTCATAAACCCTGATGAACTGTCAATGCAATGTATATTGATTGCCTTAAACCGATTTCTTCAGGAGAAGCATGGTTCGAAGATGGCATTCTTAGATGGCAACCCCCCAGAGAGACTTTGCTTGCCTATTGTTGAACACATTCAGTCACTGGGTGGTGAAGTCCGGCTTAATTCCCGAGTTCAGAAAATTGAGCTCAATGATGATGGAACTGTGAAGAATTTTTTACTAACTAATGGCAATGTGATTGACGGAGATGCTTATGTATTTGCCACACCTGTTGATATCCTCAAGCTTCAGTTACCTGAAAACTGGAAAGAGATGGCATACTTCAAGAGATTAGAGAAATTGGTGGGAGTTCCAGTCATCAACATCCACATATGGTTTGACAGGAAATTGAAAAACACTTATGATCACCTACTCTTTAGCAGAAGTTCCCTTCTAAGTGTGTATGCCGACATGTCTTTAACTTGTAAGGAGTATTACAACCCCAATCAATCCATGCTGGAGTTAGTTTTTGCCCCGGCTGAAGAGTGGATCTCATGCAGTGACTCAGAAATCATTGATGCTACAATGAAGGAGCTTGCAAAACTATTTCCTGATGAAATTTCTGCTGATCAGAGCAAAGCAAAGATTGTGAAGTACCATGTCGTCAAAACGCCAAGGTCTGTATATAAAACCATCCCAAATTGTGAACCTTGCCGTCCCTTACAAAGGTCTCCTGTAGAAGGGTTTTATTTAGCCGGGGATTACACAAAACAGAAGTATTTGGCTTCAATGGAAGGTGCTGTTTTGTCAGGGAAGCTTTGTGCACAAGCAATTGTACAGGACTATGTGCTGCTTGCTGCACGGGGGAAAGGGAGATTGGCTGAGGCAAGCATGTGTCCATAA

***>Citrus maxima***

ATGAGCCTTTGCTTCAGCGTTTCTGAAAGTGCTTTCAACTTGCGATATGGTTTCCGAGATAGTGAACCGATGGGTCAGAGCCTGAAAATTCGAGTTAAAACGAGGACAAGGAAGGGTTTCTGTCCTTCGAAGGTGGTTTGTGTGGACTACCCAAGACCAGATATTGATAATACATCTAATTTCTTGGAAGCTGCTTACTTATCTTCGTCATTTCGTACTTCTCCTCGTCCTTCTAAGCCGTTGAAAGTTGTAATTGCTGGTGCAGGTTTGGCTGGTTTATCAACTGCAAAATATTTGGCAGATGCAGGCCACAAGCCTTTGTTACTGGAAGCAAGAGATGTTCTAGGTGGAAAGATAGCTGCCTGGAAAGATGGGGACGGGGACTGGTATGAGACAGGCCTTCATATTTTCTTCGGGGCTTACCCAAATATACAGAACCTGTTTGGAGAACTTGGTATTAATGACCGGTTGCAGTGGAAGGAGCACTCTATGATTTTTGTAATGCCAAACAAGCCCGGAGAATTCAGCCGATTTGATTTTCCTGAAGTTCTTCCAGCTCCGCTAAATGGGATATTGGCCATTTTAAGGAACAATGAAATGCTGACTTGGCCGGAGAAAGTGAAGTTTGCAATTGGACTGCTTCCAGCAATAATTGGTGGACAGGCATATGTTGAAGCTCAAGATGGTTTAACTGTTCAGGAGTGGATGAGAAAGCAGGGTGTACCTGATCGAGTGACGACGGAGGTGTTTATTGCCATGTCAAAGGCACTAAACTTCATAAACCCTGATGAACTGTCAATGCAATGTATATTGATTGCCTTAAACCGATTTCTTCAGGAGAAGCATGGTTCGAAGATGGCATTCTTAGATGGCAACCCCCCAGAGAGACTTTGCTTGCCTATTGTTGAACACATTCAGTCACTGGGTGGTGAAGTCCGGCTTAATTCCCGAGTTCAGAAAATTGAGCTCAATGATGATGGAACTGTGAAGAATTTTTTACTAACTAATGGCAATGTGATTGACGGAGATGCTTATGTATTTGCCACACCTGTTGATATCCTCAAGCTTCAGTTACCTGAAAACTGGAAAGAGATGGTATACTTCAAGAGATTAGAGAAATTGGTGGGAGTTCCAGTCATCAACATCCACATATGGTTTGACAGGAAATTGAAAAACACTTATGATCACCTACTCTTTAGCAGAAGTCCCCTTCTAAGTGTGTATGCCGACATGTCTTTAACTTGTAAGGAGTATTACAACCCCAATCAATCCATGCTGGAGTTAGTTTTTGCCCCGGCTGAAGAGTGGATCTCATGCAGTGACTCAGAAATCATTGATGCTACAATGAAGGAGCTTGCAAAACTATTTCCTGATGAAATTTCTGCTGATCAGAGCAAAGCAAAGATTGTGAAGTACCATGTCGTCAAAACGCCAAGGTCTGTATATAAAACCATCCCAAATTGTGAACCTTGCCGTCCCTTACAAAGGTCTCCTGTAGAAGGGTTTTATTTAGCCGGGGATTACACAAAACAGAAGTATTTGGCTTCAATGGAAGGTGCTGTTTTGTCAGGGAAGCTTTGTGCACAAGCAATTGTACAGGACTATGTGCTGCTTGCTGCACGGGGGAAAGGGAGATTGGCTGAGGCAAGCATGTGTTAA

***>Diospyros kaki***

ATGTCTCAATTCGGACATGTTTCTGCCCTCAACCTGAGTGGGCAAAGCAATCTAATAAACTTTTGGAACCCACAATCCACTTGGATATGTGGTTCAAGGCAAACCAATGTACTATCATTTGGAGGGACTGATTCCGTGGGTTATGGGTTGAGAATTCCTAATGCAAATGCTATTAGAACAAGACCGAAGAAGGGCGTGTGCCCCTTGCAGGTCGTCTGCATTGACTATCCAAGACCAGATCTTGACAGCACTTCCAATTTTTTGGAAGCAGCTTATTTGTCTTCATTCTTCCGTACGGCTCCCCGACCAGATAAGCCGCTGAAGGTTGTAATTGCGGGTGCAGGTTTGGCTGGATTATCAACTGCAAAATATTTGGCAGATGCAGGTCATAAACCTTTATTATTGGAAGCGAGGAATGTTTTAGGTGGAAAGGTGGCTGCTTGGAAAGATGAGGATGGAGACTGGTATGAGACTGGATTACATATATTTTTTGGGGCTTACCCAAATGTACAGAACCTGTTTGGAGAGCTTGGTATAAATGATAGGTTGCAGTGGAAAGAACATTCTATGATATTTGCAATGCCAAATAAGCCAGGGGAGTTCAGCCGATTTGACTTCGCTGAAGTTCTACCAGCACCATTAAATGGGATTTGGGCCATCTTAAAGAATAATGAAATGCTTACTTGGCCTGAGAAAGTCAAGTTTGCAATTGGACTGTTGCCAGCAATGATAGGTGGGCAGCCCTATGTCGAAGCTCAAGATGGTTTAACTGTTAAAGACTGGATGAGGAAACAAGGTGTACCAGATCGAGTGACCACTGAGGTGTTCATTGCCATGTCTAAAGCATTAAACTTCATAAACCCTGATGAACTTTCAATGCAGTGTATTTTGATTGCTTTGAACCGGTTTCTTCAGGAGAAACATGGTTCAAAGATGGCATTCTTGGATGGTAATCCCCCTGAGAGACTTTGCCAGCCAATTGTGGATCACATTCAATCACTGGGAGGTGAAGTCCAACTTAATGCTCGAATTCAAAAAATTGAGTTGAATGAAGATGGAACTGTGAAGAGCTTTTTACTAAATAATGGTAATGTCATCAGTGGAGATGCTTATGTGTTTGCAACTCCAGTTGATATCTTGAAGCTTCTTTTGCCGGATGACTGGAAAGGGGTTCCCTACTTCAAAAAATTAGATAAACTAGTTGGAGTTCCTGTTATAAACGTTCACATATGGTTTGACAGGAAGCTGAGGAACACATATGATCATTTACTTTTTAGCAGAAGTCCCCTTCTCAGTGTATATGCTGACATGTCGGTAACATGTAAGGAATATTACAATCCAAATCAGTCTATGCTAGAATTGGTTTTTGCACCAGCAGAGGAATGGATTTCGCGGAGTGACACAGAAATTATTGATGCTACTATGAAGGAACTTGCAAAACTCTTCCCTGATGAAATTTGTCCAGATCAGAGCAAAGCAAAAATTTTGAAGTATCATGTTGTTAAAACACCGAGATCTGTGTATAAAACTGTCCCAAACTGTGAACCATGCCGTCCCTTGCAAAGGTCCCCTATAGAAGGATTCTATTTAGCCGGTGACTACACAAAACAAAAATATTTGGCTTCAATGGAAGGTGCTGTCCTATCAGGAAAGCTTTGTGCCCAAGCTGTTGTACAGGATTACGAGTTTCTTGCAGCCCAGGGGCAGAGAAAGCTGGTGGAAGCAAGTATGGTGTAA

***>Rhododendron kiusianum***

ATGTCTCAATTTGGACATGCTTCTGCTGTATATTGGACTGGGCAACACAACGCAACTAATTTGTGGAACCCAAGGTATACTTGGAGATGCGGTTGTCCCATTAGTTCAAGGCATAACAATGCGCTATCATTTAAAGGGAGTGATTCGTTGGGTCATAGGGTATCAAATGCCTATACTATTAGAACCAGACCAATGAAGAATGTGCAGCCTTTGCAGGTGGTTTGCATGGACTATCCCAGACCAGAGCTTGAGAGTACTGTCAATTATTTGGAAGCTGCTTACTTATCTTCATCCTTTCGTACTTCTCCTCGTCCAGATAAACCATTAAAGGTCGTAATTGCTGGTGCAGGTTTGGCTGGTTTGTCAACTGCAAAATATTTGGCAGATGCAGGCCATAAACCCATATTGTTGGAAGCAAGAGATGTTTTAGGTGGAAAGGTGGCTGCGTGGAAAGATGATGATGGAGACTGGTATGAGACTGGCTTACATATATTCTTTGGCGCCTACCCAAATGTCCAGAACCTGTTTGGAGAACTTGGTCTAAATGATCGGTTGCAGTGGAAAGAACATTCTATGATATTTGCAATGCCAAACAAGCCAGGGGAGTTCAGTCGATTTGACTTCCTTGACATTCTACCAGCACCACTGAACGGGATATGGGCTATCTTAAAGAACAATGAAATGCTTACTTGGCCAGAGAAAATAAAGTTTGCAATTGGACTACTGCCGGCAATGGTCGGTGGACAGGCTTATGTTGAAGCTCAAGATGGTTTAACTGTGAAAGACTGGATGAAGAAACAAGGTGTACCAGATCGAGTAACTACTGAGGTGTTTATTGCCATGTCAAAGGCATTAAACTTCATAAACCCTGATGAACTTTCCATGCAGTGTATTTTGATTGCCTTAAACCGGTTTCTTCAGGAAAAGCATGGTTCGAAAATGGCATTTTTGGATGGTAATCCCCCAGAGAGACTTTGCCTGCCAATTGTCGATCACATTCGGTCACTAGGCGGTGAAGTCCGACTTAATTCTCGAATTCAAAAGATTGAGCTGAATAAAGACGGAACTGTGAAGAACTTTTTGCTAAAGAACGGTAATGTTATTGAAGGAGATGTTTACGTTTTTGCCACTCCAGTCGATATCTTGAAGCGTCTTTTGCCCGAAGACTGGAAAGAGGTTCCTTACTTCAGGAAATTGGAGAAATTAGTTGGAGTTCCCGTCATAAATGTTCACATATGGTTCGACAGGAAACTGAGGAACACATACGATCATCTACTTTTTAGCAGAAGTCACCTTCTCAGTGTGTATGCTGACATGTCTGTTACATGCAAGGAATATTACGACCCGCATCGCTCTATGCTGGAATTGGTTTTTGCCCCTGCAGAGGAATGGATCTCAAAAAGTGATCAAGAAATTATTGACGCTACTATGAAGGAGCTCGCAAAACTCTTTCCTGATGAAATTTCTGCAGATCAGAGTAAAGCAAAAATATTGAAGTACCATGTCGCTAAAACACCAAGGTCTGTTTATAAAACTGTCCCAGACTGTGAACCTTGCCGTCCATTACAGAGATCCCCAGTGGAAGGTTTCTATTTGGCAGGTGACTACACAAAACAAAAATATTTGGCTTCAATGGAAGGTGCTGTTCTTTCAGGAAAGTTTTGTGCACAAGCTATTGTACAGGATTACGAATTGCTTGCTTCCCGGAGCCAGAAAAAACTAGCTGAGGCAAGTCTGGTGTAA

***>Glycine soja***

ATGGCCGCTTGTGGCTATATATCTGCTGCCAACTTCAATTATCTCGTTGGCGCCAGAAACATATCCAAATTCGCTTCTTCAGACGCCACAATTTCGTTTTCATTTGGCGGGAGCGACTCAATGGGTCTTACTTTGCGACCCGCTCCGATTCGTGCTCCTAAGAGGAACCATTTCTCTCCCTTGCGTGTCGTTTGCGTCGATTATCCACGCCCGGAGCTCGAAAACACCGTTAATTTCGTTGAAGCTGCTTACTTGTCTTCCACCTTTCGTGCTTCTCCGCGTCCTCTAAAACCCTTGAACATCGTTATTGCCGGTGCAGGATTGGCTGGTTTATCAACTGCAAAATATTTGGCTGATGCTGGGCATAAACCTATATTGCTGGAAGCAAGAGACGTTCTAGGTGGAAAGGTTGCTGCATGGAAAGACAAGGATGGAGACTGGTACGAGACAGGCCTACACATCTTTTTTGGGGCTTACCCTAATGTGCAGAACCTTTTTGGAGAACTTGGCATTAATGATCGGTTACAATGGAAAGAGCATTCTATGATTTTTGCTATGCCAAATAAGCCTGGAGAGTTTAGTCGATTTGATTTTCCTGAAGTTCTTCCCTCCCCATTGAATGGAATATGGGCAATATTGAGGAACAATGAGATGCTTACATGGCCAGAGAAAGTAAAATTTGCAATTGGGCTTCTCCCAGCTATGCTTGGCGGACAGCCATATGTTGAGGCTCAAGATGGTCTTTCTGTTCAAGAATGGATGAAAAAGCAGGGCGTACCTGAACGGGTAACTGATGAGGTGTTCATAGCAATGTCTAAGGCACTAAACTTCATCAATCCTGATGAACTTTCAATGCAATGTATATTGATTGCTTTAAACCGATTTCTTCAGGAGAAACATGGTTCTAAGATGGCCTTTTTGGATGGCAATCCACCCGAAAGACTTTGTATGCCGATAGTTGATCATATTCAGTCCTTGGGTGGTGAAGTTCATCTAAATTCGCGCATTCAAAAAATTGAGCTAAATGATGATGGAACGGTGAAGAGCTTCTTACTAAATAATGGGAAAGTGATGGAAGGGGATGCTTATGTGTTTGCAACTCCAGTGGATATTCTGAAGCTTCTTCTACCAGATAACTGGAAAGGGATTCCATATTTCCAGAGATTGGATAAATTAGTTGGCGTCCCAGTCATAAATGTTCACATATGGTTTGACAGAAAACTGAAGAACACATATGATCACCTTCTCTTTAGCAGAAGTCCCCTTCTGAGTGTATATGCTGACATGTCAGTAACTTGCAAGGAATATTATAGCCCAAACCAGTCAATGTTAGAGTTGGTTTTTGCACCAGCCGAAGAATGGATTTCACGTAGTGACGATGATATTATTCAAGCCACGATGACTGAGCTTGCCAAACTCTTTCCTGATGAAATTTCTGCAGACCAAAGCAAAGCTAAGATTCTCAAGTACCATGTTGTTAAAACACCAAGGTCGGTTTACAAAACTGTTCCAAATTGTGAACCTTGTCGACCCATTCAAAGATCTCCTATAGAAGGTTTCTATTTAGCTGGAGATTACACAAAACAAAAATATTTAGCTTCAATGGAAGGCGCTGTTCTTTCTGGGAAGCTTTGTGCACAGGCTATTGTACAGGATTCTGAGCTACTAGCTACTCGGGGCCAGAAAAGAATGGCTAAAGCAAGTGTTGTGTAA

***>Glycine max***

ATGGCCGCTTGTGGCTATATATCTGCTGCCAACTTCAATTATCTCGTTGGCGCCAGAAACATATCCAAATTCGCTTCTTCAGACGCCACAATTTCGTTTTCATTTGGCGGGAGCGACTCAATGGGTCTTACTTTGCGACCCGCTCCGATTCGTGCTCCTAAGAGGAACCATTTCTCTCCCTTGCGTGTCGTTTGCGTCGATTATCCACGCCCGGAGCTCGAAAACACCGTTAATTTCGTTGAAGCTGCTTACTTGTCTTCCACCTTTCGTGCTTCTCCGCGTCCTCTAAAACCCTTGAACATCGTTATTGCCGGTGCAGGATTGGCTGGTTTATCAACTGCAAAATATTTGGCTGATGCTGGGCATAAACCTATATTGCTGGAAGCAAGAGACGTTCTAGGTGGAAAGGTTGCTGCATGGAAAGACAAGGATGGAGACTGGTACGAGACAGGCCTACACATCTTTTTTGGGGCTTACCCTAATGTGCAGAACCTTTTTGGAGAACTTGGCATTAATGATCGGTTACAATGGAAAGAGCATTCTATGATTTTTGCTATGCCAAATAAGCCTGGAGAGTTTAGTCGATTTGATTTTCCTGAAGTTCTTCCCTCCCCATTGAATGGAATATGGGCAATATTGAGGAACAATGAGATGCTTACATGGCCAGAGAAAGTAAAATTTGCAATTGGGCTTCTCCCAGCTATGCTTGGCGGACAGCCATATGTTGAGGCTCAAGATGGTCTTTCTGTTCAAGAATGGATGAAAAAGCAGGGCGTACCTGAACGGGTAACTGATGAGGTGTTCATAGCAATGTCTAAGGCACTAAACTTCATCAATCCTGATGAACTTTCAATGCAATGTATATTGATTGCTTTAAACCGATTTCTTCAGGAGAAACATGGTTCTAAGATGGCCTTTTTGGATGGCAATCCACCCGAAAGACTTTGTATGCCGATAGTTGATCATATTCAGTCCTTGGGTGGTGAAGTTCATCTAAATTCGCGCATTCAAAAAATTGAGCTAAATGATGATGGAACGGTGAAGAGCTTCTTACTAAATAATGGGAAAGTGATGGAAGGGGATGCTTATGTGTTTGCAACTCCAGTGGATATTCTGAAGCTTCTTCTACCAGATAACTGGAAAGGGATTCCATATTTCCAGAGATTGGATAAATTAGTTGGCGTCCCAGTCATAAATGTTCACATATGGTTTGACAGAAAACTGAAGAACACATATGATCACCTTCTCTTTAGCAGAAGTCCCCTTCTGAGTGTATATGCTGACATGTCAGTAACTTGCAAGGAATATTATAGCCCAAACCAGTCAATGTTAGAGTTGGTTTTTGCACCAGCCGAAGAATGGATTTCACGTAGTGACGATGATATTATTCAAGCCACGATGACTGAGCTTGCCAAACTCTTTCCTGATGAAATTTCTGCAGACCAAAGCAAAGCTAAGATTCTCAAGTACCATGTTGTTAAAACACCAAGGTCGGTTTACAAAACTGTTCCAAATTGTGAACCTTGTCGACCCATTCAAAGATCTCCTATAGAAGGTTTCTATTTAGCTGGAGATTACACAAAACAAAAATATTTAGCTTCAATGGAAGGCGCTGTTCTTTCTGGGAAGCTTTGTGCACAGGCTATTGTACAGGATTCTGAGCTACTAGCTACTCGGGGCCAGAAAAGAATGGCTAAAGCAAGTGTTGTGTAA

***>Hibiscus syriacus***

ATGAGTCTCTGTGGGAGTGTTTCTGCCTTAAACTTAAACTTCAAAAGCAACAAGATAAGCATGGGAAGAGTCTTAGCTTTTAGAAGTGGTGAATCCATGGGAAACACCTTGAGAATTCCATTAAAAAAGAGGTCATGTAAGGGTGCTTGTCCTTTGCAGGTAGTTTGCATAGATTATCCAAGGCCAGAGCTTGAGAATACTGTTAATTTTCTGGAGGCTGCCTCTCTATCCGCTTATTTACGTTCTGCTCCTCGTCCAACTAAGCCATTGAAAGTCATAGTTGCGGGTGCAGGTTTGGCTGGTTTGTCAACGGCAAAGTATCTAGCGGATGCAGGTCATAAACCAATATTATTGGAAGCAAGAGATGTTCTTGGCGGAAAGGTGGCTGCATGGAAAGATGATGATGGAGATTGGTATGAGACAGGCCTACATATATTCTTCGGGGCGTACCCGAATGTGCAAAACTTGTTTGGAGAACTTGGCATCAATGATCGGCTGCAATGGAAGGAGCATTCTATGATATTTGCAATGCCAAATAAACCTGGAGAGTTCAGTCGATTTGATTTTCCAGAAGTTCTACCTGCACCCTTAAATGGGATATGGGCCATTTTGAAGAACAATGAAATGCTGACTTGGCCAGAGAAAGTGAAATTTGCAATAGGACTTCTACCTGCAATGCTTGGTGGACAACCTTATGTTGAGGCCCAAGATGGTTTATCTGTTAAAGAGTGGATGAGAAAGCAGGGTGTACCTGATCGTGTCACTGAAGAGGTGTTTATTGCCATGTCAAAGGCTCTGAACTTCATTAACCCTGACGAACTTTCAATGCAGTGTATTCTGATTGCTTTGAATCGATTTCTTCAGGAAAAGCATGGATCAAAGATGGCTTTCTTGGATGGCAACCCTCCGGAGAGGCTTTGCATGCCAATCGTCAATCATATTGAATCACTGGGTGGTGAAGTTCGGCTTAACTCACGGTTAAAGAAAATAGAGCTCAATGCTGATGGAACTGTGAAAAGTTTTCTTCTAAATAATGGCAATATAATTGAAGGAGATGCTTATGTAGTTGCAACTCCAGTTGATATCTTCAAGTTACTTTTGCCTGAAGACTGGAGAGAAATTTCATACTTCAAGAAATTAGATAAATTAGTTGGAGTTCCAGTTATCAACGTTCACATCTGGTTTGATAGGAAATTGAAGAACACCTATGATCATCTACTGTTCAGCAGAAGTTCGCTTCTAAGTGTTTATGCTGACATGTCTGTAACGTGTAAGGAATATTACAATCCGAACCAATCCATGTTAGAGTTAGTTTTTGCCCCGGCAGAAGAATGGATTGCACAAAGTGATTCGGAAATTATTGATGCTACAATGAAGGAGCTTGCAAAGCTCTTCCCTGATGAAATATCTGCAGATCAGAGTAAAGCAAAAGTTGTAAAGTACCATATCGTTAAAACACCAAGATCAGTATATAAAACTGTTCCAAATTGTGAACCCTGCCGTCCCGTGCAAAGATCTCCGATACAGGGGTTCTATCTAGCAGGTGATTACACAAAGCAAAAGTATTTAGCTTCCATGGAAGGAGCTGTCCTCTCAGGGAAGTTTTGTGCACAGTCTATTGTACAGGATTATGAGTTGCTTCAAACCCTGGGACAAAGAAAGTTGACCGAAGCAAGCATTCACTGA

***>Arachis hypogaea***

ATGGCCACGTGTATATCTGCTGTGAACTTGAATTACCAAATTGCCCCAAGAACCGTTTCGAAATTCAGTTCTGCGACGAGCTCGGACCAAACGGCGTCGTTTTCGCTTGGCGCGAGCGAGTCGATGGGACCGAGTCTCAGACTCGCTTTGACTCGTGCTGCTAAGAGCACCACCACCACCACCACTAGGTTGTTGAGGAAGAAGCATGGCTCTCTTCCGTTGCGAGTGTTTTGCGTCGATTACCCTCGGCCGGAGCTTGAGAACACCGTGAATTTCCTCGAAGCAGCGTTCTTGTCTTCGACTTTTCGTGATTCACCACGACCAGCGAAACCGTTGAAGGTCGTTGTTGCTGGAGCAGGATTGGCTGGTTTATCGACTGCAAAATATTTGGCAGATGCTGGTCACAAGCCTGTATTACTGGAGGCAAGAGATGTTCTAGGTGGAAAGGTTGCTGCATGGCAAGATGAAGATGGAGACTGGTATGAGACAGGCCTGCATATATTCTTTGGGGCATACCCTAATGTGCAGAATCTATTTGGAGAACTTGGTATTAATGATCGGTTACAATGGAAGGAACATTCTATGATTTTTGCAATGCCAAGTAAACCTGGAGAATTTAGTCGATTTGATTTTCCTGAAGCCCTACCAGCTCCACTAAATGGAATATTGGCAATATTGAGGAACAATGAGATGCTTACTTGGCCAGAAAAAGTCAAGTTTGCAATTGGGCTTCTGCCAGCTATGCTTGGTGGTCAGTCTTATGTTGAAGCTCAAGATGGCCTTTCTGTTAAAGATTGGATGAGAAAGCAGGGTGTACCTGATCGAGTAACTGATGAGGTGTTCATAGCCATGTCAAAGGCACTAAATTTCATCAACCCGGATGAACTTTCAATGCAATGTATATTGATTGCCTTAAACCGATTTCTTCAGGAAAAGCATGGTTCTAAAATGGCCTTTTTGGATGGTAATCCCCCTGAAAGACTTTGTATGCCTATTGTTGATCATATTCAATCCTTGGGTGGTGAAGTTCATCTAAATTCTCGCATTCAAAAGATCGATCTAAATGATGATGGCACTGTGAAGAGCTTCTTGCTAAATAATGGGAAGGTGATTGAAGGGGATGCTTATGTGTTTGCAACTCCAGTTGATATTCTGAAGCTTCTTGTGCCTAACAATTGGAAAGAGATTCCATATTTCCAAAGATTGGAGAAACTAGTAGGAGTCCCGGTTATAAATGTTCATATATGGTTTGACAGAAAACTGAAGAACACATATGATCATCTTCTCTTCAGCAGAAGTCCACTTTTGAGTGTATATGCTGACATGTCAGTAACTTGTAAGGAATATTATAACCCAAACCAGTCTATGTTGGAGTTGGTTTTTGCACCTGCTGAAGAATGGGTTTCTCGAAGTGATGAAGACATCATTGCTGCTACGATGTCTGAACTTGCCAAACTGTTCCCTGATGAAATTTGTGCAGACCAGACAAAAGCAAAGATTGTTAAGTACCATGTTGTTAAAACACCCAGGTCGGTTTACAAAACTGTTCCAAATTGTGAACCTTGTCGTCCCATACAACGATCTCCTATAGAAGGTTTCTATTTAGCTGGAGATTACACAAAACAAAAATATTTAGCTTCAATGGAAGGTGCTGTTCTGTCAGGGAAGCTATGTGCACAGGCTATTGTACAGGATTCTGAGCTACTTGCTGCTCGGAGCCAGAAAGCTGTGGCCCAAGCAAGTGTTATTTAA

***>Castanea sativa***

GACAGGAAATCAAACTCTGAGATGTGGGTTTCTTAATAATTCGGTGAAAACCAATGCATTAGCATTTGGAGGTTGTGAATCCATGGGTCATATTTtGAGAATTCCACATACAAAGGCTATTAGATTGAGGCCGAGGAAGGGTGTCTCTCCTTTGCAGGTAGTATGTATGGACTTTCCAAGACCAGAGCTTGAGAATACTGTTAATTTCTTAGAGGCTGCTTATTTGTCTTCTTCCCTCCGTGCATCTGCTCGTCCATCTAAaCCCCTAACAGTTGTAATTGCTGGTGCAGGTTTGGCTGGTTTGTCTACTGCAAAGTATTTGGCAGATGCTGGTCACAAACCTATACTATTGGAGTCAAGAGATGTGCTAggaGGAaaGGTGGCTGCATGGAAAGATGACGATGGAGActGGTATGAGACTGGATTACATATATTCTTTGGGGCTTACCCAAATGTGCAGAATCTTTTtGGAGAACTTGGTATTGATGATCGGTTGCAATGGAaGGAACATTCTATGATTTTtGCAATGCCAAATAAGCCGGGAGAGTTCAGCCGATTTGATTTTCCTGAAGTTCTTCCTGCACCATTAAATGGAATATGGGCTATTTTGAAGAACAATGAGATGCTGACTTGGCCAGATAAAGTCAAGTTTGCAATTGGACTCTTGCCAGCAATGCTTGGTGGACAGGCTTATGTTGAAGCACAAGATGGTTTAACTGTTAAAGAGTGGATGAGAAAGCAGGGAGTACCTGATCGTGTAACTGATGAGGTGTTTGTAGCCATGTCAAAGGCGCTAAACTTCATTAACCCTGATGAACTTTCAATGCAATGCATATTGATTGCTTTGAATAGGTTTCTTCAGGAGAAGAATGGTTCCAAGATGGCTTTCTTGGATGGTAATCCCCCAGAGAGACTCTGTATGCCAATTGTTGATCATATTCAATCACTCGGcGGTGAAGTAAGACTGAATTCGAGAATACAAAAAATCGAGCTAAATAATGATGGAACAGTGAAAAGCTTTTTACtGAATAATGGGAACATGATTGAAGGAGATGCTTATGTATTtgCTaCTCCAGTTGATATCCTGAAGCTTCTTTTGCCGGAAAACTGGAAAGAGATTCCATATTTtCAGAGATTAAAGAAATTAGTTGGAGTTCCAGTTATTAATGTCCACATaTGGTTTGACAGAAAACTGAAGAACACCTATGATCACCTACTGTTTAGCAGAAGTCcACTTCTCAGTGTGTATGCTGACATGTCACTAACgTGTAAGGAATATTACAACCCAAACcAATCTATGCTGGAGTTGGTTTTTGCgCCTGCAGAAGAATGGATTTCATGCAGTGACTCAGACATTATTGACGCTACAATGAATGAACTTGCAAGACTCTTTCCCGATGAAATTTCCACGgATCAAAGCAAAGCAAAGATTGTGAAGTACCATGTTGTTAAAACACCAAGGTCTGTTTACAAAATTGTCCCAGACTGTGAACCTTGC

***>Arabidopsis thaliana***

ATGGTTGTGTTTGGGAATGTTTCTGCGGCGAATTTGCCTTATCAAAACGGGTTTTTGGAGGCACTTTCTTCATCTGGAGGTATTGAATTGATGGGACACAGCTTTAGGGTTCCGACTTTTCAAGCACCTAAGACAAGAACAAGGAGGAGAAGTACTGCTGGTCCTTTGCAGGTAGTTTGTGTGGATATTCCAAGGCCAGAGCTAGAGAACACTGTCAATTTCTTGGAAGCTGCTAGTTTATCTGCATCTTTCCGTAGTGCTCCTCGTCCTGCAAAGCCTTTAAAAGTTGTAATTGCTGGTGCTGGATTGGCTGGATTGTCAACTGCAAAGTACCTGGCTGATGCCGGCCACAAACCTCTGTTGCTTGAAGCAAGAGATGTTCTTGGTGGAAAGATAGCTGCATGGAAGGATGAAGATGGGGACTGGTATGAAACTGGTTTACATATATTCTTCGGTGCTTATCCAAATGTGCAGAACTTATTTGGAGAACTTGGGATCAATGATCGGTTGCAGTGGAAGGAACACTCCATGATTTTCGCTATGCCAAGTAAACCTGGAGAGTTTAGTAGATTTGACTTCCCAGATGTCCTACCAGCACCCTTAAACGGTATTTGGGCTATTTTGCGGAACAACGAGATGCTGACATGGCCAGAGAAAATAAAGTTTGCTATTGGACTTTTGCCAGCCATGGTCGGCGGCCAGGCTTATGTTGAAGCCCAAGATGGCTTATCAGTCAAAGAATGGATGGAAAAGCAGGGAGTACCTGAGCGTGTGACTGACGAGGTGTTTATTGCCATGTCAAAGGCACTAAACTTTATCAACCCTGATGAACTGTCAATGCAATGCATTTTGATAGCTTTGAACCGGTTTCTTCAGGAAAAACATGGTTCCAAGATGGCATTCTTGGATGGTAATCCTCCGGAAAGGCTTTGTATGCCAATAGTGGATCATATTCGATCACTAGGTGGGGAAGTGCAACTTAATTCTAGGATAAAGAAAATTGAGCTCAATAACGATGGCACGGTTAAGAGTTTCTTACTAACTAATGGAAGCACTGTCGAAGGAGACGCTTATGTGTTTGCCGCTCCAGTCGATATCCTGAAGCTCCTTTTACCAGATCCCTGGAAAGAAATACCGTACTTCAAGAAATTGGATAAATTAGTTGGAGTGCCAGTTATTAATGTTCATATATGGTTTGATCGAAAACTGAAGAACACATATGATCACCTACTCTTTAGCAGAAGTAACCTTCTTAGCGTGTATGCGGACATGTCCTTAACTTGTAAGGAATATTACGATCCTAACCGGTCGATGCTGGAGCTAGTATTTGCACCAGCAGAGGAGTGGATATCACGGACTGACTCTGACATCATAGATGCAACAATGAAAGAACTCGAGAAACTCTTCCCTGATGAAATCTCAGCTGACCAAAGCAAAGCTAAAATCCTGAAGTACCATGTCGTTAAAACACCAAGGTCTGTGTATAAGACCATCCCAAACTGTGAACCATGTCGTCCTCTACAGAGATCACCTATTGAAGGATTCTACTTAGCTGGAGATTACACAAAACAGAAGTACTTAGCTTCCATGGAAGGCGCGGTTCTCTCTGGCAAATTCTGCTCACAGTCTATTGTGCAGGATTACGAGCTACTGGCTGCATCTGGATCGAGAAAGTTATCGGAGGCAACAGTATCATCATCATCATCATGA

***>Solanum lycopersicum***

ATGCCGCCGTGTCTCTGCCTTCCCGCCACTCTCCCTCACCCTTCCTCAACTCTTTTCTCCACGCGCCGCCGCTTCCCACTACTAAAACCGCCGTATGCTTCCTCAAATATACCCGTTGGCTCAACTTCTCAGCCTAATACCACCGGAGTCATTGTCATCGGCGGCGGTTTAGCTGGCCTAGCAGCTGCCATTCGGCTTCAAGCTGACAACATCCCCTTTCTCCTTCTCGAAGCTTCTGATGCCGTTGGTGGTCGCGTTCGGTCCGATGTAGTTGACGGATATACCCTTGATCGTGGTTTCCAGATTTTCATTACTGGTTATCCTGAAGCCCGAAAAGTCCTTGATTATGACTCTTTAGACCTCAGAAAATTCTATTCGGGGGCTCAGGTTTACTATGGTGGTCGTTTCCACACCGTTGCTGATCCTCTCCGGCATTTCGCAGATGCACTGCAATCTTTAACTAACCCAATTGGTTCAGTTGTGGATAAATTACTTATAGGATTGACTAGATTGAAAGTTTTGACGCAAGGTGATGATGAAATATTGAGTGCTGATGAAGAACCTACGATGAATTTATTAAAGAAGATCGGTTTTTCTGATGCAATATTGGAAAGGTTCTTTCGACCGTTTTTTGGTGGAATTTTCTTCGATAGAGAGCTTGAAACAACGTCGCGGCTGTTCAATTTCATCTTCAAATGCCTAGCTCTTGGTGACAACACACTTCCGGCGAAGGGCATTGCGGCCATTCCGGAACAGTTGGCGGCAAAACTGCCGTCGAATTCAATATTGTTCAATACACGTGTTGTTTCCGTTGATTCAGGATCGGATTCCAGCACAAAAATAAGAGTGACACTACAAAATGGGGAAATGTTGGAAAGTGAATTTGGGGTAATATTGGCAGTTGAAGAGCCTGAAGCTGTCAAGTTGTTGGCGGGAGAAAAAACCGGTGAGGTTCGGCAACCGGTTCGGAGCACAGTATGTTTGTATTTTTCAGCTGACCAAGGCAAAGTTCCGGTGCAGGATCCGGTTCTTCTTCTTAACGGATCGGGTAAAGGTATCGTTAATAACATGTTCTTCGCGACCAATGTGGCTCCCTCTTATGCTCCGGCAGGGAAGGCACTGGTTTCTGTCACGCTTGTGGGGCTTTATGGTGATGTGGCAGATGAGGATTTGGTGGATCGGGTCGTGAAGGAGCTATCGGGTTGGTTTGGGGAGTCGGTAGTTGGGTCATGGGGTTACTTGAGGATGTACAGGATCGGGTTTGCCCAACCGAACCAATGCCCACCCACTAACTTGAAGAAGAACCCGAAAGTGAAACCGGGCTTGTACATTTGTGGAGATTATGTGACTAGCGCTACTTTTGATGGAGCTTTAGTTTCTGGGAAAAAAGCAGCAGAAACTTTGTTACAAGATAAAGCTCTGGTTATTGTATAG

***>Nicotiana tabacum***

ATGCCCCAAATTGGACTTGTTTCTGCCGTTAATTTGAGAGTCCAAGGTAATTCAGCTTATCTTTGGAGCTCGAGGTCTTCTTTGGGAACTGAAAGTCAAGATGGTCACTTGCAAAGGAATTTGTTATGTTTTGGTAGTAGCGACTCCATGGGGCATAAGTTAAGGATTCGTACTCCCAGTGCCATGACCAGAAGATTGACAAAGGACTTTAATCCTTTAAAGGTAGTCTGCATTGATTATCCAAGACCAGAGCTAGACAATACAGTTAACTATTTGGAGGCGGCGTTATTATCATCATCATTTCGTACTTCCTCACGCCCAACTAAACCATTGGAGATTGTTATTGCTGGTGCAGGTTTGGGTGGTTTGTCTACAGCAAAATATCTGGCTGATGCTGGTCACAAACCGATATTGCTGGAGGCAAGAGATGTCCTAGGTGGAAAGGTAGCTGCATGGAAAGATGATGATGGAGATTGGTATGAGACTGGGTTGCACATATTCTTTGGGGCTTACCCAAATATGCAGAACTTGTTTGGAGAACTAGGGATAAACGATCGGTTGCAGTGGAAGGAACATTCAATGATATTTGCGATGCCTAACAAGCCAGGGGAGTTCAGCCGCTTTGATTTTCCTGAAGCTCTTCCTGCGCCATTAAATGGAATTTTGGCCATACTAAAGAACAACGAAATGCTTACGTGGCCCGAAAAAGTCAAATTTGCTATTGGACTCTTGCCAGCAATGCTTGGAGGGCAATCTTATGTTGAAGCTCAAGACGGTTTAAGTGTTAAGGACTGGATGAGAAAGCAAGGTGTGCCTGATAGGGTGACAGATGAGGTGTTCATTGCCATGTCAAAGGCACTTAACTTCATAAACCCTGACGAGCTTTCGATGCAGTGCATTTTGATTGCTTTGAACAGATTTCTTCAGGAGAAACATGGTTCAAAAATGGCCTTTTTAGATGGTAACCCTCCTGAGAGACTTTGCATGCCGATTGTTGAACATATTGAGTCAAAAGGTGGCCAAGTCAGACTAAACTCACGAATAAAAAAGATTGAGCTGAATGAGGATGGAAGTGTCAAATGTTTTATACTGAATAATGGCAGTACAATTAAAGGAGATGCTTTTGTGTTTGCCACTCCAGTGGATATCTTCAAGCTTCTTTTGCCTGAAGAGTGGAAAGAGATCCCATATTTCCAAAAGTTGGAGAAGCTAGTGGGAGTTCCTGTGATAAATGTCCATATATGGTTTGACAGAAAACTGAAGAACACATCTGATAATCTGCTCTTCAGCAGAAGCCCATTGCTCAGTGTGTATGCTGACATGTCTGTTACATGTAAGGAATATTACAACCCCAATCAGTCTATGTTGGAATTGGTATTTGCACCTGCAGAAGAGTGGATAAATCGTAGTGACTCAGAAATTATTGATGCTACAATGAAGGAACTAGCAAAGCTTTTCCCTGACGAAATTTCGGCAGATCAGAGCAAAGCAAAAATATTGAAGTATCACATTGTCAAAACTCCAAGGTCTGTTTATAAAACTGTGCCAGGTTGTGAACCCTGTCGGCCCTTGCAAAGATCTCCTATTGAGGGGTTTTATTTAGCTGGTGACTACACAAAACAGAAATACTTGGCTTCAATGGAAGGTGCTGTCTTATCAGGAAAGCTTTGTGCCCAAGCTATTGTACAGGATTACGAGTTACTTCTTGGCCGGAGCCAGAAGAAGTTGGCAGAAGCAAGCGTAGTTTAG

***>Vaccinium myrtillus***

ATGAAGAACGTGCAGCCTTTGCAGGTGGTCTGCATGGATTATCCTAGACCAGAGCTTGAGAGTACTGTCAGTTATTTGGAAGCTGCTTACATATCTTCATCCTTTCGTTCTTCTCCTCGTCCAGATAAGCCATTGAAGGTCGTCATTGCCGGTGCAGGTTTGGCTGGTTTGTCAACTGCAAAATATTTGGCAGATGCAGGCCATAAACCGATATTGCTGGAAGCAAGAGACGTTTTAGGCGGAAAGGTGGCTGCATGGCAAGATGATGATGGAGACTGGTATGAGACTGGCTTACATATATTTTTTGGCGCCTACCCAAATGTCCAGAACCTGTTTGGAGAACTTGGTATAAATGATCGGTTGCAGTGGAAAGAACATTCTATGATATTTGCAATGCCAAACAAGCCAGGGGAGTTCAGTCGATTTGACTTCCTTGACATTCTACCGGCACCATTAAACGGGATATGGGCCATCTTAAAGAATAATGAAATGCTTACTTGGCCGGAGAAAGTAAAATTTGCGATTGGACTACTGCCAGCAATGATCGGTGGACAGGCGTATGTTGAAGCTCAAGATGGTTTAACTGTGAAAGACTGGATGAGGAAACAAGGTGTACCAGATCGAGTAACTACTGAGGTGTTTATTGCCATGTCAAAGGCATTGAACTTCATAAACCCTGATGAACTTTCCATGCAGTGTATTTTGATTGCATTGAACCGGTTTCTCCAGGAAAAGCATGGTTCAAAAATGGCATTCTTGGATGGTAACCCTCCAGAGAGACTTTGCCTGCCAATTGTCAATCATATTCAGTCCCTAGGTGGTGAAGTCCGGCTTAATTCTCGAATTCAAAAGATTGAGCTGAATAAAGATGGAACCGTGAAGAACTTCTTACTAAATAATGGAAATGTTGTTGAAGGAGATGTTTATGTTTTTGCCACTCCAGTCGATACCTTGAAGCTTCTTTTGCCTGAAGACTGGAAAGAGATTCCTTACTTCAGAAAATTGGAGAAATTAGTTGGAGTTCCTGTTATTAATGTCCACATATGGTTTGACAGGAAACTGAAGAACACGTACGATCATCTACTCTTTAGCAGAAGTCCGCTTCTCAGTGTGTATGCTGACATGTCTGTGACATGCAAGGAATATTACGACCCAAATCGCTCTATGCTGGAATTGGTTTTCGCACCTGCAGAGGAATGGATCTCAAAAAGTGATCAAGAAATTATTGACGCTACGATGATGGAGCTCGCAAAACTCTTTCCTGATGAAATTTCTGCTGATCATAGTAAAGCAAAAATATTGAAGTACCATGTAGTTAAAACACCAAGGTCTGTTTATAAAACTGTCCCGGACTGTGAACCTTGCCGTCCCTTACAAAGATCCCCAGTGGAAGGTTTCTATTTGGCCGGTGACTACACAAAACAAAAATATTTGGCTTCAATGGAAGGTGCTGTTCTTTCAGGAAAGTTTTGTGCACAAGCTATTGTACAGGATTACGAGTTGCTTGCTTCTCGGGGGCAGAAAAAGCTAGCTGAGGCAAATCCTCTTTTTGCACTCATGGCTGAGCTAAAGCAACAACAGTCAAAGAGTACTAGAGCCAATGCCTCTTCAAAGAGAAAGGGCAATGGGTTCACAAGGAAGTGTGCTTCCTTGGTTAAGGAACAAAGAGCTAGACTTTACATACTCCGCCGCTGCGCCACCATGCTTCTCTGCTCCTACATACAAGGAGATGACTAA
